# A new paradigm for outer membrane protein biogenesis in the Bacteroidota

**DOI:** 10.1038/s41586-025-09532-8

**Published:** 2025-10-01

**Authors:** Xiaolong Liu, Luis Orenday Tapia, Justin C. Deme, Susan M. Lea, Ben C. Berks

**Affiliations:** 1 Department of Biochemistry, University of Oxford, Oxford, UK; 2 Center for Structural Biology, Center for Cancer Research, National Cancer Institute, Frederick, MD USA; 3 Structural Biology, St Jude Children’s Research Hospital, Memphis, TN USA

**Keywords:** Bacterial secretion, Cryoelectron microscopy, Bacterial secretion

## Abstract

In Gram-negative bacteria, the outer membrane is the first line of defence against antimicrobial agents and immunological attacks^1^. A key part of outer membrane biogenesis is the insertion of outer membrane proteins by the β-barrel-assembly machinery (BAM)^2–4^. Here we report the cryo-electron microscopy structure of a BAM complex isolated from *Flavobacterium johnsoniae*, a member of the Bacteroidota, a phylum that includes key human commensals and major anaerobic pathogens. This BAM complex is extensively modified from the canonical *Escherichia coli* system and includes an extracellular canopy that overhangs the substrate folding site and a subunit that inserts into the BAM pore. The novel BamG and BamH subunits that are involved in forming the extracellular canopy are required for BAM function and are conserved across the Bacteroidota, suggesting that they form an essential extension to the canonical BAM core in this phylum. For BamH, isolation of a suppressor mutation enables the separation of its essential and non-essential functions. The need for a highly remodelled and enhanced BAM complex reflects the unusually complex membrane proteins found in the outer membrane of the Bacteroidota.

## Abstract (web summary)

Structural and biochemical studies of the β-barrel-assembly machinery from *Flavobacterium johnsoniae* reveal a subunit composition and assembly that are distinct from those of the canonical *Escherichia coli* complex.

## Main

The well-characterized *E. coli* BAM complex (BAM_*Ec*_) is composed of the outer membrane protein (OMP) BamA together with four periplasmic lipoprotein subunits^5^. Only BamA and the lipoprotein BamD are individually essential for BAM function, and the roles of the remaining subunits remain poorly defined^2^. BamA is a 16-stranded OMP^2^ that is related to the central subunit of the machinery that inserts β-barrel proteins into the mitochondrial outer membrane (OM)^6^. The BamA barrel has a periplasmic extension composed of five polypeptide transport-associated (POTRA) domains to which the lipoprotein subunits bind^7–9^. Within the BamA barrel the seam between the first and last strands is unusually short and can open^7,9^, exposing the N-terminal strand of the BamA barrel to pair with the C-terminal strand of an incoming substrate OMP^10,11^. This structure in turn acts as a template for insertion and folding of successive strands of the nascent OMP through β-augmentation. The result is the formation of a hybrid barrel between BamA and the client OMP that is resolved when the substrate barrel is completed and closes to release it from BamA^3,4^.

The Bacteroidota are a phylum of abundant Gram-negative commensals found in the human gut and other human microbiomes^12^ that includes major opportunistic anaerobic pathogens that are responsible for sepsis (for example, *Prevotella* species and *Bacteroides fragilis*) and severe dental disease (*Porphyromonas gingivalis*). OM proteins in the Bacteroidota exhibit considerably greater structural diversity than the OM proteome of *E. coli*, raising the possibility that the Bacteroidota BAM machinery might be functionally augmented relative to BAM_*Ec*_. Bacteroidota OMPs commonly possess much larger extracellular domains than *E. coli* OMPs^13–15^ and Bacteroidota BAM must be capable of assembling these. Furthermore, and unlike *E. coli*, the Bacteroidota possess abundant cell surface lipoproteins (SLPs), which the BAM complex has been speculated to export^13,16^. Notably, both of these biosynthetic requirements are involved in the assembly of starch utilization system (SUS) nutrient uptake systems, a characteristic and highly abundant feature of the Bacteroidota OM, which consist of a SLP (SusD) and an OMP with large extracellular regions (SusC)^13,17^. A further intriguing aspect of Bacteroidota BAM is a possible functional connection with the Bacteroidota-specific type 9 secretion system (T9SS)^18^ which has two essential components encoded at the *bamA* locus^19^.

To investigate the nature of the Bacteroidota BAM system we isolated and characterized the BAM complex from the T9SS-possessing bacterium *F. johnsoniae*.

## *F. johnsoniae* BAM complex structure

**Fig. 1:**
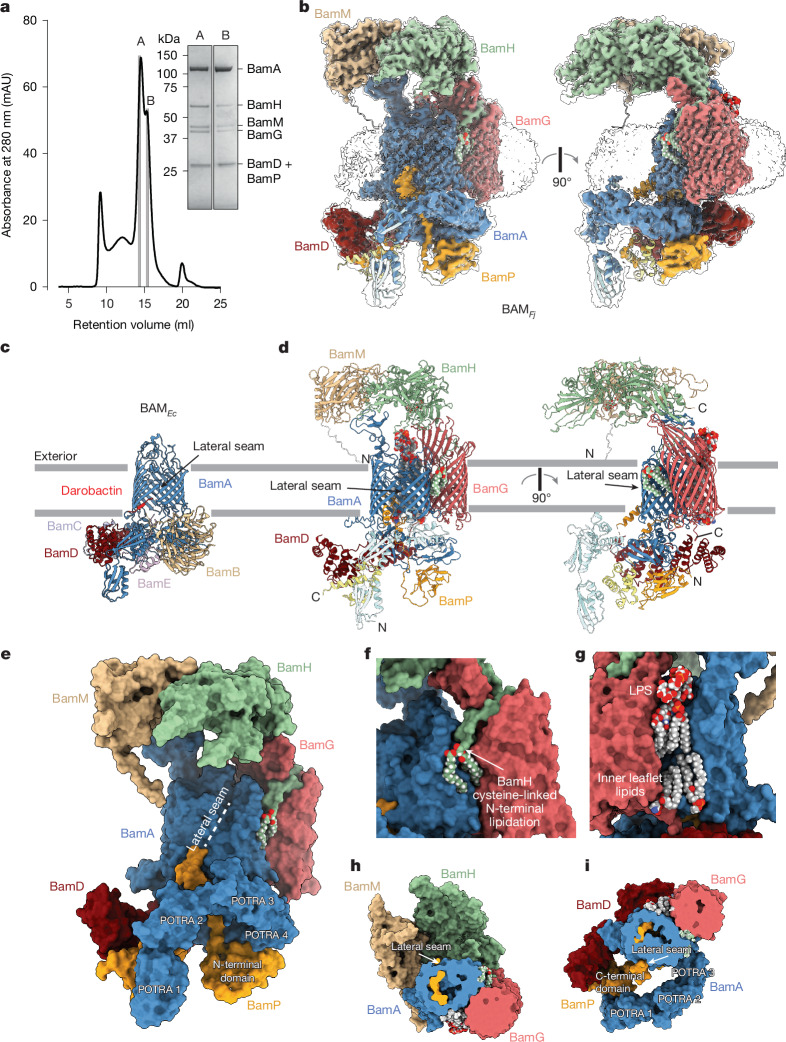
Structure of the BAM_*Fj*_ complex. **a**, Size-exclusion chromatography profile of the purified BAM_*Fj*_ preparation and Coomassie-stained SDS–PAGE gel of the indicated fractions. Bands were identified by peptide fingerprinting. Fraction A was used to determine the structure of the full BAM_*Fj*_ complex and fraction B was used for the structure of the BamAP complex. Similar data were obtained for three independent preparations. **b**, Cryo-electron microscopy (cryo-EM) volume for the BAM_*Fj*_ complex overlaid on the hybrid model shown in **d**. The volume is shown at a high contour level (coloured) and at a low contour level (semi-transparent). **c**,**d**, Comparison of the most similar *E. coli* BAM complex structure (darobactin 9-bound complex; PDB: 8ADI) (**c**) with the BAM_*Fj*_ complex (**d**). Structures are shown in cartoon representation with lipids and metal ions in space-filling atom representation coloured by element. For BAM_*Fj*_, the poorly resolved BamA POTRA 1–3 domains and BamP C-terminal domain are modelled by placing AlphaFold^20^ structures in the electron microscopy density (lighter coloured domains). **e**–**i**, The BAM_*Fj*_ hybrid model (Supplementary Data 1) with protein components in space filling representation and lipids shown as atom spheres coloured by element. **e**, View in the same orientation as **d**, left. **f**, The *N*-acyl and *S*-diacylglyceryl groups attached to the N-terminal cysteine of BamH. **g**, The resolved portion of a lipopolysaccharide (LPS) molecule in the outer leaflet of the OM and two ordered phospholipid molecules on the inner leaflet of the OM. **h**, View from the periplasm with the periplasmic side of the complex cut away to the membrane midpoint. **i**, View from the exterior with the extracellular side of the complex cut away to the membrane midpoint. Source data

**Extended Data Fig. 1:**
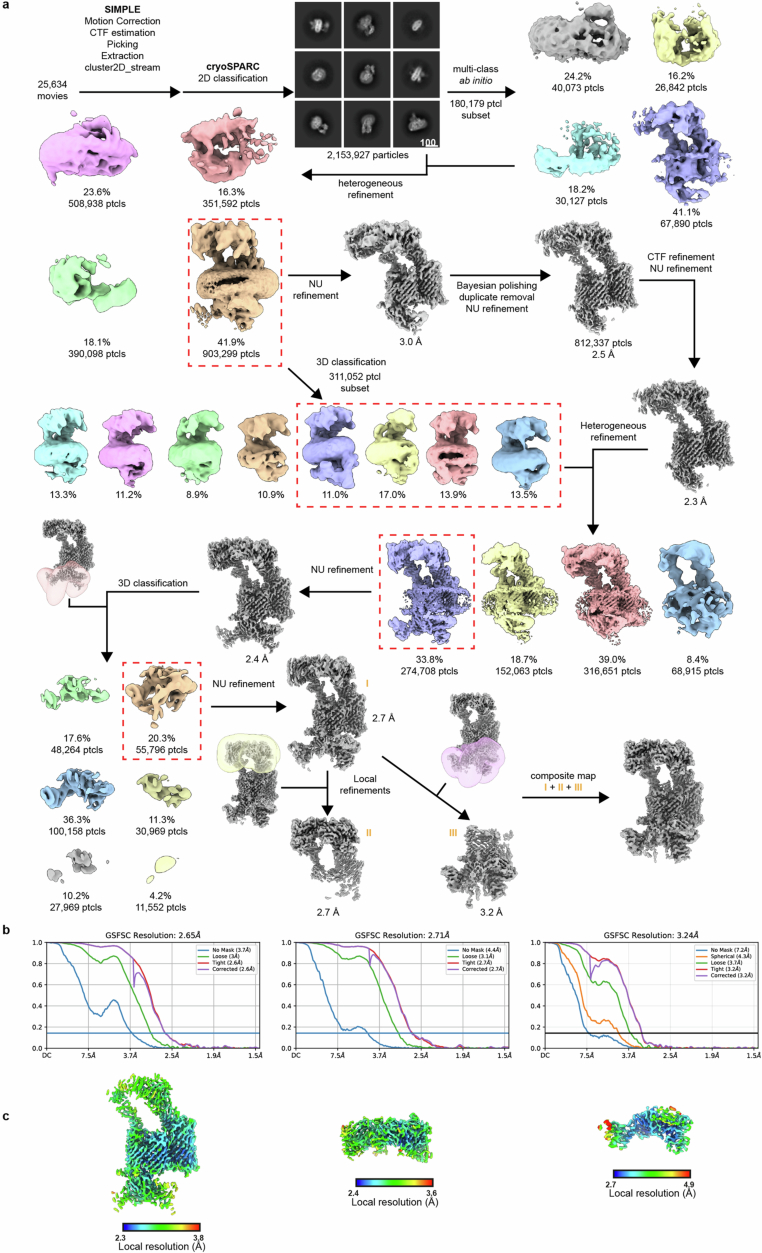
Workflow for the cryoEM analysis of the *F. johnsoniae* BAM_*FJ*_ complex and map quality metrics. **a**, Twin-Strep-tagged BamA complexes were purified by Streptactin affinity chromatography and size exclusion chromatography and the major (highest molecular size) peak was analyzed. See Fig. 1a for corresponding SDS-PAGE analysis of this material. Image processing workflow for the BamA complexes. **b**,**c**, Gold-standard Fourier Shell Correlation (FSC) curves used for global resolution estimation (**b**) and local resolution estimate (**c**) of consensus (left), extracellular (middle), or periplasmic (right) volumes from the BAM_FJ_ complex.

**Extended Data Fig. 2:**
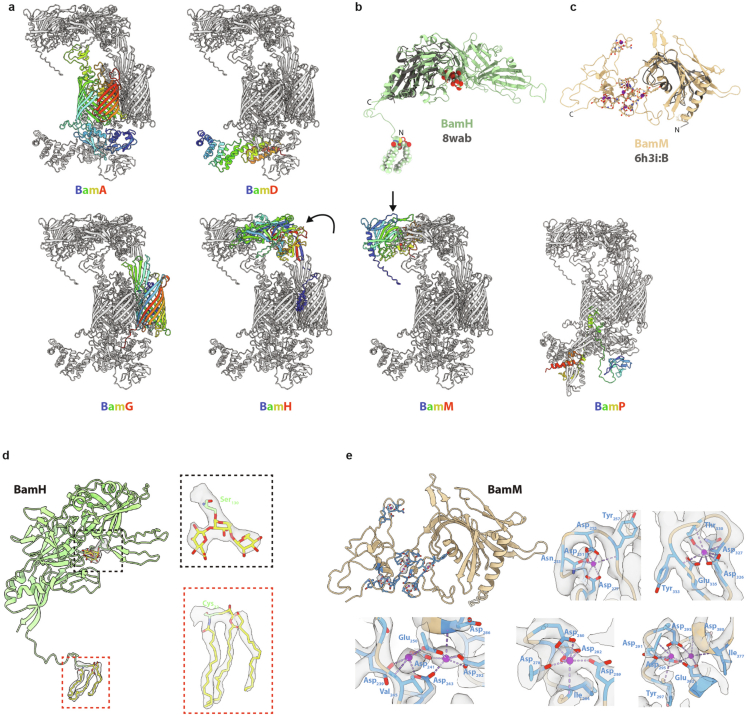
Further structural analysis of the BAM_*FJ*_ complex. **a**, Chain ordering. The indicated subunit in each panel is rainbow-coloured from the N- (blue) to C-terminus (red). **b**, BamH (green) in cartoon representation with ligands (glycosylation and lipidation) as space fill representation. The protein is viewed from the direction indicated in (**a**) and overlaid with the closest structural homologue as judged by PDBeFold 2.58^64^, the chondroitin sulfate-binding carbohydrate binding module of a chondroitinase (dark grey, PDB 8wab, RMSD 2.5 Å across 64 equivalent residues), which is defined as a DNRLRE domain-containing protein by UniProtKB. **c**, BamM (tan) in cartoon representation with bound metals shown as purple spheres and coordinating residues in ball-and-stick representation. The protein is viewed from the direction indicated in (**a**) and overlaid with the closest structural homologue as judged by PDBeFold 2.58^64^, the peptidyl-prolyl isomerases (PPI) subunit (dark grey) from the Type 9 Secretion System translocon complex (PDB: 6h3i chain B, RMSD 0.75 Å across 74 equivalent residues). **d**, Glycosylation and lipidation of BamH shown in ball-and-stick representation within the cryoEM volume in the context of the full chain (left) and in closeup (right). The modelled glycosylation was assigned on the basis of the EM density informed by prior studies of O-glycosylation in Bacteroidota^65^ but without biochemical identification. **e**, Bound metals within BamM modelled as calcium ions (purple spheres) with coordinating residues shown in ball-and-stick representation. The model is displayed within the EM density (insets) or showing just the EM density for the metal ions (full structure). The metals were assigned as Ca ions based on their co-ordination chemistry (O-only ligation, variable co-ordination number and geometry, and appropriate bond lengths) and refining to thermal mobility (B) factors that matched those of the ligating protein atoms.

We isolated the native *F. johnsoniae* BAM complex (BAM_*Fj*_) using an affinity tag fused to BamA (Fjoh_1690). Biochemical (Fig. 1a) and structural (Fig. 1b Extended Data Figs. 1 and 2a and Extended Data Table 1) analysis of the BAM_*Fj*_ complex revealed that it contains five proteins in addition to BamA, one of which could be assigned as BamD (Fjoh_3469). The remaining co-purifying proteins were unrelated to known BAM subunits from other organisms, and did not include T9SS components. We named these novel BAM_*Fj*_ subunits BamG (Fjoh_1412), BamH (Fjoh_0823), BamM (metal ion-containing; Fjoh_0050) and BamP (periplasmic; Fjoh_1771). Smaller BamA-containing complexes present in the sample appear to be fragmentation products (Extended Data Fig. 1).

As in BAM_*Ec*_, BamA forms the core of BAM_*Fj*_, to which the other subunits are directly or indirectly attached (Fig. 1c). However, whereas all the accessory subunits of BAM_*Ec*_ are located in the periplasm (Fig. 1c), BAM_*Fj*_ has a remarkably different organization in which only BamD and BamP are periplasmic or part periplasmic proteins (Fig. 1b,d,e). Uniquely, the BamG subunit is a transmembrane OMP, whereas BamH and BamM are SLPs that together form an extensive extracellular structure. BamG is bound to the ‘rear’ of the BamA barrel relative to the lateral seam. The interaction between BamA and BamG is reinforced by lipid binding on either side of the protein interface. On one side, these interactions are provided by the phospholipid tail of BamH (Fig. 1f) and on the other, they are provided by the ordered lipid portion of a lipopolysaccharide molecule in the outer leaflet of the membrane and two ordered phospholipid molecules in the inner leaflet (Fig. 1g). BamH and BamM interact with each other to form a long canopy structure on the extracellular side of the OM that extends from the rear of BamA across the BamA barrel and out beyond the lateral seam to cover the position in the membrane where client OMPs assemble on BamA (Fig. 1b,d,e,h). The canopy is positioned at an approximately constant height of 40 Å above the inferred position of the membrane bilayer and delineates an approximately 3,000 Å^3^ space above the membrane surface. The canopy is anchored to the BAM_*Fj*_ complex through binding to extracellular ‘pillars’ provided by the BamA and BamG subunits. At the periplasmic side of the membrane the folded domains of the novel BamP subunit are bound to BamD and to the POTRA domains of BamA. These domains are linked by a loop that enters the interior of the BamA barrel and exits at the periplasmic end of the lateral seam (Fig. 1b,d,e,h,i). The more membrane-distal POTRA 1–3 domains of BamA, together with the C-terminal portion of BamP, are poorly resolved in the structure and are modelled in all figures by placement of AlphaFold^20^ structures into the electron microscopy map (Fig. 1d and Supplementary Data 1).

**Fig. 2:**
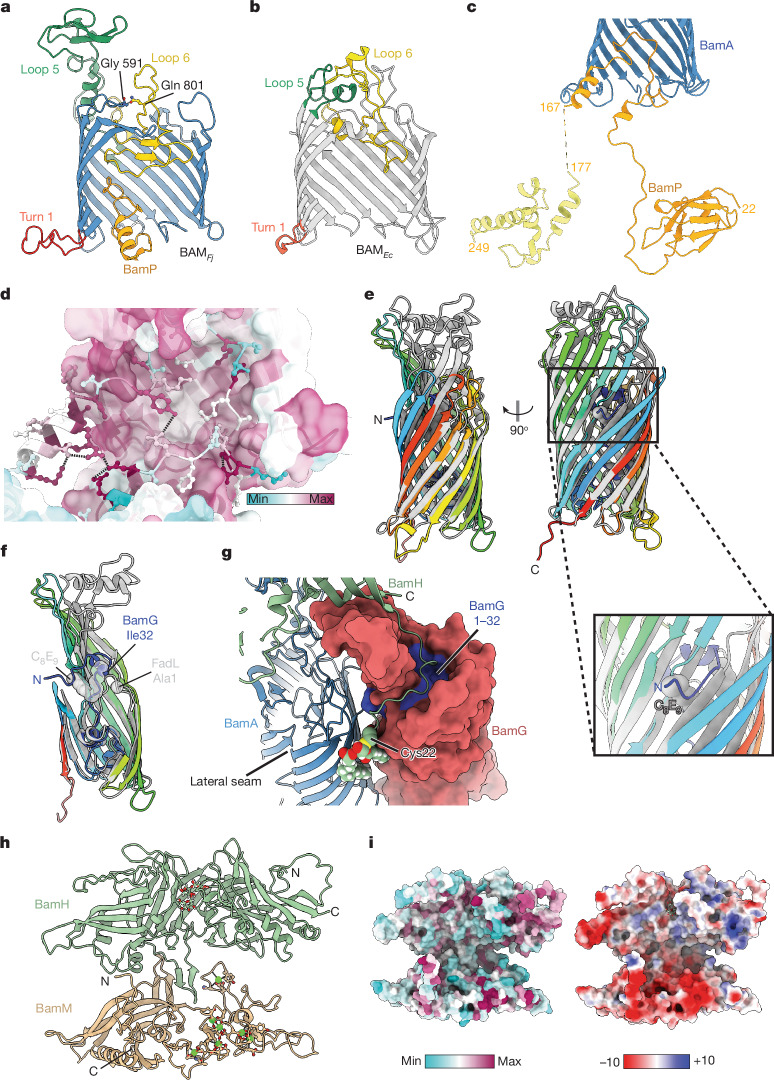
Structural features of the BAM_*Fj*_ subunits. **a**,**b**, Comparison of the *F. johnsoniae* (**a**) and *E. coli* (PDB: 8ADI) (**b**) BamA barrels. The strands closest to the viewer have been removed, revealing BamP within the *F. johnsoniae* barrel. The structure in **a** also highlights the hydrogen-bonding interaction between the side chain of Gln801 (substituted in the *bamH* suppressor mutant) and the main chain of Gly591 (both in ball and stick representation). **c**, Cartoon representation of BamP (orange). The C-terminal domain (pale yellow) is an AlphaFold model docked into the electron microscopy density. Portions of the BamA barrel (blue) are shown for orientation. **d**, Sequence conservation and intra-chain interactions of the inter-domain loop of BamP (cartoon with ball and stick side chains) within the BamA barrel (surface representation). Min, minimum; max, maximum. **e**,**f**, Superimposition of BamG (chainbows colouring) and *E. coli* FadL (grey; PDB: 3DWO). A proposed substrate-mimicking C_8_E_9_ detergent molecule in FadL is shown in grey spheres. In **f**, the front walls of the barrels, oriented as in **e**, left, are cut away and the N-terminal amino acid of FadL together with the equivalent sequence position residue in BamG are shown as spheres. **g**, View from outside the cell showing how the N-terminal region of BamH is bound by BamG. BamG is in surface representation with the N tail (residues 1–32) coloured blue. Partial structures of BamA and BamH are shown in cartoon representation with the N-terminal cysteine of BamH and its attached lipid groups shown as atomic spheres and coloured by atom. **h**, The BAM_*Fj*_ extracellular canopy viewed from BamA. Bound calcium ions and their coordinating side chains in BamM and glycosylation of BamH are shown in ball and stick representation. **i**, Surface conservation (left) and electrostatics (right, kcal (mol.e)^–1^ at 298 K) of the extracellular canopy in the same orientation as in **h**.

In structurally characterized BAM complexes, the lateral seam of BamA has been observed to be either open or closed^3,4^. In our BAM_*Fj*_ structure, BamA is in the closed state with the lateral seam sealed by a two-residue overlap between the N- and C-terminal strands (Fig. 1d,e). Multiple interstrand loops of the *F. johnsoniae* BamA barrel are extended relative to the canonical *E. coli* protein (Fig. 2a,b). First, 15 additional residues in periplasmic turn 1 (T1) form a short amphipathic structure along the periplasmic face of the OM that extends away from the BamA barrel. Second, 76 additional residues in extracellular loop 5 (L5) fold into a β-sheet domain that provides the binding platform for BamM. Finally, as in other BamA proteins, extracellular loop 6 (L6) enters the barrel lumen, where it contacts the barrel wall through a conserved VRGF/Y motif^8,21,22^ (the actual sequence being L^779^RGY^782^ in BamA_*Fj*_). However, in BamA_*Fj*_ an additional 16 residues form a β-strand-containing loop that extends across and fills the extracellular end of the pore, notably contacting the most deeply inserted piece of BamP. AlphaFold 3 modelling^23^ indicates that all three of these extended loop structures are highly conserved across Bacteroidota BamA proteins, although only the proteins from Flavobacteria include the BamM-binding domain at the tip of L5 (Supplementary Fig. 1).

The novel BamP subunit has a tripartite structure in which the N-terminal and C-terminal structured domains are joined by an extended linker (Fig. 2c). The N-terminal domain binds to POTRA 4 and POTRA 5 of BamA (Figs. 1d,e and 2c). The linker then extends up into the BamA barrel, which it penetrates as far as loop L6 (Fig. 2a) while making conserved contacts with the interior of the barrel (Fig. 2d), then exits the open periplasmic end of the lateral seam running back into the periplasm (Figs. 1d,e and 2a,c). BamP ends in a three-helix C-terminal domain that is sandwiched between, and thus links, BamA POTRA 1 and BamD (Fig. 1d,e,i).

BamG is a member of the FadL family of 14-stranded OMPs, which are characterized by a lateral opening in the transmembrane barrel and a long N-terminal tail that threads through the barrel pore to reach the extracellular side of the membrane^24^ (Fig. 2e,f). Canonical FadL proteins function as transporters for hydrophobic molecules. In these proteins, the lateral opening acts as a conduit to move hydrophobic substrate molecules between the protein interior and the membrane bilayer^25^. However, in BamG the N-terminal tail is extended and threads through the lateral opening with the N-terminal residue of the tail touching BamA (Fig. 2e–g). Many additional contacts between BamG and BamA are present and span the entire width of the bilayer. BamG also makes limited contact with BamD through the final three amino acids of its C-tail (Fig. 1d, right). BamG is O-glycosylated on the periplasmic portion of the N tail.

The extracellular portions of BamG anchor BamH to the BAM_*Fj*_ complex through extensive contacts. Strands 3 to 7 of the BamG barrel extend into the extracellular space to form the pillar onto which the proximal folded end of BamH docks (Fig. 2e–g). The lipidated N-terminal tail of BamH extends around the pillar in a deep groove on the BamG surface before exiting towards BamA (Fig. 2g), packing the three acyl chains between the BamA and BamG barrels (Figs. 1g and 2g).

BamH and BamM are both elongated two-domain proteins (Fig. 2h). The BamG-proximal end of BamH adopts a chondroitin sulfate-binding carbohydrate binding fold and is O-glycosylated facing BamA (Extended Data Fig. 2b,d). The N-terminal domain of BamM has a peptidyl-prolyl isomerase (PPI) fold (Extended Data Fig. 2c), whereas the C-terminal domain contains no well-defined secondary structural elements but is structured in part by the presence of seven metal ions (Fig. 2h and Extended Data Fig. 2e) assigned by their co-ordination chemistry as calcium ions. The phospholipid tail of BamM is not resolved. However, the N terminus of BamM is positioned to allow it to insert in the OM (Fig. 1b,d,e).

BamH and BamM pack along their long axes, where each interdigitates a β-hairpin into the other protein (Fig. 2h). The membrane-proximal side of the BamHM unit is likely to face substrate proteins and has a deep central valley (Fig. 2i). This surface is hydrophilic and highly acidic in the BamM portion, and shows little amino acid conservation (Fig. 2i), suggesting that it does not make highly specific interactions with substrates.

## Subunit conservation and essentiality

BamA, BamD, BamG and BamH are universally conserved across the Bacteroidota, whereas homologues of the full-length BamM protein are only found in the genus Flavobacterium and detectable homologues of BamP are restricted to the family Flavobacteriaceae (Extended Data Table 2 and Supplementary Table 1). This suggests that BamADGH constitute the core of the Bacteroidota BAM system. The BamG and BamH subunits are also conserved across six of the seven phyla that together with the Bacteroidota comprise the wider Fibrobacterota–Chlorobiota–Bacteroidota (FCB) superphylum (Extended Data Table 2), indicating that these phyla also possess a Bacteroidota-like BAM complex.

**Extended Data Fig. 3:**
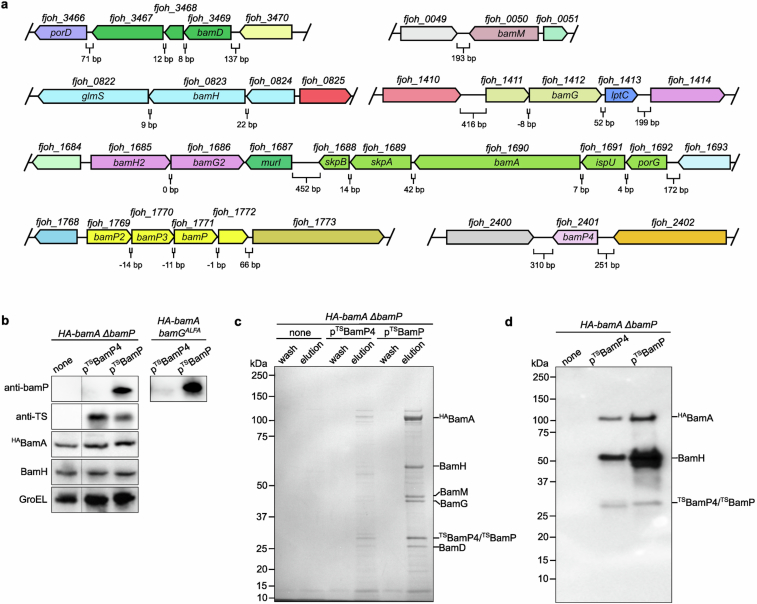
Genomic organisation of *F. johnsoniae bam* genes and biochemical evidence that BamP4 interacts with the BAM_*FJ*_ complex. **a**, Genomic organisation of *F. johnsoniae bam* genes. *porG*^66^ and *skpA*^19^ at the *bamA* locus code for components of the Type 9 Secretion system. BamH2 would be unlikely to interact with BamM as it lacks the protruding β-hairpin that BamH uses for this purpose. BamP homologues have related folded domains but markedly diverge in the interdomain loop. **b-d**, BamP interacts with the BAM_*FJ*_ complex. Strains in which the native BamA protein was fused to a HA tag (*HA-bamA* allele) were transformed with plasmids overproducing N-terminally Twin-Strep-tagged BamP (p^TS^BamP) or BamP4 (p^TS^BamP4). wt, wild type. Similar data were obtained for three biological repeats. **b**, Immunoblots of whole cells showing overproduction of BamP or BamP4 relative to native BamP levels. The cytoplasmic protein GroEL was used as a loading control. **c**,**d**, Affinity purification of Twin-Strep-tagged BamP and BamP4 complexes. **c**, Coomassie-stained SDS–PAGE gel of the wash and elution fractions. BAM subunits were assigned by comparison with (**d**) and Fig. 1a. **d**, Immunoblotting analysis of the elution fractions with anti-HA (to identify BamA), anti-BamH, and anti-Twin-Strep (to identify BamP and BamP4) antibodies.

*F. johnsoniae* possesses homologues of BamG (BamG2), BamH (BamH2) and three additional homologues of BamP (BamP2, BamP3 and PamP4) (Extended Data Fig. 3a and Extended Data Table 1). With the exception of BamP4, none of these homologues is expressed at an appreciable level in cells cultured on rich medium^26^. Pull-down experiments confirm that BamP4 binds to BAM_*Fj*_ in vivo (Extended Data Fig. 3b–d).

**Extended Data Fig. 4:**
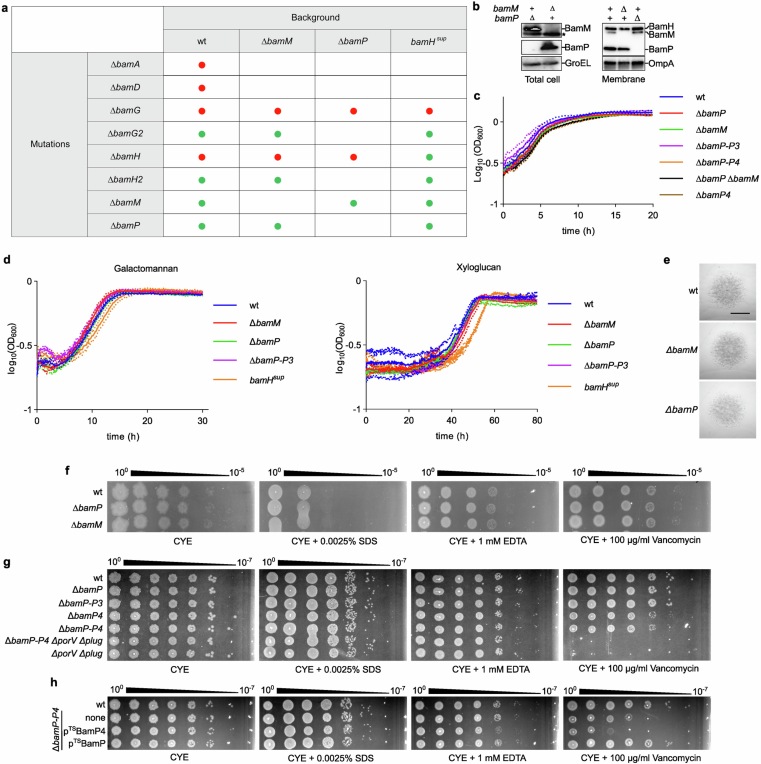
Phenotypic characterisation of strains with deletions in BAM_*FJ*_ subunits or BAM_*FJ*_ subunit homologues. **a**, Results of attempts to delete Bam_*FJ*_ subunits and their homologues in different genetic backgrounds. Mutations and their combinations that were viable are indicated by green dots, while those that could not be constructed are indicated by red dots and are assumed to disrupt essential cell functions. **b**, Immunoblots of whole cells and isolated membranes of strains containing in-frame deletions of *bamM* or *bamP*. The cytoplasmic protein GroEL and OM protein OmpA were used as loading controls. *, non-specific band. Similar results were obtained from 3 biological repeats. **c**, Growth curves on rich CYE medium. Shown are the means ± 1 SD from three biological repeats. **d**, Growth curves on minimal medium containing either galactomannan or xyloglucan as carbon source. Shown are the means ± 1 SD from three biological repeats. **e**, Spreading (gliding) morphology of colonies on agar. Scale bar, 5 mm. Similar data were obtained for three biological repeats. **f,g**, OM integrity assays. Cells were grown on CYE agar with the indicated additions. wt, wild type; Δ*bamP-P3*, strain deleted for BamP, BamP2, and BamP3; Δ*bamP-P4*, strain deleted for all BamP homologues (Δ*bamP-P3* Δ*bamP4*). The Δ*porV* Δ*plug* background permeabilizes the OM through opening the T9SS translocon channel^15^. Similar data were obtained for three biological repeats. **h**, BamP overproduction restores vancomycin resistance to a strain lacking all BamP homologues (strain Δ*bamP-P4*). Where indicated strains were transformed with plasmids overproducing N-terminally Twin-Strep-tagged BamP (p^TS^BamP) or BamP4 (p^TS^BamP4). wt, wild type. Similar data were obtained for three biological repeats.

We were unable to delete the genes for BamG or BamH (or in control experiments BamA and BamD) (Extended Data Fig. 4a), suggesting that these core BAM_*Fj*_ proteins are essential (confirmed below). By contrast, the genes encoding the accessory proteins BamM and BamP, as well as the various BAM_*Fj*_ subunit homologues could all be deleted (Extended Data Fig. 4a,b). Strains lacking either BamM or BamP, or all BamP homologues, exhibit no defects in growth on rich medium (Extended Data Fig. 4c) or on carbon sources (galactomannan or xyloglucan) that require SusCD systems to metabolize^27^ (Extended Data Fig. 4d), or in the T9SS-dependent process of gliding motility^28^ (Extended Data Fig. 4e). They also show no defect in the canonical BAM function of OMP insertion as assessed through standard chemical challenges for loss of OM integrity^5^ (Extended Data Fig. 4f,g), with the exception that loss of BamP4 results in a modest increase in sensitivity to vancomycin, which can be reversed by BamP overexpression, showing a functional equivalence between BamP4 and BamP (Extended Data Fig. 4h).

## Structural consequences of BamP removal

**Fig. 3:**
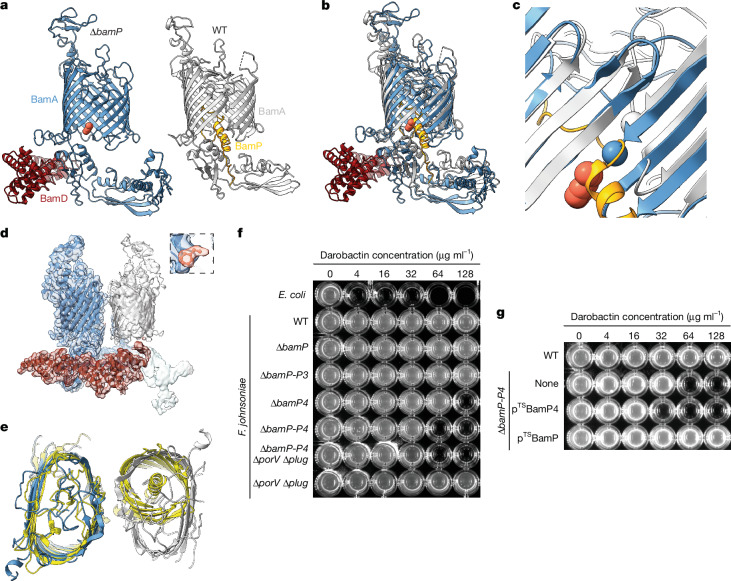
Structural and functional consequences of losing BamP. **a**, Comparison of the structure of the BamAD complex from a BamP-deleted (Δ*bamP*) background and a BamAP complex from the wild-type (WT) background. The proposed phenylalanine molecule is shown in orange space-filling representation. **b**, Overlay of the structures shown in **a** aligned on the N-terminal 100 residues of the BamA barrel. **c**, Detail from **b** showing the enlargement and register shift of the sheet between the BamA barrel N and C terminal strands upon BamP removal and the incompatible binding modes of BamP and the putative phenylalanine (orange space-filling representation). Spheres show the Cα atom of Gly897 in each model. **d**, Cryo-EM volume for the BamAD complex from a BamP-deleted background reveals a partially occupied second barrel (silver). Inset shows the putative phenylalanine density. **e**, Superposition of the complex in **d** with an *E. coli* BamA–EspP complex^29^ (PDB: 8BO2; yellow) aligning on the blue BamA_*Fj*_. The view is from the cell exterior but truncated in the periplasm for clarity. A second copy of BamA_*Fj*_ (silver) has been docked into the second barrel density in **d** and occupies the same position as the EspP substrate (yellow barrel, right). **f**, Removal of BamP homologues sensitizes *F. johnsoniae* to darobactin. The Δ*porV* Δ*plug* background permeabilizes the OM by opening the T9SS translocon channel^15^. **g**, BamP overexpression restores darobactin resistance to a strain lacking all BamP homologues. Strains contain plasmids overexpressing Twin-Strep-tagged BamP (p^TS^BamP) or BamP4 (p^TS^BamP4). **f**,**g**, Similar data were obtained for three biological repeats.

**Extended Data Fig. 5:**
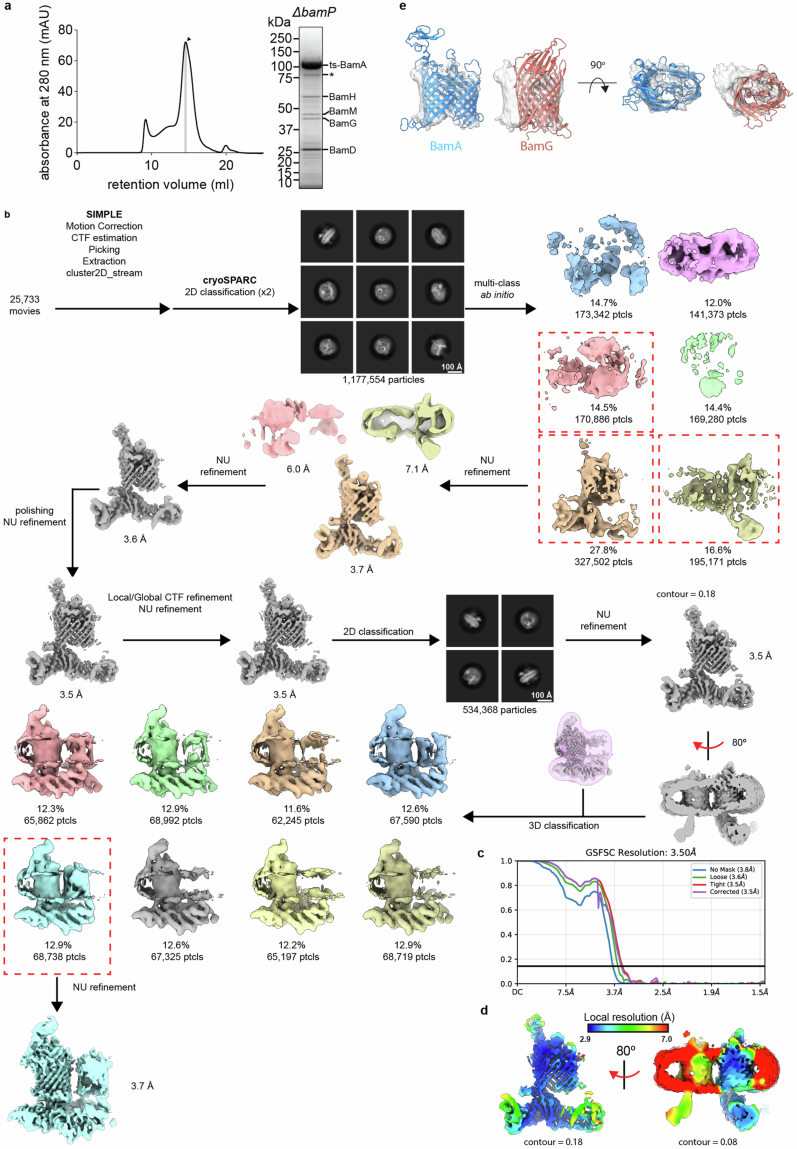
Workflow for the cryoEM analysis of the BamA complex isolated from a Δ*bamP* background. **a**, Size exclusion chromatography profile of BamA complexes purified from a BamP-deleted background together with a Coomassie-stained SDS–PAGE gel of the indicated peak fraction that was used for structure determination. BamA* indicates a proteolysis product of BamA. Similar results were obtained from 2 biological repeats. **b**, Image processing workflow for the ΔBamP complex. **c**, Gold-standard Fourier Shell Correlation (FSC) curves used for global resolution estimation. **d**, Local resolution estimate of the volume, displayed at two contour levels. **e**, Density for the unassigned second barrel taken from the focused 3.7 Å volume is shown with either a second copy of BamA or BamG docked. Two views are shown from the side (left) or the cell exterior (right). The shape and size of the volume is clearly more consistent with this being a second copy of BamA than BamG. Source data

**Extended Data Fig. 6:**
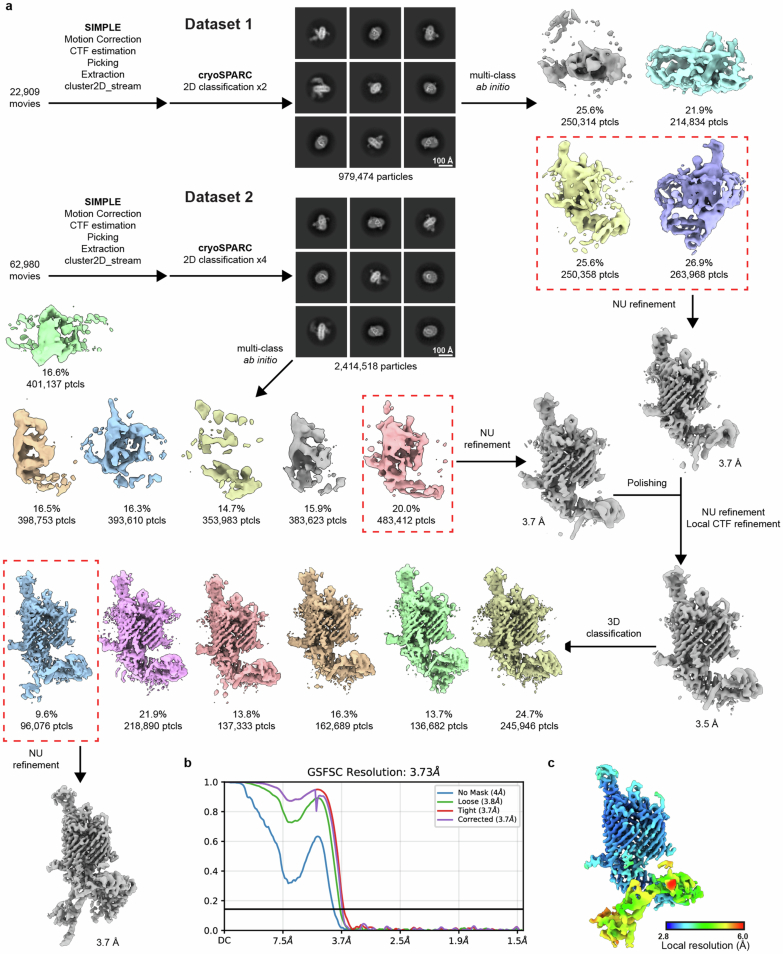
Workflow for the cryoEM analysis of the BamAP complex. The sample used was fraction B from Fig. 1a. **a**, Image processing workflow for the BamAP complexes. **b**, Gold-standard Fourier Shell Correlation (FSC) curves used for global resolution estimation. **c**, Local resolution estimate of the volume.

The central loop of BamP is bound at the lateral seam of BamA in a way that would sterically impede hybrid barrel formation with the substrate protein. This suggests that our BAM_*Fj*_ structure represents an inhibited or inactive state and that for catalysis to occur the BamP loop must be displaced. In an attempt to mimic the loop-displaced state we deleted the BamP subunit and structurally characterized the resulting BamA complex. Following grid preparation, only BamAD complexes were identified, even though the preparation also contained BamGHM proteins (Fig. 3a, Extended Data Fig. 5 and Extended Data Table 1). The loss of BamGHM does not in itself affect the conformation of the BamA barrel, because the barrel conformer does not change between the full BAM_*Fj*_ complex and a BamAP sub-complex that is present in the original BAM_*Fj*_ preparation (Figs. 1a and 3a,b Extended Data Fig. 6 and Extended Data Table 1).

In the absence of BamP, the lateral seam of the BamA barrel remains closed (Fig. 3a). However, the sheet between the barrel N and C termini shifts register and is enlarged through the formation of an additional hydrogen bond (Fig. 3b,c). The structure contains partial density for a second β-barrel next to the lateral seam (Fig. 3d) that is likely to represent a second copy of BamA (Fig. 3e and Extended Data Fig. 5e), as well as unconnected density at the periplasmic side of the BamA lateral seam that we model as a phenylalanine side chain of unknown origin (Fig. 3a–d).

The BamP-deleted state closely and uniquely resembles a complex between *E. coli* BAM and the substrate protein EspP (BAM–pair3-EspP in ref. ^29^; Protein Data Bank (PDB): 8BO2), which exhibits the same register shift between the first and last strands of BamA (Fig. 3e). Notably the position of the folded EspP substrate in the *E. coli* complex is very similar to the position of the second barrel in our BamP-deleted structure (Fig. 3e) suggesting a correlation between the register shift at the lateral seam and the presence of a barrel interacting at this position. The *E. coli* complex has been interpreted as representing the end state in OMP insertion^29^ and so our BamP-deleted complex may be an analogue of this state.

The presence of the BamP loop within the lateral seam of BAM_*Fj*_ would be expected to block binding of the BAM-specific antibiotic darobactin that also binds at this position^30^ (Fig. 1c), potentially explaining the insensitivity of Bacteroidota to this antibiotic^30,31^. Consistent with this hypothesis, we found that removing all four BamP homologues renders *F. johnsoniae* sensitive to darobactin (Fig. 3f). Deletion of BamP4 had the largest effect on darobactin sensitivity, but this was additive with removal of the other BamP homologues (Fig. 3f), and overexpression of BamP alone suppressed the effect of deleting all four BamP homologues (Fig. 3g and Extended Data Fig. 4h). Thus, all four BamP homologues are likely to interact with the same site on BamA to prevent darobactin binding, and the BamP proteins must be interacting with BamA during normal cell growth in order to provide their protective effect. The restricted phylogenetic distribution of BamP proteins within the Bacteroidota (Extended Data Table 2) suggests that other organisms within the phylum either have other mechanisms for darobactin resistance or possess unrecognized, mechanistically analogous proteins.

## Depletion of essential BAM_*Fj*_ subunits

**Fig. 4:**
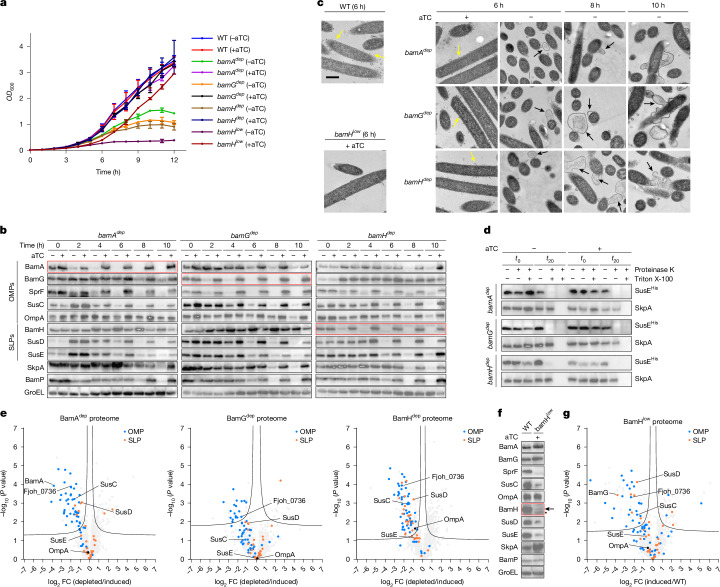
Depletion analysis of the essential BAM_*Fj*_ subunits. Strains are the wild-type and depletion strains for BamA (*bamA*^*dep*^), BamG (*bamG*^*dep*^) and BamH with either a strong (*bamH*^*dep*^) or weak (*bamH*^*low*^) inducible promoter. SkpA is a periplasmic protein to control for OM integrity; GroEL is a loading control. **a**–**d**, Strains were cultured in rich (Casitone yeast extract, CYE) medium. The aTC inducer of the target gene was removed at 0 h where indicated (−aTC) to initiate subunit depletion. Samples in **b**–**d** were taken at the indicated time points in **a**. **a**, Growth curves. Data are mean ± s.d. **b**, Immunoblots of whole-cell lysates. **c**, Representative transmission electron microscopy images showing OM defects in the depletion strains. Yellow arrows highlight budding OM vesicles; black arrows highlight OM blebbing and rupture. Scale bar, 500 nm. **d**, Depletion of BAM_*Fj*_ subunits for 6 h does not change the surface exposure of the SLP SusE as assessed by proteinase K accessibility. Triton X-100 permeabilizes the OM. Reactions were stopped immediately (*t*_0_) or after 20 min (*t*_20_) and analysed by immunoblotting. **e**, Comparative whole-membrane proteome analysis of depleted (−aTC) versus induced (+aTC) strains collected at the 6 h time point in **a**. Data points for OMPs and SLPs are coloured as indicated and the most highly expressed OM proteins are labelled. A significance threshold is drawn according to a two-tailed *t*-test with a false discovery rate (FDR) of 0.1 and a variance correction constant *S*_0_ of 0.1. Data are averaged over three biological repeats. FC, fold change. **f**,**g**, Analysis of chronic BamH depletion in an induced strain (*bamH*^*low*^ + aTC) in which a weak promoter results in the incomplete restoration of wild-type BamH levels. **f**, Whole-cell immunoblots. Arrow indicates BamH; asterisk indicates a non-specific band. **g**, As **e** but comparing chronic BamH depletion (*bamH*^*low*^ strain + aTC) relative to the wild type. **a**–**d**,**f**, Similar data were obtained for three biological repeats. Source data

**Extended Data Fig. 7:**
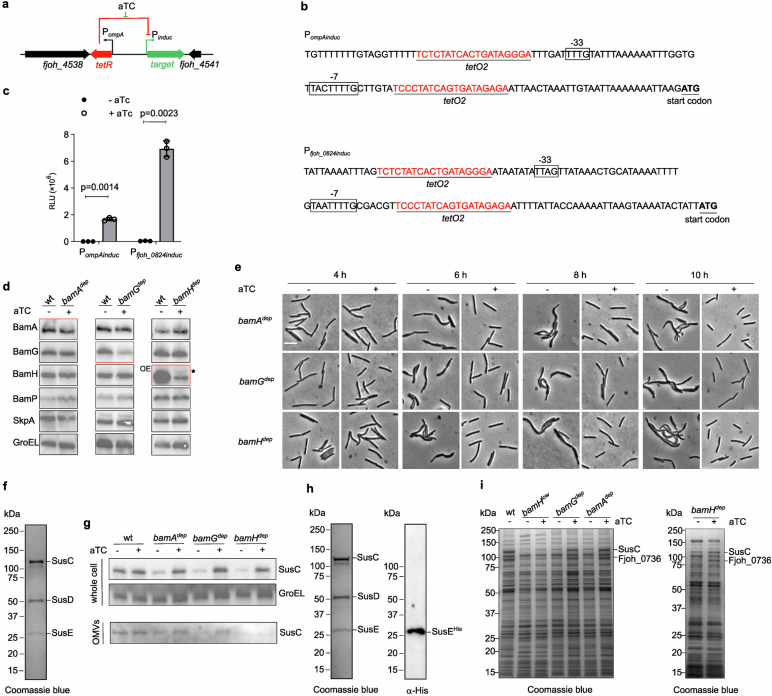
Depletion analysis of the essential *F. johnsoniae* BAM complex subunits. **a**, Design of an anhydrotetracycline (aTC)-inducible system for the depletion of essential target genes in *F. johnsoniae*. The TetR repressor is constitutively expressed under the control of the *F. johnsoniae ompA* promoter (P_*ompA*_) and the target gene is regulated by a designed TetR-repressed promoter (P_*induc*_). In the presence of the inducer aTC repression of the target gene by TetR will be released. The genetic system is integrated into the *F. johnsoniae* chromosome at a neutral locus. **b**, Sequences of the designed inducible P_*ompA-induc*_ and P_*fjoh_0824-induc*_ promoters. *tetO2* arrays are placed upstream and downstream of the conserved −33 and −7 RNA polymerase binding sites (boxed) of the selected promoters. **c**, Tight regulation of protein expression by the designed inducible systems. Strains expressing NanoLuc under the control of either the P_*ompA*indc_ promoter (XLFJ_1095) or the P_*fjoh_0824*induc_ promoter (XLFJ_1100) were grown to mid-exponential phase (OD_600_ = 0.6) in the presence or absence of aTC and the luminescence signal measured. Error bars represent the mean ± 1 SD from three biological repeats. *P* values were determined with a two-sided paired Student’s t-test. RLU, relative luminescence units. **d**, Comparison of the expression levels of Bam subunits in the wild type strain (wt, XLFJ_1078) and corresponding depletion strains grown in the presence of the inducer aTc (*bamA*^*dep*^, XLFJ_1129; *bamG*^*dep*^, XLFJ_1115; *bamH*^*dep*^, XLFJ_1140). Whole cell immunoblotting of cells grown to mid-exponential phase (OD_600_ = 0.6). The blots for the depleted subunit are boxed in red. The BamH blot for the BamH depletion comparison is overexposed (OE) relative to the other BamH blots in order to detect the low levels of BamH in the depletion strain. BamA and BamG are detected via epitope tags. *, non-specific band. Similar results were obtained for 3 biological repeats. **e**, Phase contrast images of cells sampled at the indicated time points in the BAM_*FJ*_ subunit depletion experiments shown in Fig. 4a. Scale bar, 10 µm. Similar results were obtained for 3 biological repeats. **f**, The major *F. johnsoniae* SUS complex is composed of SusC (Fjoh_0403), SusD (Fjoh_0404), and SusE (Fjoh_0405). The native SUS complex was purified via a Twin-Strep tag on the N-terminus of SusC followed by size exclusion chromatography and analysed on a Coomassie-stained SDS–PAGE gel. Proteins were identified by peptide mass fingerprinting. Similar data were obtained for two biological repeats. **g**, Outer membrane vesicle (OMV) production does not increase upon BAM depletion. Immunoblotting of the OM protein SusC in whole cells or the OMV fraction at the 6 h time point in Fig. 4a. GroEL serves as loading control. Similar results were obtained for 3 biological repeats. **h**, An exogenously-expressed His-tagged variant of SusE (SusE^His^) is incorporated into the native SusCDE complex. SusC-containing complexes were purified as described in **f** from cells expressing SusE^His^ from a plasmid. The purified material was separated by SDS-PAGE and characterized by Coomassie-staining (Left) and anti-His tag immunoblotting (Right). Similar data were obtained for two biological repeats. **i**, Exemplar Coomassie-stained SDS-PAGE gel of the whole membrane samples used for the comparative proteome analysis (Fig. 4e,g) of induced/non-induced (i.e. undepleted/depleted) BAM subunit depletion strains harvested at the 6 h time point in Fig. 4a. Proteins present in the two obviously depleting bands were assigned by peptide mass fingerprinting. The data are representative of the three repeats used for the proteomics analysis. Source data

To gain insight into the roles of the essential BamG and BamH proteins we developed a genetic system to enable gene depletion in *F. johnsoniae*. In this system, a duplicate copy of the gene of interest is expressed ectopically on the chromosome under the control of a TetR-repressible promoter (Extended Data Fig. 7a–d). Provided expression of this second copy of the gene is maintained by the inclusion of the inducer anhydrotetracyline (aTC) in the growth medium, the native copy of the gene can be deleted. Omission of aTC in the resultant strain prevents further synthesis of the target protein leading to its depletion as the cells grow and divide. Using this strategy, we confirmed that BamG, BamH and BamA are essential for growth under standard laboratory conditions (Fig. 4a). In all three cases, full depletion of the target protein is apparent by 6 h after removal of the inducer (Fig. 4b), at which point cell growth slows (Fig. 4a). Within a further 2 h, the cells become misshapen (Extended Data Fig. 7e) and start to lose periplasmic contents (Fig. 4b, SkpA lanes). More detailed analysis of the depleted cells by transmission electron microscopy shows that all three depletion strains exhibit a similar perturbed morphology in which the OM no longer buds OM vesicles^32^, but is deformed by massive blebbing, while the inner membrane remains intact (Fig. 4c). Thus, depletion of any of the three essential BAM_*Fj*_ subunits leads to gross defects in OM biogenesis, similar to those reported in *E. coli* following BamA depletion^33^.

We used immunoblotting to assess the effects of depletion of BAM subunits on the cellular levels of the remaining BAM_*Fj*_ components and of representative OMPs and SLPs (Fig. 4b). The analysed proteins include the two most abundant *F. johnsoniae* OM components^26,34,35^, OmpA (Fjoh_0697), an 8-strand OMP that anchors the OM to the cell wall, and a SUS complex of unknown function that we show here to be composed of a 22-strand SusC OMP (Fjoh_0403) together with its SusD SLP partner (Fjoh_0404) and a structurally unrelated SLP SusE (Fjoh_0405) (Extended Data Fig. 7f). We also assessed the levels of SprF, a 14-strand OMP involved in gliding motility^36^. The effects of depleting all three BAM_*Fj*_ subunits were broadly similar. Levels of OMPs decreased after depletion of the target subunit, although at differing rates. OmpA is notably slow to deplete, and it is possible that other BamA homologues present in *F. johnsoniae* may also be able to insert this simple OMP, as has recently been demonstrated for the *E. coli* translocation assembly module (TAM) complex^37^. The levels of the SLPs (SusD and SusE) also decreased, with the exception of BamH, which instead increased.

Because *F. johnsoniae* releases OM vesicles (Fig. 2c and ref. ^32^), we investigated whether the reduced OM protein levels in the depletion strains were a consequence of OM loss through vesicle shedding. However, we detected no increase in OM protein in the vesicle fraction of the culture supernatant (Extended Data Fig. 7g). Thus, as in *E. coli*^38^, the OM is not lost through vesicle production when BAM is depleted. The observed reduction in OMP levels therefore reflects defects in their biogenesis.

Analysis of the surface exposure of an overexpressed tagged version of the SLP SusE provides no evidence that SLPs are accumulating inside the depletion strains, and thus no evidence that their export is blocked (Fig. 4d and Extended Data Fig. 7h).

**Extended Data Fig. 8:**
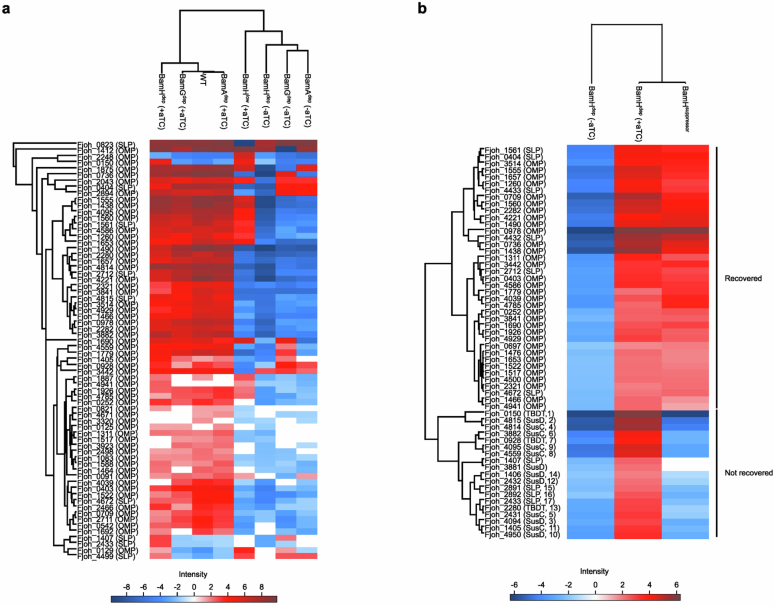
OM proteomics data comparisons. Heat maps of the indicated strains after hierarchical protein clustering of the entire datasets and post hoc ANOVA testing. Only proteins classified as OMPs or SLPs are displayed. Colours indicate HSD (honestly significant difference) values according to the intensity panel. **a**, Comparison of the datasets used in Fig. 4e and g. **b**, Comparison of the *bamH*^*sup*^ mutant dataset with the induced and non-induced *bamH*^*dep*^ datasets. The non-recovered proteins are numbered as in Fig. 5d and assigned to SusC, SusD, other SUS SLP, or TonB-dependent transporter (TBDT) protein families. TBDTs are 22-strand OMPs that are related to the SusC family. Source data

We extended our analysis of the effects of the BAM subunit depletions to the whole OM proteome (Fig. 4e and Extended Data Fig. 7i). We analysed membranes collected 6 h after removal of the inducer, at which point depletion of the target subunit is complete but the other BAM_*Fj*_ subunits are still present and the OM is still intact (Fig. 4a–c). The overall pattern of OM proteome changes in all three depletions is similar, with marked decreases in the levels of many OMPs and some SLPs (Fig. 4e and Extended Data Fig. 8a). Thus, removal of the essential BAM_*Fj*_ subunits has the general effect of reducing the levels of OM proteins.

As an alternative to fully depleting the BAM_*Fj*_ subunits, we also investigated the effects of chronically reducing the steady-state concentration of BamH to a level at which there is a marked effect on cell growth (Fig. 4a,f). Cells of this strain had less severe defects in OM morphology than after full BamH depletion, although the budding of OM vesicles seen in the parental strain was almost fully suppressed (Fig. 4c). The differences in the steady-state OM proteome in this strain relative to that in wild-type cells followed the same trends as the proteome changes seen in the total depletion experiments in showing a general reduction in OMPs and SLPs (Fig. 4f,g and Extended Data Figs. 7i and 8a).

In summary, the loss of either BamG or BamH results in changes in the OM proteome and cellular morphology that closely match those associated with the total loss of BAM function that occurs when BamA is removed. Thus, BamG and BamH are both essential for the core BAM_*Fj*_ function of OMP insertion.

## Isolation of a *bamH* suppressor mutant

**Extended Data Fig. 9:**
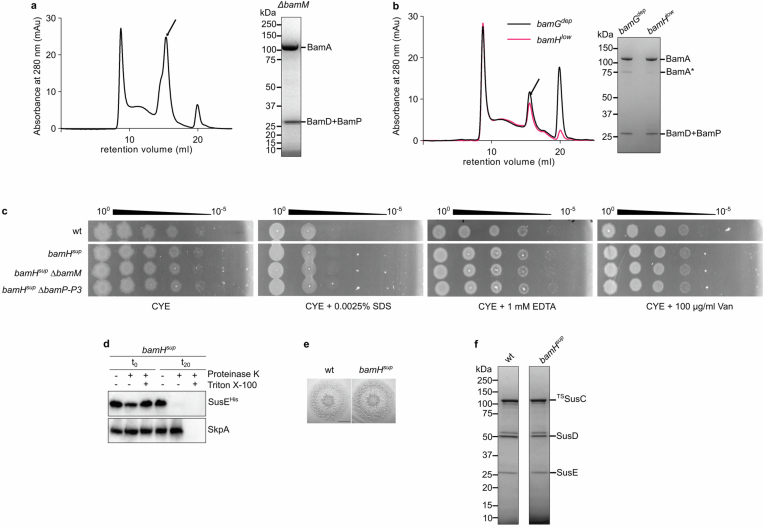
Isolation of BamA complexes from subunit depleted backgrounds and phenotypic characterisation of the *bamH*^*sup*^ strain. **a**,**b**, Isolation of BamA complexes either (**a**) in the absence of BamM or (**b**) after 6 h of depletion of the essential BamG or BamH subunits. Size exclusion chromatography profile of Twin-Strep-tagged BamA complexes purified by Streptactin affinity chromatography (Left) and a Coomassie-stained SDS–PAGE gel of the indicated peak fractions (Right). BamA* indicates a proteolysis product of BamA. The identities of the BamA* and BamD + BamP bands were assigned by peptide fingerprinting. Similar results were obtained from 2 biological repeats. **c-f**, Characterization of the recreated *bamH*^*sup*^ mutant (*bamA*^*Q801K*^ Δ*bamH* Δ*bamH2*). wt, wild type. Similar results were obtained for three biological repeats. **c**, OM integrity assays. Cells were grown on CYE agar with the indicated additions. **d**, Surface exposure of the SLP SusE. Strains expressing a protease-sensitive His-tagged variant of SusE (SusE^His^) were treated as indicated with Proteinase K and the detergent Triton X-100 (to permeabilise the OM). Reactions were stopped immediately (t_0_) or after 20 min (t_20_) and analysed by immunoblotting with His tag antibodies. The periplasmic protein SkpA serves as an OM integrity control. **e**, Spreading (gliding) morphology of colonies on agar. Scale bar, 5 mm. **f**, Purification of the native SusCDE complex via a Twin-Strep tag on the N-terminus of SusC followed by size exclusion chromatography. Analysed on a Coomassie-stained SDS–PAGE gel. Source data

The requirement for BamG and BamH in BAM_*Fj*_ function could reflect a direct involvement of these subunits in the general OMP biogenesis function of the BAM complex. However, the same phenotype could also arise indirectly if BamG and BamH have a specialized role in the maturation of a subset of BAM_*Fj*_ clients such that in their absence these clients accumulate on BamA and interfere with its ability to carry out general OMP biogenesis. Although no additional proteins corresponding to trapped substrates were co-purified with BamA complexes isolated from strains depleted for BamG or BamH (Extended Data Fig. 9a,b), the hypothesis that BamG and BamH have client-specific roles in BAM_*Fj*_ function suggests that it might be possible to identify suppressor mutations that relieve the secondary effects on general BAM function of BamG or BamH removal.

**Fig. 5:**
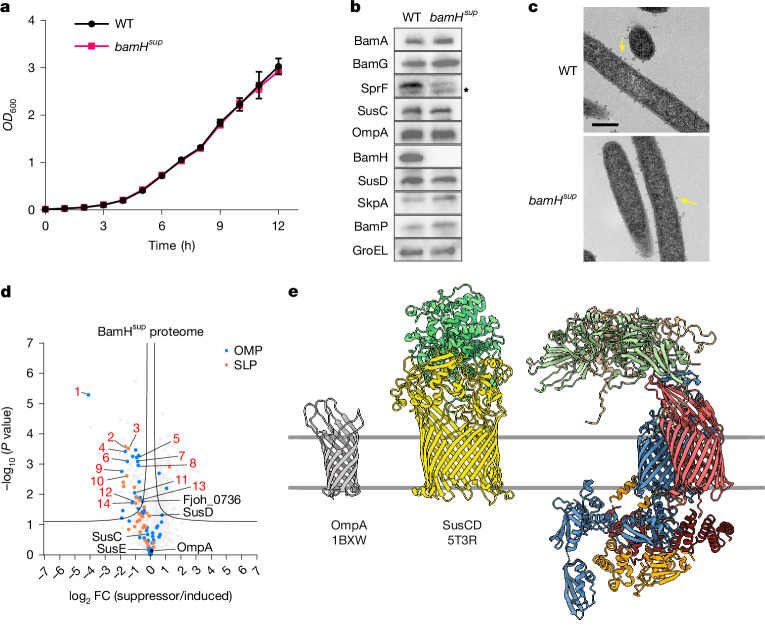
Characterization of a *bamH* suppressor mutant. Comparative characterization of the recreated *bamH*^*sup*^ mutant (*bamA*^*Q801K*^ Δ*bamH* Δ*bamH2*) and wild-type strains. **a**, Growth on rich (CYE) medium in the absence of aTc. Data are mean ± s.d. **b**, Whole-cell immunoblots. SkpA is a periplasmic protein to control for OM integrity. GroEL is a cytoplasmic protein as loading control. BamA and BamG are detected via epitope tags. Asterisk indicates non-specific bands. **c**, Representative transmission electron microscopy images of the wild type and *bamH*^*sup*^ mutant. Yellow arrows highlight budding OM vesicles. Scale bar, 500 nm. **d**, Comparative whole-membrane proteome analysis of the *bamH*^*sup*^ strain relative to a BamH-induced strain (*bamH*^*dep*^ + aTC). Data points for OMPs and SLPs are coloured as indicated and the most highly expressed OM proteins are labelled. Proteins that show poor recovery in the *bamH*^*sup*^ strain in a post hoc ANOVA with BamH-induced and depleted strains are numbered as in Extended Data Fig. 8b. A significance threshold is drawn according to a two-tailed *t*-test with a FDR of 0.1 and a *S*_0_ of 0.1. Data are averaged over three biological repeats. **a**–**c**, Similar data were obtained for three biological repeats. Cells were analysed (**b**,**c**) and membranes prepared (**d**) at the 6 h time point in **a**. **e**, Size comparison between BAM_*Fj*_ and a typical SusCD complex and OmpA. SusCD and OmpA are illustrated using homologous proteins of known structure from other organisms (labelled with their PDB accession numbers). Source data

We were able to select a spontaneous mutant of the BamH depletion strain that allowed growth in the absence of the inducer aTC. Genome sequencing identified a Q801K substitution in BamA as most probably responsible for the suppressor phenotype. Re-creation of the BamA Q801K substitution in a clean background permitted deletion of both *bamH* and its orthologue *bamH2*, confirming that this single amino acid substitution was responsible for the *bamH* suppressor phenotype and that it did not operate through upregulating *bamH2* expression. The resultant *bamA*^*Q801K*^ Δ*bamH* Δ*bamH2* strain (hereafter *bamH*^*sup*^) grew as rapidly as the wild-type strain on rich medium (Fig. 5a), even though BamH was absent (Fig. 5b). Thus, although *bamH* behaves as an essential component of BAM_*Fj*_ in the native context, it is dispensable in an experimentally modified genetic background. This has parallels to the way that *E. coli* BamD can be deleted in a *bamA* suppressor background^39^. Of note, the *bamA*^*Q801K*^ mutation did not allow deletion of *bamG*, indicating that BamH and BamG have non-identical functions (Extended Data Fig. 4a).

The *bamH*^*sup*^ strain had normal cellular morphology (Fig. 5c) and no defect in OM integrity, SLP export or gliding motility (Extended Data Fig. 9c–e). The SusC, SusD and SusE proteins were restored to wild-type levels (Fig. 5b) and the cell was able to assemble these proteins into SusCDE complexes (Extended Data Fig. 9f). Thus, the most abundant *F. johnsoniae* SUS system does not depend on BamH for its biogenesis.

Analysis of the OM proteome of the suppressor strain showed strong restoration of the levels of many OMPs and SLPs relative to BamH-depleted conditions (Fig. 5d and Extended Data Fig. 8b). However, the levels of other OM proteins recovered poorly, suggesting that these proteins were particularly sensitive to the loss of BamH. These sensitive proteins were almost all SusCD pairs and their SLP partners (Extended Data Fig. 8b). Thus, BamH may be particularly important in the biogenesis of a subset of SUS systems.

## Discussion

Our phylogenetic and experimental analyses indicate that BamADGH constitute the essential core of the Bacteroidota BAM complex with which a species-variable complement of accessory subunits associate. This pattern is consistent with a contemporaneous characterization of BAM complexes from *Bacteroides thetaiotaomicron* and *P. gingivalis*^40^, which conserve only BamADGH from the BAM_*Fj*_ complex but have three distinct and non-essential SLP subunits (though two of these have PPI folds resembling one of the BamM_*FJ*_ domains). Note that the subunit nomenclature in that study matches that used here. Variation in accessory subunit composition has previously been observed between the BAM complexes of Proteobacteria^41,42^.

The Bacteroidota BAM complex has previously been proposed to be involved in SLP export^13,16^. Consistent with this suggestion, we find that blocking core BAM_*Fj*_ function through depletion of BamA reduces SLP levels (Fig. 4a–c). However, assigning causality is complex because any OM protein involved in SLP export will indirectly depend on BAM_*Fj*_ for their own biogenesis. Furthermore, our biochemical analysis provides no evidence that loss of BAM_*Fj*_ leads to the accumulation of non-exported SLPs inside the cell (Fig. 4d).

The BAM_*Fj*_ canopy provides a protected extracellular cavity above the position in the membrane where client OMPs assemble on BamA, suggesting that it functions as an extracellular folding vestibule. A possible precedent for a BAM-like machine providing *trans*-side folding assistance comes from the mitochondrial SAM complex, which contains subunits that contact client OMPs from the cytoplasmic (external) side of the membrane^43^. The BAM_*Fj*_ canopy might protect folding intermediates on the BAM complex from proteolysis by sterically blocking the access of proteases in the extracellular environment. Similarly, the presence of the canopy should sterically exclude lipopolysaccharide molecules (which have large head groups and form rigid arrays in the OM^1,44^) from the region of the membrane next to the lateral seam. This would provide a patch of phospholipid bilayer in the OM for client OMPs to fold into.

The novel BamG, BamH and BamM subunits of BAM_*Fj*_ are likely to expand the range of OMPs inserted relative to the canonical BAM_*Ec*_ complex and therefore act on specific structural classes of proteins that are found only in the Bacteroidota. In addition, their cell surface location implies that the novel subunits act on the extracellular portions of BAM substrates. Given these expectations, it is likely that these components are involved in one or more of the following processes: biogenesis of OMPs with large extracellular regions; assisting BamA to transport and fold SLPs (but see comments above); or allowing the assembly of the abundant SusCD family complexes that characterize the Bacteroidota OM. In this context, it may be important that the only FCB phylum that lacks BamG and BamH proteins (the Chlorobiota) also lacks SusCD systems and the FCB-specific T9SS translocon channel SprA, which has more than 150 kDa of polypeptide on the extracellular side of the membrane^15^ (Extended Data Table 2). Thus, there is a correlation between having a BAM_*Fj*_-like BAM complex and being able to build the complex OMPs that characterize the FCB superphylum. Our observation that a subset of SusCD proteins are only minimally recovered by a *bamH* suppressor mutation (Fig. 5d) supports the idea that at least BamH is involved in the assembly of some SusCD systems.

In Fig. 5e, we compare the proportions of BAM_*Fj*_ with those of the SusCD unit that it may assemble as well as the more classical *E. coli* OMP substrate OmpA that does not have an extensive extracellular domain. Although SusC could be accommodated under the BAM_*Fj*_ canopy, the full SusCD complex cannot do so without the canopy being raised. This appears unlikely owing to the tethering of the canopy to BamA and BamG at one end, and to the membrane by the lipid anchor of BamM at the other. Thus, SusC is likely to fold on BAM_*Fj*_ and be at least partially released before forming a complex with its SusD partner.

We were able to select a suppressor mutation in *bamA* that compensates for the loss of the BamH subunit. This single amino acid substitution in BamA is sufficient to restore OMP biogenesis and OM morphology (Fig. 5b,c), showing that general OMP insertion in *F. johnsoniae* does not physically require the presence of BamH. It is unlikely that the suppressing amino acid substitution in BamA functions by replicating the role of BamH, as it is difficult to see how alterations in BamA could create a similar structural environment to the BamH-containing extracellular canopy. Instead, it is most plausible that the suppressor substitution acts by compensating for the toxic consequences of loss of BamH function. Since removal of BamH closely phenocopies the loss of BamA (Fig. 4b,c,e–g), the most probable suppression scenario is that loss of BamH blocks BamA function through the accumulation of stalled BamH-requiring substrates and that this blockage is relieved by a structural change in BamA that corrects the problem, for example, by accelerating substrate release. Gln801, the BamA residue that is substituted in the *bamH* suppressor, is located in extracellular loop L6, which lies over the extracellular end of the BamA pore (Fig. 2a). Gln801 is hydrogen-bonded through its side-chain oxygen atom to the main chain amine of Gly591 in adjacent loop L5 (Fig. 2a), so it is likely that its substitution disrupts the packing of the BamA extracellular cap. We speculate that this may marginally destabilize BAM–substrate interactions, allowing the release of misfolded substrates.

Although analysis of the *bamH* suppressor allowed us to identify certain SusCD proteins that are heavily dependent on BamH for their biogenesis, many other SusCD systems, including the most abundant SusCDE complex, were well-restored in the same background (Fig. 5b,d Extended Data Fig. 9d). We interpret this as indicating that most BamH clients are able to fold without BamH during the vast majority of BAM_*Fj*_ turnovers and that BamH is only required to correct a small proportion of insertion events that go wrong. In this model, BamH has a role in quality control that prevents infrequent errors in folding blocking the BAM_*Fj*_ complex. Alternatively, BamH may have a more critical role in the biogenesis of these proteins under specific conditions, such as stress or under conditions that are not readily replicated in the laboratory.

## Methods

### Bacterial strains and growth conditions

All strains and plasmids used in this work are listed in Supplementary Tables 2 and 3. *F. johnsoniae* was routinely cultured aerobically in Casitone yeast extract (CYE) medium^45^ at 30 °C with shaking. For some physiological studies the cells were cultured in PY2 medium^46^ as indicated below. For experiments testing growth on complex sugars cells were cultured in a 96-well plate in a CLARIOstarPlus plate reader using modified minimal A medium^27^ and containing 0.25% (w/v) of either carob galactomannan (Megazyme, 11078-30-1) or tamarind xyloglucan (Megazyme, 37294-28-3) as the sole carbon source. *E. coli* strains were routinely grown aerobically in LB medium at 37 °C with shaking, or on LB agar plates. Where required, 100 µg ml^−1^ erythromycin was used in the growth medium for *F. Johnsoniae*. 100 µg ml^−1^ ampicillin or 50 µg ml^−1^ kanamycin were used in the growth medium for *E. coli*. aTC (CAY10009542-50 mg, Cambridge Bioscience) was used as a final concentration of 0.2 µg ml^−1^ (liquid culture) and 2 µg ml^−1^ (agar plates).

### Genetic constructs

Plasmids were constructed by Gibson cloning^47^ using the primers and target DNA in Supplementary Table 4. Suicide and expression plasmids were introduced into the appropriate *F. johnsoniae* background strain by triparental mating as previously described^46^. Chromosomal modifications were introduced using the suicide vector pYT313 harbouring the counter-selectable *sacB* gene as previously described^48^. All plasmid constructs and chromosomal modifications were confirmed by sequencing.

### Construction of a tightly regulated gene expression system for *F. johnsoniae*

The aTC-inducible systems for the depletion of essential Bam_*FJ*_ components (Extended Data Fig. 7a) were based on the native *F. johnsoniae* *ompA* and *fjoh_0824* promoters and contain the 100 bp upstream of *ompA* or *fjoh_0824*. Guided by the observations of Lim et al.^49^, a* tetO2* site (TetR binding site) was inserted upstream of the conserved −33 motif in these promoters and another *tetO2* site downstream of the conserved −7 motif generating the synthetic promoters *P*_*ompAinduc*_ and *P*_*fjoh_0824induc*_ (Extended Data Fig. 7b). The constructs also contain *tetR* under the control of an additional copy of the constitutive *F. johnsoniae ompA* promoter. The final inducible systems containing the gene to be induced were integrated into the chromosome at an assumed phenotypically neutral site^26,36^ by replacing *fjoh_4538* to *fjoh_4540*.

The designed inducible systems were validated using strains in which a NanoLuc reporter gene^50^ was placed under the control of the chromosomally integrated aTC-inducible systems (Extended Data Fig. 7c). Overnight cultures of these strains were diluted 1:100 into fresh CYE medium in the absence or presence of 0.2 µg ml^−1^ aTC and cultured for 6 h to mid-exponential phase (OD_600_ ~ 0.6). Cells were collected and resuspended in PY2 medium to OD_600_ = 0.6. A volume of 50 µl of cell resuspension was mixed with 50 µl of reaction solution (48 µl PY2 medium supplemented with 2 µl of furimazine (Promega)) in a 96-well plate and the luminescence signal measured in a CLARIOstar^Plus^ plate reader.

Strains to enable the depletion of the essential BAM_*Fj*_ subunits were constructed by introducing a copy of the target gene under the control of the designed inducible system into the chromosome at the phenotypically neutral site. The native copy of the target gene was then deleted in the presence of aTC to allow expression of the introduced copy of the gene.

### Purification of BAM_*Fj*_ and SusCDE complexes

To purify complexes containing Twin-Strep tagged BamA, the relevant strain was cultured for 22 h in CYE medium using 1 l culture volume in 2.5 l flasks. A total culture volume of 12 l was used for sample preparations for structure determination, and 4 l of culture was used for analytical purifications of BAM_*Fj*_ variants. Cells were collected by centrifugation at 12,000*g* for 30 min and stored at −20 °C until further use. All purification steps were carried out at 4 °C. Cell pellets were resuspended in buffer W (100 mM Tris-HCl pH 8.0, 150 mM NaCl, 1 mM EDTA) containing 30 μg ml^−1^ DNase I, 400 μg ml^−1^ lysozyme and 1 mM phenylmethylsulfonyl fluoride (PMSF) at a ratio of 5 ml of buffer to 1 g of cell pellet. Cells were incubated on ice for 30 min with constant stirring before being lysed by two passages through a TS series 1.1 kW cell disruptor (Constant Systems) at 30,000 PSI. Unbroken cells were removed by centrifugation at 20,000*g* for 20 min. The supernatant was recovered and total membranes were collected by centrifugation at 230,000*g* for 75 min. Membranes were resuspended in buffer W to a protein concentration of 6.5 mg ml^−1^ and solubilized by incubation with 1% (w/v) lauryl maltose neopentyl glycol (LMNG, Anatrace) for 2 h. Insoluble material was removed by centrifugation at 230,000*g* for 75 min. Endogenous biotin-containing proteins were masked by addition of 1 ml BioLock solution (IBA Lifesciences) per 100 ml of supernatant and incubation for 20 min with constant stirring. The solution was then circulated through a Strep-TactinXT 4Flow High Capacity column (IBA Lifesciences) overnight. The column was washed with 10 column volumes of buffer W containing 0.01% LMNG (buffer WD) and bound proteins were eluted with 6 column volumes Strep-TactinXT BXT buffer (IBA Lifesciences) containing 0.01% LMNG. The eluate was concentrated to 500 μl using a 100-kDa molecular weight cut-off (MWCO) Amicon ultra-15 centrifugal filter unit (Merck) and then injected onto a Superose 6 Increase 10/300 GL column (Cytiva) previously equilibrated in buffer WD. Peak fractions were collected and concentrated using a 100-kDa MWCO Vivaspin 500 column (Sartorius).

Purification of SusCDE complexes with a N-terminal Twin-Strep tag on SusC was carried out by the same protocol.

### Peptide mass fingerprinting

Samples were excised from Coomassie-stained gels. For whole sample proteomic analysis, SDS–PAGE was carried out only until the sample had fully entered the gel and the protein smear at the top of the gel was excised. Samples were subject to in-gel trypsin digestion and electrospray mass spectrometry at the Advanced Proteomics Facility (University of Oxford, UK).

### Immunoblotting

Immunoblotting was carried out as previously described^19^. Antibodies against BAM_*Fj*_ subunits, Sus proteins and SkpA were raised in rabbits against His-tagged recombinant proteins produced using the plasmids listed in Supplementary Table 3. Antiserum against OmpA^34^ was provided by S. Shibata and antiserum against SprF^36^ by M. McBride. The following commercial antisera were used: anti-Strep-tag (34850 Qiagen), anti-GroEL (G6532 Merck), anti-ALFA-Tag (N1582 Synaptic Systems GmbH), anti-His-tag (H1029-100UL Merck Life Science), anti-HA-tag (26183 Thermo Fisher Scientific), anti-mouse IgG peroxidase conjugate (A4416 Merck) and anti-rabbit IgG peroxidase conjugate (31462 Pierce). Antibodies were used at the following dilutions: anti-His-tag and anti-HA-tag, 1:1,000; anti-SprF, 1:2,500; anti-BamH, anti-BamM, anti-BamP, anti-SusC, anti-SusD, anti-SusE, anti-SkpA, anti-Strep-tag and anti-ALFA-tag, 1:3,000; anti-OmpA and anti-GroEL, 1:50,000.

Original uncropped gels and immunoblots are shown in Supplementary Fig. 2.

### Darobactin inhibition experiments

*E. coli* or *F. johnsoniae* strains were cultured, respectively, in LB and CYE medium (supplemented with erythromycin if carrying p^TS^BamP or p^TS^BamP4 plasmids). Five-millilitre starter cultures were grown aerobically overnight at 30 °C, then diluted into 5 ml fresh medium to OD_600_ = 0.02 and then grown to OD_600_ between 0.6 to 0.8. The cultures were then diluted with fresh medium to OD_600_ = 0.006. Fifty microlitre aliquots were transferred into a 96-well plate and mixed with 50 µl of the required concentration of darobactin solution in growth medium. The minimum inhibitory concentration (MIC) for darobactin was assessed after overnight incubation at 30 °C in a CLARIOstarPlus plate reader.

### BamP pull-downs

Strains with pCP11-derived plasmids expressing N-terminal Twin-Strep-tagged BamP or BamP4 under the control of a *remA* promoter were grown aerobically overnight at 30 °C in erythromycin-supplemented CYE medium. The culture was diluted into 100 ml fresh medium to OD_600_ = 0.02 and grown to an OD_600_ = 0.8–1.0. Cells were then collected by centrifugation at 8,000*g* for 10 min and resuspended in 3 ml of buffer W containing 30 µg ml^−1^ DNase I, 400 µg ml^−1^ lysosome and 1 mM PMSF. The cells were incubated for 30 min at 4 °C, and then lysed by sonication for 3 min on ice using a Sonics Vibra Cell Ultrasonic Processor VCX 130 with a 6 mm probe at 40% amplitude, with a 10 s on to 10 s off cycle. Unbroken cells were removed by centrifugation at 20,000*g* for 20 min. The supernatant fraction was then centrifuged at 200,000*g* for 1 h to pellet total membranes. The membrane pellets were resuspended to a protein concentration of 6.5 mg ml^−1^ with buffer W and solubilized by incubation with 1% (w/v) LMNG for 2 h. Insoluble material was removed by centrifugation at 230,000*g* for 1 h, and the recovered supernatant supplemented with 1% BioLock solution before mixing with 50 µl Strep-TactinXT 4Flow lbeads (IBA Lifesciences) that had been equilibrated in buffer WD. Samples were rotated slowly at 4 °C for 2 h and then transferred into Mini Bio-Spin Chromatography columns (Bio-Rad, 7326207), and centrifugation at 100*g* for 1 min. The beads were washed 3 times with 250 µl buffer WD and bound proteins then eluted with 150 µl of 1× Strep-TactinXT BXT buffer containing 0.01% LMNG. The elute was concentrated to 30 µl using a 10 kDa MWCO Vivispin500 centrifugal concentrator (VS0102, Sartorius).

### BAM_*Fj*_ subunit depletion experiments

The desired depletion strain was grown overnight in CYE medium supplemented with 0.2 µg ml^−1^ aTC. Cells from 1 ml of the overnight culture were collected, washed once in 1 ml CYE, and resuspended in 1 ml of CYE medium. Cells from this sample were then used to inoculate 15 ml of CYE medium, either with or without 0.2 µg ml^−1^ aTC, to OD_600_ = 0.02. The cells were then cultured aerobically at 30 °C and cell samples collected into SDS sample buffer every 2 h for subsequent analysis by immunoblotting. Samples for imaging or membrane preparation were collected and analysed as detailed below.

To purify BamA complexes after depleting the essential BamG or BamH subunits, a 200 ml overnight culture of the appropriate strain grown in the presence of 0.2 µg ml^−1^ aTC was collected and resuspended in the same volume of fresh CYE medium without aTC. This sample was used to inoculation 8 l of CYE without aTC to OD_600_ = 0.1 which was then cultured aerobically at 30 °C for 6 h. Cells were collected and BamA complexes processed for purification as described above.

### Microscopic analysis of cells during BAM subunit depletions

Live cells were imaged directly in growth medium by spotting samples taken from depletion cultures onto a 1% agarose pad prepared in PY2 medium. Phase contrast images were acquired on an inverted fluorescence microscope (Ti-E, Nikon) equipped with a perfect focus system, a 100× NA 1.4 oil immersion objective, a motorized stage, and a sCMOS camera (Orca Flash 4, Hamamatsu).

For transmission electron microscopy, cells were collected at the required time points during depletion by centrifugation at 8,000*g* for 5 min. After carefully removing the supernatant, cell pellets were gently resuspended in 1 ml of fixative solution (2.5% glutaraldehyde, 4% formaldehyde in 0.1 M PIPES buffer, pH 7.4) and incubated at room temperature for 1 h. Following fixation cells were washed with TEM buffer (100 mM PIPES NaOH pH 7.2), treated with TEM buffer containing 50 mM glycine, washed again in TEM buffer, and then subjected to secondary fixation with TEM buffer containing 1% (w/v) osmium tetroxide and 1.5% (w/v) potassium ferrocyanide. Samples were then washed extensively with Milli-Q water, stained with aqueous 0.5% (w/v) uranyl acetate overnight, then washed again with Milli-Q water. The samples were dehydrated through an ethanol series and infiltrated with and embedded in TAAB low viscosity epoxy resin ahead of polymerization at 60 °C for 24 h. Sections of 90 nm were cut from the resin blocks using a Leica UC7 Ultramicrotome and collected onto 3 mm copper grids. The sections were then post-stained with lead citrate and imaged using a JEOL Flash 120 kV TEM equipped with a Gatan Rio camera.

### Whole-membrane proteomics

Fifteen millilitres of cells at the 6 h time point of the standard depletion experiment were collected by centrifugation at 8,000*g* for 5 min at 4 °C. The cells were resuspended in 1 ml of buffer W and lysed on ice using a probe sonicator (Sonics Vibra Cell, probe 630-0422) at 40% power by 12 repeats of a 10 s on/10 s off pulse cycle. After lysis, the samples were centrifuged at 20,000*g* for 20 min at 4 °C to remove cell debris. The supernatant was then centrifuged at 135,000*g* for 45 min at 4 °C to pellet the membranes. The membranes were resuspended in buffer W and the protein contents of the samples normalized by A_280 nm_. The samples were run together on SDS–PAGE gels and stained with Coomassie Blue (Extended Data Fig. 7i) to confirm that normalization had been correctly implemented. Statistical methods were not used to determine sample size. Randomization and blinding were not used.

Membrane fractions were resuspended in lysis buffer containing 1% SDS, 0.1 M ammonium bicarbonate pH 8.0. Samples were sonicated for 5× 15 s in a water bath with 15 s incubations on ice between each pulse cycle. The samples were clarified by centrifugation at 17,500*g* for 30 min and 50 µg of total protein lysate was taken for analysis. Samples were reduced for 30 min using 10 mM tris(2-carboxyethyl)phosphine (TCEP) followed by alkylation for 30 min in the dark using 2-chloroacetamide. SpeedBeads Magnetic Carboxylate Modified Particles (GE Healthcare) were mixed with the sample in a 10 volumes beads: 1 volume sample ratio and the samples shaken for 10 min at 1,000 rpm. The beads were then washed twice with 70% ethanol followed by 100% acetonitrile. This procedure was repeated 8 times. 100 mM ammonium bicarbonate was added to the washed beads and pre-digestion with endoprotease LysC (Wako; 1:100) was carried out at 37 °C for 2 h. This was followed by 16 h digestion with trypsin (Promega, 1:40) at 37 °C. The supernatant was collected and any remaining bound peptides were eluted from the beads using 2% dimethyl sulfoxide (DMSO). Digested peptides were loaded onto C18 stage tips, pre-activated with 100% acetonitrile and 0.1% formic acid and centrifuged at 4000 rpm. The tips were then washed with 0.1% formic acid and eluted in 50% acetonitrile/0.1% formic acid. Eluted peptides were dried in a speed-vac.

Peptide analysis employed a Thermofisher Scientific Ultimate RSLC 3000 nano liquid chromatography system coupled in-line to a Q Exactive mass spectrometer equipped with an Easy-Spray source (Thermofisher Scientific). Peptides were separated using an Easy-Spray RSLC C18 column (75 µm internal diameter, 50 cm length, Thermofisher Scientific) using a 60 min linear 15% to 35% solvent B (0.1% formic acid in acetonitrile) gradient at a flow rate 200 nl min^−1^. The raw data were acquired on the mass spectrometer in a data-dependent acquisition (DDA) mode. Full-scan mass spectra were acquired in the Orbitrap (Scan range 350–1,500 *m*/*z*, resolution 70,000, AGC target 3 × 10^6^, maximum injection time 50 ms). The 10 most intense peaks were selected for higher-energy collision dissociation (HCD) fragmentation at 30% of normalized collision energy. HCD spectra were acquired in the Orbitrap at resolution 17,500, AGC target 5 × 10^4^, maximum injection time 120 ms with fixed mass at 180 *m*/*z*.

Mass spectrometry data were analysed using MaxQuant 2.5.1.0 as previously described^51^ to obtain label-free quantification values that were then used for data processing in Perseus 2.1.3.0^52^. Label-free quantification values were log_2_-transformed and categorically grouped by replicates. Rows were filtered based on two valid values in each group and then missing values were replaced using a normal distribution with a width of 0.3 and down shift of 1.8 (default values). Then, dataset was normalized by subtracting the medians of each sample. After visually verifying a normal distribution and a linear correlation, sample pairs were subjected to a two-tailed *t*-test using a false discovery rate (FDR) of 0.1 and a *S*_0_ of 0.1 to define a threshold of statistical significance. Proteins were represented in a volcano plot, according to the log_2_ of their enrichment and the −log_10_ of the *t*-test *P* value.

An ANOVA test was carried out for indicated groups of proteins using the Benjamini–Hochberg method with a FDR of 0.05 for truncation. Then, a post hoc Tukey’s honest significant difference test for one-way ANOVA using a FDR of 0.05 was carried out. Proteins were then filtered by ANOVA significance and by category to represent in a heat map their honest significant difference scores, as indicated.

A batch normalization using empirical Bayes method was carried out with the ComBat script^53^ for PerseusR package 0.3.4^54^ to make the heat map for all depletions (Extended Data Fig. 8). Then, samples were subjected to the statistical test previously described.

The proteins obtained from the mass spectrometry experiments were categorized as follows. Proteins with signal peptides or lipoprotein signal peptides were first extracted using SignalP 6.0^55^ to obtain datasets containing only OM plus periplasmic proteins, or lipoproteins, respectively. Proteins were then manually sorted to the categories OMP or SLP. This sorting was carried out using Uniprot entry data that included AlphaFold^23^ models. Lipoproteins were only classified as SLPs if they were either SusD homologues or if they were found at a locus coding SusCD systems.

### Determination of cell surface exposure of SusE

The strain for analysis was transformed with plasmid pXL184 which expresses His-tagged SusE. The cells were then grown overnight in CYE supplemented with erythromycin, and for BAM subunit depletion strains with 0.2 µg ml^−1^ aTC. Cells were collected, resuspended in CYE medium, and then used to inoculate 10 ml of erythromycin-containing CYE medium to OD_600_ = 0.02, supplementing with 0.2 µg ml^−1^ aTC as required. The cells were cultured for 6 h before being collected by centrifugation and resuspended in phosphate buffered saline (PBS) containing 10 mM MgCl_2_ to a total volume of 80 µl and OD_600_ = 1. Samples were supplemented as appropriate with 200 μg ml^−1^ proteinase K (Thermo Fisher) and 1% (v/v) Triton X-100 (Merck) and incubated for 20 min at room temperature. Reactions were stopped by the addition of 5 mM PMSF (ITW Reagents) followed by incubation at 100 °C for 5 min, addition of SDS–PAGE sample buffer, and further incubation at 100 °C for 5 min before analysis by immunoblotting.

### Isolation of outer membrane vesicle fraction

The isolation of outer membrane vesicles (OMVs) was performed essentially as in ref. ^38^. In brief, cells were separated from culture supernatant by centrifugation at 8,000*g* for 5 min and the pellets reserved as the whole-cell fraction. Culture supernatant from the equivalent of 2 ml of culture at OD_600_ = 1 was filtered through a 0.2 µm filter (MilliporeSigma, SLGPR33RB) and concentrated using a 100 kDa molecular weight cut-off Amicon Ultra-4 centrifugal filter (MilliporeSigma, UFC810096) to produce the OMV fraction. Samples were adjusted to equal volume before analysis by immunoblotting.

### Isolation of a spontaneous suppressor of BamH depletion

The BamH depletion strain XLFJ_1140 was grown overnight in CYE medium supplied with aTC. One millilitre of cells was collected by centrifugation at 8,000*g* for 3 min, washed once with CYE and then diluted to a starting OD_600_ = 0.2 in 10 ml fresh CYE medium without aTC. After culturing for 6 h, cells were diluted 1:200 into fresh CYE medium without aTC and cultured for a further 2 days before plating on CYE agar to obtain single colonies. Individual clones were cultured in parallel with and without aTC in CYE and the expression of BamH analysed by whole-cell immunoblotting. Clones that grew without aTC but still expressed BamH only following aTC induction (showing that they were not constitutively de-repressed for BamH synthesis) were subjected to genome sequencing (Plasmidsaurus). This identified the potential suppressor mutation *bamA*^*Q801K*^, which was introduced into a BAM wild-type background, followed by successive deletions of *bamH* and *bamH2* to produce the *bamH*^*sup*^ strain XLFJ_1198.

### Cryo-EM sample preparation and imaging

Four microlitres of either fraction A (for the BAM_*Fj*_ complex, 1.3 mg ml^−1^) or fraction B (for the BamAP complex, 1.3 mg ml^−1^) of the BAM_*Fj*_ preparation (Fig. 1a), or of the BamP-deleted BAM complex (ΔBamP complex, 1.2 mg ml^−1^) was adsorbed onto glow-discharged holey carbon-coated grids (Quantifoil 300 mesh, Au R1.2/1.3) for 10 s. Grids were blotted for 2 s at 10 °C, 100% humidity and frozen in liquid ethane using a Vitrobot Mark IV (Thermo Fisher Scientific).

Movies were collected in counted mode, in Electron Event Representation (EER) format, on a CFEG-equipped Titan Krios G4 (Thermo Fisher Scientific) operating at 300 kV with a Selectris X imaging filter (Thermo Fisher Scientific) and slit width of 10 eV, at ×165,000 magnification on a Falcon 4i direct detection camera (Thermo Fisher Scientific), corresponding to a calibrated pixel size of 0.732 Å. Movies were collected at a total dose ranging between 52.0–60.3 e^−^ Å^−2^ (Extended Data Table 1), fractionated to ~1.0 e^−^ Å^−2^ per fraction for motion correction.

### Cryo-EM data processing

Patched motion correction, contrast transfer function (CTF) parameter estimation, particle picking, extraction and initial 2D classification were performed in SIMPLE 3.01^56^. All downstream processing was carried out in cryoSPARC 4.5.3^57^ or RELION 4.03^58^, using the csparc2star.py script within UCSF pyem 0.5^59^ to convert between formats. Global resolution was estimated from gold-standard Fourier shell correlations (FSCs) using the 0.143 criterion and local resolution estimation was calculated within cryoSPARC.

The cryo-EM processing workflow for the BAM_*Fj*_ complex is outlined in Extended Data Fig. 1. In brief, particles were subjected to one round of reference-free 2D classification (*k* = 200) using a 240 Å soft circular mask within cryoSPARC resulting in the selection of 2,153,927 clean particles. A subset of these particles (180,179) was subjected to multi-class ab initio reconstruction using a maximum resolution cut-off of 7 Å, generating 4 volumes. These volumes were lowpass-filtered to 20 Å and used as references in a heterogeneous refinement against the full 2D-cleaned particle set. Particles (903,299) from the most populated and structured class were selected and non-uniform refined against their corresponding volume lowpass-filtered to 15 Å, generating a 3.0 Å map. Bayesian polishing in RELION followed by duplicate particle removal generated a 2.5 Å map after non-uniform refinement, which could be further improved to 2.3 Å after local and global CTF refinement (fitting beam tilt and trefoil only). These particles were then subjected to heterogeneous refinement against four compositionally distinct volumes previously generated by RELION 3D classification (*k* = 8, 3.75° sampling) of a particle subset of pre-polished particles. Particles (274,708) belonging to the class with strong BamD and POTRA densities were selected and non-uniform refined against their corresponding volume, generating a 2.4 Å map. Additional alignment-free 3D classification in RELION was performed (*k* = 6) using a soft mask covering BamD and the BamA POTRA domains yielding a class with stronger density. Particles (55,795) from this class were selected and non-uniform refined against a previous volume lowpass-filtered to 15 Å, generating a consensus 2.7 Å volume. Local refinements were performed against the consensus volume (lowpass-filtered to 7 Å) using soft masks covering the BamD/POTRA domains or extracellular density, yielding 3.2 Å and 2.7 Å volumes, respectively. ChimeraX^60^ was used to generate a composite map from the consensus and individual focused maps.

The cryo-EM processing workflow for the BamAP complex is outlined in Extended Data Fig. 6. Two datasets were collected for this sample. In the first dataset particles were subjected to two rounds of reference-free 2D classification (*k* = 200) using a 200 Å soft circular mask resulting in the selection of 979,474 clean particles. These particles were then subjected to multi-class ab initio reconstruction (*k* = 4) using a maximum resolution cut-off of 8 Å, generating 4 volumes. Particles (514,326) belonging to the 2 most prominent volumes were combined and non-uniform refined against one of their corresponding volumes, lowpass-filtered to 15 Å, generating a 3.7 Å volume. The second particle dataset underwent four rounds of 2D classification (*k* = 200, 200 Å soft circular mask) followed by multi-class ab initio reconstruction using a maximum resolution cut-off of 7 Å, generating 6 volumes. Particles (438,412) from the most populated class were selected and refined against their corresponding volume lowpass-filtered to 15 Å, generating a 3.7 Å volume. Particles from both datasets were independently polished within RELION, combined, and non-uniform refined, fitting per-particle CTF parameters, yielding a 3.5 Å map. Alignment-free 3D classification was subsequently performed within cryoSPARC (*k* = 6), using a soft mask covering the full protein density of the complex. Particles (96,076) from the class demonstrating strong density for the N-terminal domain of BamP were selected and non-uniform refined against their corresponding volume, lowpass-filtered to 15 Å, generating a 3.7 Å map.

The cryo-EM processing workflow for the ΔBamP complex is outlined in Extended Data Fig. 5. In brief, particles were subjected to two rounds of reference-free 2D classification (*k* = 200) using a 180 Å soft circular mask within cryoSPARC resulting in the selection of 1,177,554 clean particles. These particles were then subjected to multi-class ab initio reconstruction using a maximum resolution cut-off of 6 Å, generating 6 volumes. Particles from volume classes containing BamA barrels were independently non-uniform refined against their corresponding volume, lowpass-filtered to 15 Å. These particles were subsequently combined and refined against a volume (lowpass-filtered to 15 Å) from the most populated class, generating a 3.6 Å consensus volume. Bayesian polishing in RELION followed by non-uniform refinement and fitting of per-particle CTF parameters plus beam tilt and trefoil generated a 3.5 Å map. Map quality was further improved by non-uniform refinement of a cleaner particle set (534,368 particles) generated by an additional round of 2D classification (*k* = 100, 180 Å soft circular mask), despite no increase in nominal resolution. A second β-barrel could be resolved in map density at low contour level (0.08). Attempts to improve map quality for this partner β-barrel, through extensive 3D classification and local refinement schemes, did not improve map quality for this region.

### Model building, structure refinement and figure preparation

Iterative model building and real-space refinement using secondary structure, rotamer, and Ramachandran restraints was performed in Coot v0.9^61^ and Phenix 1.21^62^, respectively. Validation was performed in Molprobity 4.5.2^63^ within Phenix. Cryo-EM data collection, image processing and structure refinement statistics are listed in Extended Data Table 1. Figures were prepared using UCSF ChimeraX v.1.9^60^.

### Reporting summary

Further information on research design is available in the Nature Portfolio Reporting Summary linked to this article.

## Online content

Any methods, additional references, Nature Portfolio reporting summaries, source data, extended data, supplementary information, acknowledgements, peer review information; details of author contributions and competing interests; and statements of data and code availability are available at 10.1038/s41586-025-09532-8.

## Supplementary information

Supplementary Information Supplementary Fig. 1, Supplementary Tables 1–4 and references

Reporting Summary

Supplementary Figure 2 Uncropped gels and immunoblots. See separate source data file Supplementary Fig. 1.

Supplementary Data 1 Hybrid model of the BAM_*Fj*_ complex. The experimental model deposited as PDB: 9N2D is extended by AlphaFold structures for the parts of the complex that are insufficiently resolved in the electron microscopy density to allow confident model building (the POTRA 1–3 domains of BamA and C-terminal portion of BamP)

## Source data

Source Data Fig. 1

Source Data Fig. 4

Source Data Fig. 5

Source Data Extended Data Fig. 5

Source Data Extended Data Fig. 7

Source Data Extended Data Fig. 8

Source Data Extended Data Fig. 9

## Figures and Tables

**Extended Data Table 1:**
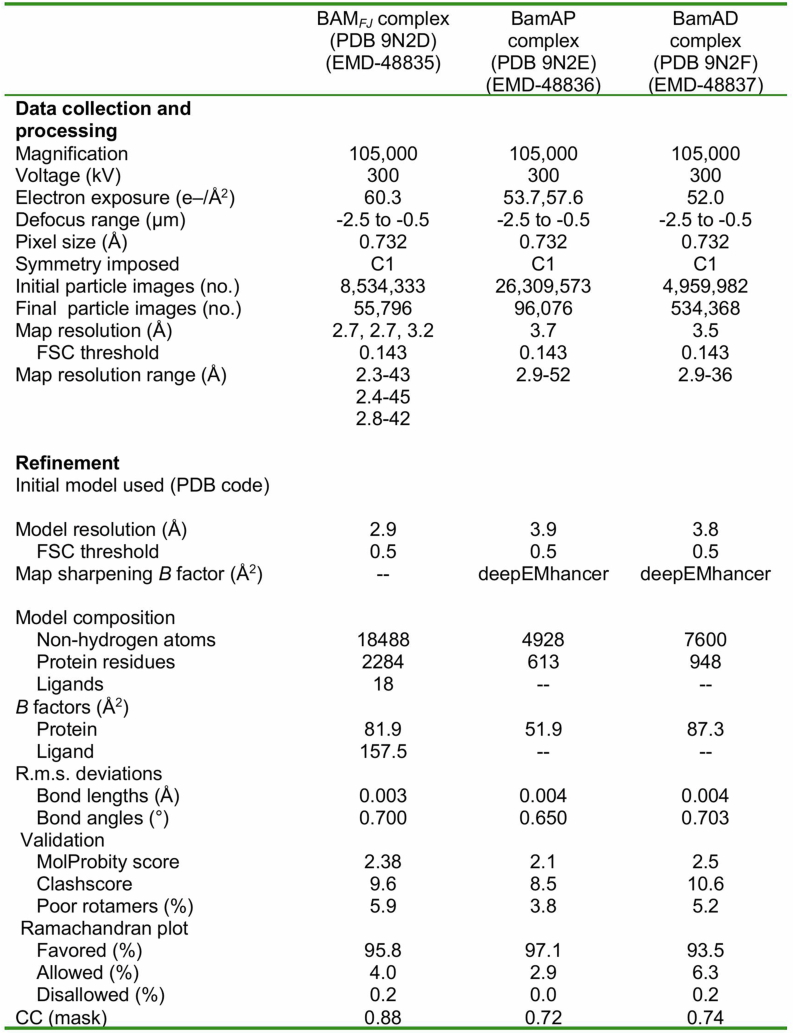
Cryo-EM data collection, refinement and validation statistics

**Extended Data Table 2:**
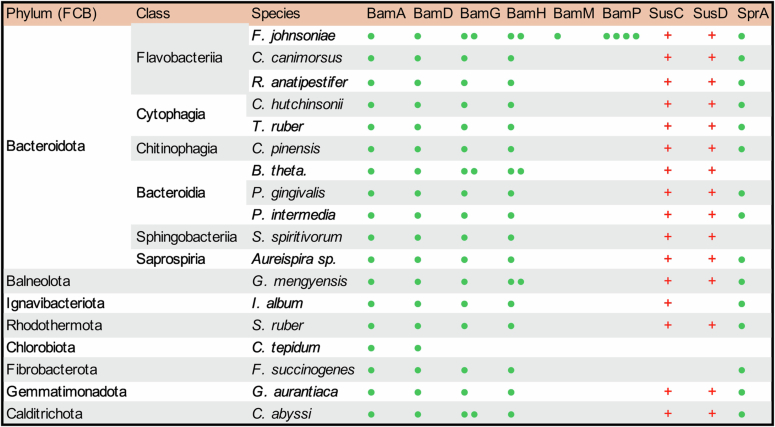
Phylogenetic distribution of BAM_*FJ*_ subunits

Distribution of BAM_*FJ*_ subunit homologues within representative members of the Bacteroidota and wider FCB superphylum. Each copy of a coding gene is indicated by a green dot. The presence of members of the SusC and SusD protein families (red +) and the 36-stranded T9SS translocon barrel SprA^15^ are also shown. See also Supplementary Table 1.

## Data Availability

Cryo-EM density maps and atomic coordinates have been deposited in the Electron Microscopy Data Bank (EMDB) with the following accession numbers: EMD-48835 (BAM_*Fj*_ composite map), EMD-48832 (BAM_*Fj*_ consensus map), EMD-48833 (BAM_*Fj*_ BamHM-focused map), EMD-48834 (BAM_*Fj*_ BamADP-focused map), EMD-48836 (BamAP complex) and EMD-48837 (BamAD complex). Atomic coordinates have been deposited in the Protein Data Bank (PDB) with the following accession numbers: 9N2D (BAM_*Fj*_ complex), 9N2E (BamAP complex) and 9N2F (BamAD complex). The hybrid model of the BAM_*Fj*_ complex is provided in Supplementary Data 1. Raw proteomics data have been deposited in the PRIDE database with the accession PXD065907. Processed proteomics source data and peptide fingerprinting source data are provided with this paper. Uncropped gels and immunoblots are in Supplementary Fig. 2. Requests for materials should be addressed to B.C.B. Source data are provided with this paper.
