# Supplementary material for: A new paradigm for outer membrane protein biogenesis in the Bacteroidota
Source: Nature. 2025 Oct 1;647(8089):479–87. doi: 10.1038/s41586-025-09532-8 (PMC12611786; doi:10.1038/s41586-025-09532-8)
Supplement: Supplementary file 1 — Supplementary Fig. 1, Supplementary Tables 1–4 and references [file 41586_2025_9532_MOESM1_ESM.pdf]

nature portfolio

https://doi.org/10.1038/s41586-025-09532-8

Supplementary information

A new paradigm for outer membrane protein biogenesis in the Bacteroidota

In the format provided by the authors and unedited

Supplementary Information For:

A new paradigm for outer membrane protein biogenesis in the Bacteroidota

Xiaolong Liu[1§*], Luis Orenday Tapia[1§], Justin C. Deme[2§], Susan M. Lea[2,3*], and Ben C. Berks[1*]

[1] Department of Biochemistry, University of Oxford, Oxford, United Kingdom.

[2] Center for Structural Biology, Center for Cancer Research, National Cancer Institute, Frederick, Maryland, United States of America.

[3] Structural Biology, St Jude Children's Research Hospital, Memphis, Tennessee, United States of America.

* Corresponding authors. Email ben.berks@bioch.ox.ac.uk, susan.lea@stjude.org, xiaolong.liu@bioch.ox.ac.uk

§ These authors contributed equally to this work.

Table of Contents

Supplementary Fig. 1. AlphaFold predictions of the structure of BamA barrels from representative Bacteroidota………………………………….. ………………..3

Supplementary Fig. 2. Uncropped gels and immunoblots……………..…………..4

Supplementary Table 1. Accession numbers of BamA, BamD, BamG, and BamH proteins analyzed in Extended Data Table 2…………………………...……......5

Supplementary Table 2. Bacterial strains used in this study……………………….6

Supplementary Table 3. Plasmids used in this study...........................……......8

Supplementary Table 4. Primers used in this study………………………………10

Supplementary references………..……………………………………………….16

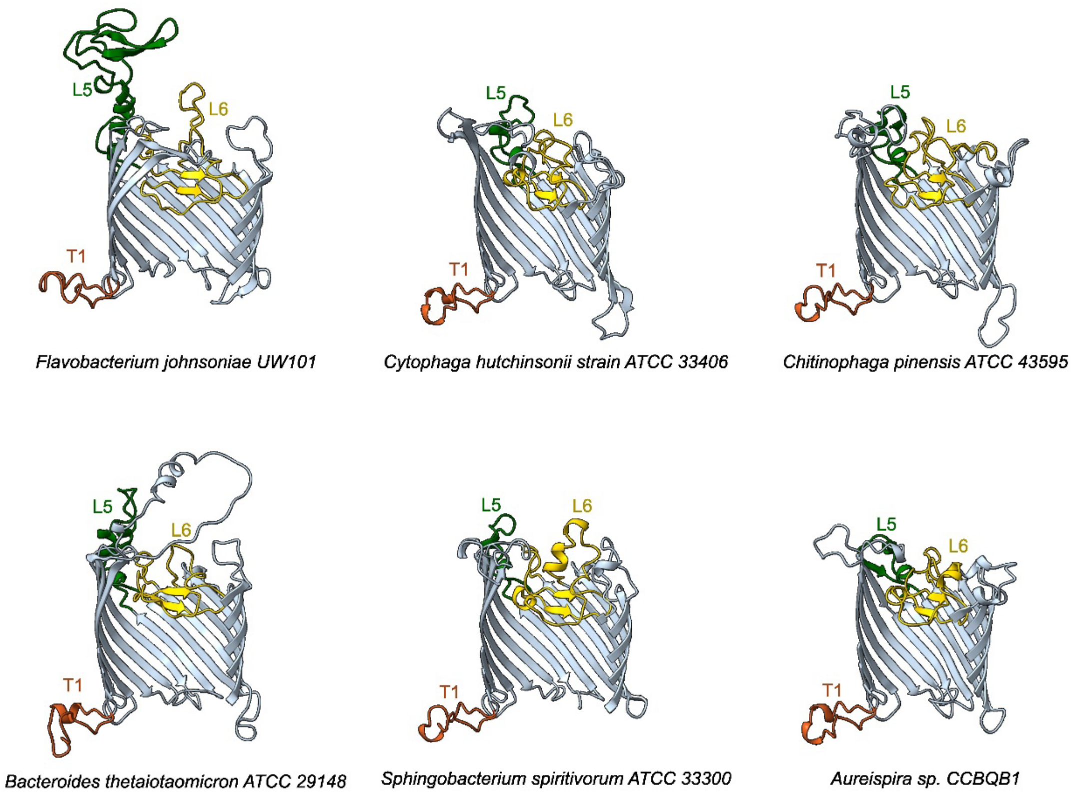

Supplementary Figure 1│AlphaFold predictions of the structure of BamA barrels from representative Bacteroidota. The proteins analyzed are from selected species in Extended Data Table 2 and are compared with the experimental structure of the *F. johnsoniae* protein. Loop 5 (L5) is shown in green, loop 6 (L6) in yellow, and turn 1 (T1) in orange. The strands closest to the viewer have been removed to reveal Loop 6.

Supplementary Figure 2│ Uncropped gels and immunoblots. See separate source data file Supplementary Figure 1.

Supplementary Table 1│Accession numbers of BamA, BamD, BamG, and BamH proteins analyzed in Extended Data Table 2.

Species	BamA	BamD	BamG	BamH
Flavobacterium johnsoniae UW101	Fjoh_1690	Fjoh_3469	Fjoh_1412; Fjoh_1686	Fjoh_0823, Fjoh_1685
Capnocytophaga canimorsus Cc5	Ccan_09070	Ccan_09420	Ccan_02540	Ccan_06570
Riemerella anatipestifer DSM 15868	RA0C_1327	RA0C_1616	RA0C_0295	RA0C_1937
Cytophaga hutchinsonii ATCC 33406	CHU_3412	CHU_3017	CHU_3271	CHU_3837
Thermoflexibacter ruber ATCC 23103	SAMN04488541_1004111	SAMN04488541_1009104	SAMN04488541_100378	SAMN04488541_103619
Chitinophaga pinensis ATCC 43595	Cpin_3279	Cpin_6968	Cpin_1839	Cpin_1840
Bacteroides thetaiotaomicron ATCC 29148	BT_3725	BT_0573	BT_4367, BT_1785	BT_4306, BT_1786
Porphyromonas gingivalis W83	PG_0191	PG_1215	PG_0448	PG_1835
Prevotella intermedia ATCC 25611	BWX39_00670	BWX39_04985	BWX39_09620	BWX39_09115
Sphingobacterium spiritivorum ATCC 33300	HMPREF0765_4825	HMPREF0765_0270	HMPREF0765_2828	HMPREF0765_0180
Aureispira sp. CCB-QB1	WP_052599347.1	WP_052598710.1	WP_052595627.1	WP_081909491.1
Gracilimonas mengyensis DSM 21985	SAMN06265219_11353	SAMN06265219_101289	SAMN06265219_106164	SAMN06265219_103120, SAMN06265219_106163
Ignavibacterium album JCM 16511	IALB_0497	IALB_3077	IALB_1455	IALB_1454
Salinibacter ruber DSM 13855	SRU_1846	SRU_2073	SRU_0789	SRU_0790
Chlorobaculum tepidum DSM 12025	CT0266	CT0017		
Fibrobacter succinogenes S85	FSU_2654	WP_041917861.1	FSU_1156	FSU_1157
Gemmatimonas aurantiaca strain DSM 14586	GAU_1585	GAU_1963	GAU_1453	GAU_1452
Caldithrix abyssi DSM 13497	Calab_3667	Cabys_2838	Cabys_4206, Cabys_3919	Cabys_4207

Supplementary Table 2 │ Bacterial strains used in this study.

Strain	Genotype and Details	Reference
E. coli		
NEB® 5-α	*fhuA2 Δ(argF-lacZ)U169 phoA glnV44 Φ80 Δ(lacZ)M15 gyrA96 recA1 relA1 endA1 thi-1 hsdR17*	NEB
MG1655	*F^- lambda$^-$ ilvG$^-$ rfb$^-$50 rph-1*	67
HB101 Helper	Strain used with pRK2013 for triparental conjugation	36
BL21 (DE3)	*F^- ompT hsdS$_B$ (r_B^-, m_B^-) galdcmrne131 (DE3)*	Invitrogen
F. johnsoniae		
UW101	Wild-type *F. johnsoniae* strain	46
FI_058	UW101 Δ*porV* Δ*plug*	15
XLFJ_000	UW101 Δ*fjoh_1945*	This study
XLFJ_963	*Δfjoh_1945 twinstrep-bamA (fjoh1690)*	This study
XLFJ_988	*Δfjoh_1945 twinstrep-bamA ΔbamP* (*fjoh_1771)*	This study
XLFJ_998	*Δfjoh_1945 twinstrep-bamA ΔbamM (fjoh_0050)*	This study
XLFJ_1016	*Δfjoh_1945 twinstrep-bamA ΔbamP-P3 (fjoh_1769-1771)*	This study
XLFJ_1027	*Δfjoh_1945 twinstrep-bamA ΔbamP-P3 ΔbamP4 (fjoh_2401)*	This study
XLFJ_1078	*Δfjoh_1945 twinstrep-bamA bamG-SW3-α (fjoh_1412)*	This study
XLFJ_1090	*Δfjoh_1945 twinstrep-bamA ΔbamP ΔbamM*	This study
XLFJ_1093	*Δfjoh_1945 twinstrep-bamA ΔbamP4*	
XLFJ_1095	*tetR-P_{ompA} $P_{ompAinduc}$-NanoLuc*	This study
XLFJ_1100	*tetR-P_{ompA} $P_{fjoh\_0824induc}$ -NanoLuc*	This study
XLFJ_1115	*Δfjoh_1945 twinstrep-bamA tetR-P_{ompA} $P_{ompAinduc}$ -bamG-SW3-α ΔbamG*	This study
XLFJ_1122	*Δfjoh_1945 twinstrep-bamA tetR-P_{ompA} $P_{ompAinduc}$-bamH ΔbamH*	This study
XLFJ_1129	*Δfjoh_1945 tetR-P_{ompA} $P_{ompAinduc}$- twinstrep-bamA ΔbamA bamG-SW3-α*	This study
XLFJ_1130	*Δfjoh_1945 twinstrep-bamA tetR-P_{ompA} $P_{ompAinduc}$ -bamH ΔbamH bamG-SW3-α*	This study
XLFJ_1131	*Δfjoh_1945 twinstrep-bamA tetR-P_{ompA} $P_{ompAinduc}$-bamG-SW3-α ΔbamG/pXL184*	This study

XLFJ_1139	*Δfjoh_1945 tetR-P_{ompA} $P_{ompAinduc}$-twinstrep-bamA ΔbamA bamG-SW3-α/pXL184*	This study
XLFJ_1140	*Δfjoh_1945 twinstrep-bamA tetR-P_{ompA} $P_{fjoh\_0824induc}$-bamH ΔbamH bamG-SW3-α*	This study
XLFJ_1144	*Δfjoh_1945 twinstrep-bamA tetR-P_{ompA} $P_{fjoh\_0824induc}$-bamH ΔbamH bamG-SW3-α/pXL184*	This study
XLFJ_1162	*Δfjoh_1945 twinstrep-bamA bamG-SW3-α ΔbamG2 (fjoh_1686)*	This study
XLFJ_1174	*Δfjoh_1945 HA-bamA bamG-SW3-α*	This study
XLFJ_1180	*Δfjoh_1945 twinstrep-bamA tetR-P_{ompA} $P_{fjoh\_0824induc}$ -bamH ΔbamH bamG-SW3-α ΔbamH2 (fjoh_1685)*	This study
XLFJ_1183	*Δfjoh_1945 twinstrep-$bamA^{Q801K}$ bamG-SW3-α*	This study
XLFJ_1187	*Δfjoh_1945 twinstrep-$bamA^{Q801K}$ bamG-SW3-α ΔbamH*	This study
XLFJ_1198	*Δfjoh_1945 twinstrep-$bamA^{Q801K}$ bamG-SW3-α ΔbamH ΔbamH2*	This study
XLFJ_1200	*Δfjoh_1945 twinstrep-bamA ΔbamH2*	This study
XLFJ_1201	*Δfjoh_1945 twinstrep-bamA ΔbamG2*	This study
XLFJ_1207	*Δfjoh_1945 twinstrep-$bamA^{Q801K}$ bamG-SW3-α ΔbamH ΔbamH2/pXL184*	This study
XLFJ_1208	*Δfjoh_1945 HA-bamA ΔbamP*	This study
XLFJ_1211	*Δfjoh_1945 HA-$bamA^{Q801K}$ bamG-SW3-α ΔbamH ΔbamH2*	This study
XLFJ_1213	*Δfjoh_1945 twinstrep-bamA bamG-SW3-α ΔbamH2*	This study
XLFJ_1214	*Δfjoh_1945 twinstrep-$bamA^{Q801K}$ bamG-SW3-α ΔbamH2*	This study
XLFJ_1215	*Δfjoh_1945 twinstrep-$bamA^{Q801K}$ bamGF-SW3-α ΔbamG ΔbamH2 ΔbamM*	This study
XLFJ_1217	*Δfjoh_1945 twinstrep-$bamA^{Q801K}$ bamG-SW3-α ΔbamH ΔbamH2 Δfjoh_1769-1771*	This study
XLFJ_1266	*Δfjoh_1945 twinstrep-bamA ΔbamP-P3 ΔbamP4 ΔporV*	This study
XLFJ_1271	*Δfjoh_1945 twinstrep-bamA ΔbamP-P3 ΔbamP4 ΔporV Δplug*	This study
sLO55	*Δfjoh_1945 twinstrep-0403*	This study
sLO77	*Δfjoh_1945 HA-bamA bamG-SW3-α twinstrep-fjoh_0403(susC)*	This study
sLO78	*Δfjoh_1945 HA-$bamA^{Q801K}$ bamG-SW3-α ΔbamH Δfjoh_1685 twinstrep-fjoh_0403(susC)*	This study

Supplementary Table 3│Plasmids used in this study.

plasmid	Descriptions	Reference
pYT313	*sacB* containing mobilizable suicide vector; Ap^R (Em^R)	15
pCP11	*E. coli-F. johnsoniae* shuttle plasmid; Apr (Emr)	68
pRK2013	Helper plasmid for triparental conjugation; IncP Tra^+ Km^r	36
pET21a(+)	*E. coli* expression vector, T7 promoter; Ap^r	Merck
pET15b-TEV	*E. coli* expression vector T7 promoter; Ap^r	15
pFL80	pYT313-derived construct used to generate the deletion of *porV*	15
pFL81	pYT313-derived construct used to generate the deletion of *plug*	15
pXL155	pYT313-derived construct used to generate the deletion of *Fjoh1945*	This study
pXL184	pCP11-derived plasmid used to express C-terminally his-tagged SusE under the control of the *ompA* promoter	This study
pXL200	pYT313-derived construct used to generate N-terminally twinstrep tagged *bamA*	This study
pXL201	pYT313-derived construct used to generate the deletion of *fjoh_1685.*	This study
pXL213	pYT313-derived construct used to generate the deletion of *bamM*	This study
pXL214	pYT313-derived construct used to generate the deletion of *bamP*	This study
pXL220	pYT313-derived construct used to generate the deletion of *fjoh1769-1771*	This study
pXL222	pYT313-derived construct used to generate the deletion of *fjoh_2401*	This study
pXL235	pYT313-derived construct used to generate astrain expressing NanoLuc under the control of the aTC-inducible $P_{ompAinduc}$ promoter	This study
pXL239	pYT313-derived construct used to generate the strain expressing NanoLuc under the aTC-inducible $P_{fjoh\_0824induc}$ promoter	This study
pXL243	pYT313-derived construct used to generate the ALFA tagged BamG by insertion after *gly245*	This study
pXL246	pYT313 derived construct used to generate the deletion of *bamA*	This study
pXL247	pYT313-derived construct used to generate the strain expressing N-terminaly twinstrep tagged BamA under the control of the aTC-inducible $P_{ompAinduc}$ promoter	This study

pXL250	pYT313-derived construct used to generate the strain expressing ALFA-tagged BamG under the aTC-inducible $P_{ompAinduc}$ promoter	This study
pXL252	pYT313-derived construct used to generate the deletion of *bamG*	This study
pXL253	pYT313-derived construct used to generate the deletion of *bamH*	This study
pXL256	pYT313-derived construct used to generate the strain expressing BamH under the inducible *ompA* promoter	This study
pXL259	pYT313-derived construct used to generate the strain expressing BamH under the aTC-inducible $P_{fjoh\_0824induc}$ promoter	This study
pXL273	pYT313-derived construct used to generate the deletion of *fjoh_1686*	This study
pXL279	pYT313-derived construct used to generate the $bamA^{Q801K}$ mutant	This study
pXL283	pYT313-derived construct used to delete *bamD*	This study
pXL339	pCP11-derived construct to express N-terminal twinstrep-tagged BamP4 under the P_{remA} promoter	This study
pXL340	pCP11-derived construct to express N-terminal twinstrep-tagged BamP under the P_{remA} promoter	This study
pLO79	pET21-derived vector to heterologously overproduce signal peptide-less BamH with a N-terminal 8His tag	This study
pLO80	pET21-derived vector to heterologously overproduce signal peptide-less BamM with a N-terminal 8His tag	This study
pLO92	pET21-derived vector to heterologously overproduce signal peptide-less BamP with a N-terminal 8His tag	This study
pLO95	pET21-derived vector to heterologously overproduce signal peptide-less SusE with a N-terminal 8His tag	This study
pLO100	pET15b-derived vector to heterologously overproduce signal peptide-less SusD with a N-terminal 8His-SUMO tag	This study
pLO102	pET21-derived vector to heterologously overproduce signal peptide-less SusC N-terminal domain with a N-terminal 8His tag	This study
pLO103	pYT313-derived construct used to generate N-terminally twinstrep tagged *susC*	This study

Supplementary Table 4│Primers used in this study.

oligos	Sequences (5′ to 3′)
FL169	GGATATACCGAAAAAATCGC
priXL01	GGATCCTCTAGAGTCGACCT
priXL02	CCCGAATTTTTCCGCTGCAT
priXL05	CACCAAGGAAAGTCTACACGA
priXL06	CAGGAAACAGCTATGACCAT
priXL51	GTCTACACGAACCCTTTGG
priXL52	TCCACTTTCATATTACGGTTTA
priXL315	TTAATAAATGTATTCTTCTTTATTAGCC
priXL320	CAAGCTTGCATGCCTGCAGGTCGACTCTAGAGGATCCTGGTTGAAAATGGTGAGAAAAT
priXL316	CGAAATGATGGCTAATAAAGAAGAATACATTTATTAAATTTTAAAGCCCCATTTTAATG
priXL317	TGACCCCGAAGCAGGGTTATGCAGCGGAAAAATTCGGGGATACTTTAACTGAAGGCGAA
priXL325	TTGTTGGACACAGAATTCCT
priXL326	GAATCGCTCTTACTTATATAGAGT
priXL364	CAGATTTCTACAACAGTTACATTTGATGCTGACGCAATCCATCACCATCATCACCACCA
priXL366	GAACGCAGCTATTTGTTTTATATATTTTTTCATACTTAATTTTTTTAATTACAATTTAG
priXL367	ATGAAAAAATATATAAAACAAATAGCTG
priXL368	GATTGCGTCAGCATCAAATGT
priXL415	CGCCGAACCTCCCGATCCACCTCCGGAACCTCCACCTTTTTCGAACTGCGGGT GGCTCCATTGAGCTTTTATTTGTGAAAAACTAC
priXL416	GGTGGAGGTTCCGGAGGTGGATCGGGAGGTTCGGCGTGGTCACATCCACAATTTGAGAA GGATAGAGTTCCTTTTGATCAAGG
priXL417	ATAATGACCCCGAAGCAGGGTTATGCAGCGGAAAAATTCGGGGATATTCG ATATCAACAGTTCCAG
priXL418	CAAGCTTGCATGCCTGCAGGTCGACTCTAGAGGATCCACTTCCAGATAACCAGTATCAA
priXL419	TATTTGCCAAAAAAATTGATTCCTTACCAGGAATATCTAAATCAG

priXL420	GATTTAGATATTCCTGGTAAGGAATCAATTTTTTTGGCAAATATAAAAGG
priXL421	ACCCCGAAGCAGGGTTATGCAGCGGAAAAATTCGGGGTTTGTATTTGATCCCTATTCCT
priXL422	AAGCTTGCATGCCTGCAGGTCGACTCTAGAGGATCCGCTCAAACTTTTTAAGTTTGAGC
priXL423	GTTTTTTTTAGCTGCTTCATTTTTGGTTTGTAAAACGAAAATTCG
priXL424	CGAATTTTCGTTTTACAAACCAAAAATGAAGCAGCTAAAAAAAAC
priXL425	TGACCCCGAAGCAGGGTTATGCAGCGGAAAAATTCGGGTGGATCAGATTATCTTGCTCT
priXL426	CAAGCTTGCATGCCTGCAGGTCGACTCTAGAGGATCCTAGTGGTTACGTCTGAAAAATG
priXL429	TGACCCCGAAGCAGGGTTATGCAGCGGAAAAATTCGGGATTCTGGAGCTAAAACAGGAA
priXL446	CGAACAAGTGGAGAACATAGAA
priXL447	ATAAATAATGCGGGCATCACG
priXL448	ATTAGCTACAATCAAGGCTAAAA
priXL457	CAAGCTTGCATGCCTGCAGGTCGACTCTAGAGGATCCGGTTATTAAACTGAGTTGGATT
priXL460	TGACCCCGAAGCAGGGTTATGCAGCGGAAAAATTCGGGTCAACAGGAATACGTGTAAAC
priXL461	CCAAGCTTGCATGCCTGCAGGTCGACTCTAGAGGATCCCCGTTTTCATCAAAAACAACC
priXL462	GTTTTTTTTATTCTATTTATTTCGCTCCTATTGTTAATTTTTTAGGTGCGC
priXL463	TGCGCACCTAAAAAATTAACAATAGGAGCGAAATAAATAGAATAAAAAAAAC
priXL464	TGACCCCGAAGCAGGGTTATGCAGCGGAAAAATTCGGGAAGATACTCGTTCTGGTTTTG
priXL465	CAAGCTTGCATGCCTGCAGGTCGACTCTAGAGGATCCCAATATTAAAACCGCGCTCTA
priXL466	AAAATGAAAAATAAACTAGGATGTTCAAACGATCCAGAATCA
priXL467	GATTCTGGATCGTTTGAACATCCTAGTTTATTTTTCATTTTCGAG
priXL468	CCCCGAAGCAGGGTTATGCAGCGGAAAAATTCGGGGATGATGATGCTTTTCATAAATCT
priXL469	TTGTTATAGCATCGCCAAATG
priXL470	TCATCGTATAACATAACGCCG
priXL471	TGGTCTTGTAATAACGAACG
priXL472	CCAGCTGGTATCAAATTCAAT
priXL473	ATGCGTGATGTAATTTGAATC
priXL474	TTATATTGTGTGGATGATGCC
priXL475	CATCCAAAAAACAGTGTCACA

priXL476	ATGAGATTGACGGTTTTATCG
priXL484	ATTTATTCATTTTGACAAAGAGTTGTTCAAACGATCCAGAATCA
priXL485	TGATTCTGGATCGTTTGAACAACTCTTTGTCAAAATGAATAAATTAGAA
priXL486	CCCCGAAGCAGGGTTATGCAGCGGAAAAATTCGGGAGTTCAACACTTAATAGGCTTA
priXL489	CCAAGCTTGCATGCCTGCAGGTCGACTCTAGAGGATCCATTGATATTGGATGTGCTGGT
priXL490	TGCTTTAAATCAGGATATGGTACTAAAAAGGAATTAAAACTCTACT
priXL491	AGAGTTTTAATTCCTTTTTAGTACCATATCCTGATTTAAAGCACT
priXL492	ACCCCGAAGCAGGGTTATGCAGCGGAAAAATTCGGGCGTAAAATTTCTTCTATCGAGAG
priXL493	TGTGTCTCTCTTGAAATTACTA
priXL494	AGTTTCCTTAGATGCTGAAAAC
priXL495	AATTTATCGTCTATACTGCTTTT
priXL496	CAAGCTTGCATGCCTGCAGGTCGACTCTAGAGGATCCGCAGGAGTTACTAACATCATCA
priXL497	TAGGGATACAAGCAAAAGTAACACCAAATTTTTTAAATACAAAATCAAATCCCTATCAGTGAT AGAGAAAAAACCTACAAAAAAACATGCGTTGTATAACGCATTAC
priXL498	ATTTAAAAAATTTGGTGTTACTTTTGCTTGTATCCCTATCAGTGATAGAGAATTAACTAAATTG TAATTAAAAAAATTAAGATGATTAAAAAAATTATAATAAGTGCT
priXL499	TCTGTTAATCTACGTCTTAATTCCTCTTCTAATCTAGAGTTGAATTTGCTCTTCACAAA
priXL500	ATTAGAAGAGGAATTAAGACGTAGATTAACAGAATAATCAATGTTCTGATGGTGTTAAC
priXL501	GACCCCGAAGCAGGGTTATGCAGCGGAAAAATTCGGGAACTTGAAGCAGAATTTGAAG
priXL504	ATAGGGAACGTCGCAAAATTACAAAATTTTATGCAGTTTATAACTAATATATTATTCCCTATCA GTGATAGAGACTAAATTTTAATATGCGTTGTATAACGCATTAC
priXL506	AAATTTTTTAAATACAAAATCAAATGTTAAAAAAAACCTACAAAAAAACATAATACAACCAATG CGTTTGACGG
priXL507	TTAGAAAAACAACTTAAATGTGAAAGTGGGTCTTAATTGTAGTTGTGAGTTTCAAATAC
priXL508	TTAAGACCCACTTTCACATTTAAG
priXL509	TTTTTTTAACATTTGATTTTGTATTTAAAAAATTTGGTGTTACTTTTGCTTGTAATTAACTAAAT TGTAATTAAAAAAATTAAGTATGTCTAGATTAGATAAAAGTAAAGTGATT
priXL510	CTTGGGATAGGTTTTCCTTCAAATTCTGCTTCAAGTTCCCGAATTTTTCCGCTGCAT

priXL511	TCGTTATTTAATACATTGATGATGTTAGTAACTCCTGCGGATCCTCTAGAGTCGACCT
priXL512	GCAGGAGTTACTAACATCATCAA
priXL513	AACTTGAAGCAGAATTTGAAGG
priXL515	CATCTTAATTTTTTTAATTACAATTTAGTTAATTCTCTATCA
priXL522	AAATACTCGAAGAACGAGAAG
priXL523	AATTAATACACGTTTGTGTGTTG
priXL536	TCAATGTTCTGATGGTGTTAACTG
priXL537	CATAATAGTATTTTACTTAATTTTTGGTAATAAAATTCTC
priXL538	AGAATTAACTAAATTGTAATTAAAAAAATTAAGATGGTCTTCACACTCGAAGATTTC
priXL539	TTTTATTACCAAAAATTAAGTAAAATACTATTATGGTCTTCACACTCGAAGATTTC
priXL540	CTTCAGTGCAGTTAACACCATCAGAACATTGATTACGCCAGAATGCGTTCGC
priXL543	GATAAATGGTTTGTGAAGAGCA
priXL544	GAACGATATAAGCAAACAAGCA
priXL545	ATGATTAAAAAAATTATAATAAGTGCTTGTTT
priXL546	TTAGTTGAATTTGCTCTTCACA
priXL551	TCTAGATTAGAAGAGGAATTAAGACGTAGATTAACAGAAGATCCGGACCCTTATGCATA
priXL552	CTGTTAATCTACGTCTTAATTCCTCTTCTAATCTAGAGCCGCTTACATTAATTTCTCG
priXL557	AGAATTAACTAAATTGTAATTAAAAAAATTAAGATGTTACAAAAAAGAATACCACAAAT
priXL558	TCTTCAGTGCAGTTAACACCATCAGAACATTGACTAAAATTGTTGTCCTATGATAAAGT
priXL560	TAACTAAATTGTAATTAAAAAAATTAAGATGTACAATACTTCTTTTATTAAGAAAATTC
priXL561	TTCTTCAGTGCAGTTAACACCATCAGAACATTGATTAATTTGGTTTCGTGTAGTAAACC
priXL562	ATTACCAAAAATTAAGTAAAATACTATTATGTACAATACTTCTTTTATTAAGAAAATTC
priXL563	CAAGCTTGCATGCCTGCAGGTCGACTCTAGAGGATCCCTTTTCTCGTTCCAACTATACG
priXL564	TTTGCTTTATTAGTAGTAGAGGTACAGATTATTTGTGGTATTCT
priXL565	ACAAATAATCTGTACCTCTACTACTAATAAAGCAAATGGC
priXL566	AAGCAGGGTTATGCAGCGGAAAAATTCGGGAACGCCTATAGGATTATTGTTTG
priXL567	GTGCAGTTAACACCATCAGAACATTGATTAGTTGAATTTGCTCTTCACA
priXL568	CTTGTTTGCTTATATCGTTCGAAGATGTAGGGATGACTTTAG

priXL569	CTAAAGTCATCCCTACATCTTCGAACGATATAAGCAAACAAG
priXL570	ACAACCAATCCTAAGTATTGTACATATTAAATAATCAAAATT
priXL571	ATTATTTAATATGTACAATACTTAGGATTGGTTGTTACAGAAAG
priXL580	GAAAAGGTATAGACTGGCCAG
priXL581	AAGCAAATCCTAATGTTTGTTTG
priXL634	ATGCCTGCAGGTCGACTCTAGAGGATCCTTAGCTTTAGTTGACACTTATAC
priXL635	ACTTCAGTGATATCCATAGCTTAGAATTTTATTGTTAAGT
priXL636	TAAAATTCTAAGCTATGGATATCACTGAAGTTCCTAAAC
priXL637	AGCAGGGTTATGCAGCGGAAAAATTCGGGCTTTCTTCGAATTAGATTGCG
priXL644	ATTTATATTTTTAGCCTTGATTGTAG
priXL645	GGAAATTACTTATTCTATGTTCTCC
priXL646	GTTCTAGATACGGAAAGCAGATTGGAGCAA
priXL647	TTGCTCCAATCTGCTTTCCGTATCTAGAACT
priXL648	CAAATCCAGCAGTATTAAAATC
priXL649	CGCTTTTTGATAGACTAAATCC
priXL650	TACCCATACGATGTTCCAGATTACGCTGATAGAGTTCCTTTTGATCAAGG
priXL651	AGCGTAATCTGGAACATCGTATGGGTATTGAGCTTTTATTTGTGAAAAACTAC
priXL656	GCATGCCTGCAGGTCGACTCTAGAGGATCCGTATCTTGTTGTTATGGGAAC
priXL657	TTAGCATAAGCTGTAAGGGCAGCAACAATTAATAG
priXL658	TTGTTGCTGCCCTTACAGCTTATGCTAATTTGATGAAA
priXL659	GAAGCAGGGTTATGCAGCGGAAAAATTCGGGGCAATTTACTCTCTAAATCAGC
priXL747	ATGTGACCACGCCGAACCTCC
	GGTGGATCGGGAGGTTCGGCGTGGTCACATCCACAATTTGAGAAGGATGTTGACTCATCT
priXL748	ACCCCG
priXL749	AATTGGAGGTTTCTATTGCTGAATTCCCGGTTAATTCCTTTTTAGTACTTTATATTGTT
	GGTGGATCGGGAGGTTCGGCGTGGTCACATCCACAATTTGAGAAGAACGGTACAAGAAA
priXL750	GTCGCTG
priXL751	AATTGGAGGTTTCTATTGCTGAATTCCCGGTTATTGAGCGGCATTCACTTTTTT

oLO130	TCTGGCGGTGGATCGGATAAAGATTTTAATGCGATTGGCG
oLO131	AGCCGGATCTCATCATTAATTTGGTTTCGTGTAGTAAACCT
oLO132	CGATCCACCGCCAGA
oLO133	TGATGAGATCCGGCTGCTAA
oLO134	TCTGGCGGTGGATCGAATAAAAAGGATGATGATGAAGAGATTG
oLO135	AGCCGGATCTCATCATTAATTCTTTTTATAAGGATATGTATTATCTAAATGAATTC
oLO178	TCTGGCGGTGGATCGCAAAACGGTACAAGAAAGTCG
oLO179	AGCCGGATCTCATCATTATTGAGCGGCATTCACTT
oLO180	TCTGGCGGTGGATCGCAAGAAAGAGCTGTTTCTGGA
oLO184	TCTGGCGGTGGATCGGATTCAGCAGATGACAATGTAAATTT
oLO185	AGCCGGATCTCATCATTAGATTGCGTCAGCATCAAA
oLO186	CCTCTCCCCGCGCGAGAAAAGCAAACACTTTTGATGG
oLO187	GTGGCTCCAAGCGCTCGCAAAAGAAAGTTGCGC
oLO188	CAACTTTCTTTTGCGAGCGCTTGGAGCCAC
oLO189	AACAGCTCTTTCTTGACTAGTTTTCTCGAACTGCG
oLO190	TTCGAGAAAACTAGTCAAGAAAGAGCTGTTTCTGGA
oLO191	CGCCGTTGGATACACCAAGGTGAAACTTTTGCTGTTGCAGA
oLO205	AGCCGGATCTCATCATTATCCAGCTAATTTTACGTTAACTGT
oLO207	TCCACCAATCTGTTCTCTGT
oLO208	GGCTGCTAACAAAGCCC
oLO209	GGGCTTTGTTAGCAGCCTTAGAATTTATCCCAGAAAAGTTTAGTAGT
oLO210	GAACAGATTGGTGGATCTGATGATTTTGGGAATATGAACC

Supplementary references

67. Blattner, F. R. *et al.* The complete genome sequence of *Escherichia coli* K-12. *Science* 277, 1453–1462 (1997).

68. Braun, T. F. & McBride, M. J. *Flavobacterium johnsoniae* GldJ is a lipoprotein that is required for gliding motility. *J. Bacteriol.* 187, 2628–2637 (2005).
