# Supplementary material for: A new paradigm for outer membrane protein biogenesis in the Bacteroidota
Source: Nature. 2025 Oct 1;647(8089):479–87. doi: 10.1038/s41586-025-09532-8 (PMC12611786; doi:10.1038/s41586-025-09532-8)
Supplement: Supplementary file 2 — Reporting Summary [file 41586_2025_9532_MOESM2_ESM.pdf]

# nature portfolio

Corresponding author(s): Ben C. Berks, Susan M. Lea, and Xiaolong Liu

Last updated by author(s): 8th July 2025

# Reporting Summary

Nature Portfolio wishes to improve the reproducibility of the work that we publish. This form provides structure for consistency and transparency in reporting. For further information on Nature Portfolio policies, see our Editorial Policies and the Editorial Policy Checklist.

## Statistics

For all statistical analyses, confirm that the following items are present in the figure legend, table legend, main text, or Methods section.

| n/a | Confirmed | |
|---|---|---|
| ☐ | ☒ | The exact sample size (*n*) for each experimental group/condition, given as a discrete number and unit of measurement |
| ☐ | ☒ | A statement on whether measurements were taken from distinct samples or whether the same sample was measured repeatedly |
| ☐ | ☒ | The statistical test(s) used AND whether they are one- or two-sided<br>*Only common tests should be described solely by name; describe more complex techniques in the Methods section.* |
| ☐ | ☒ | A description of all covariates tested |
| ☐ | ☒ | A description of any assumptions or corrections, such as tests of normality and adjustment for multiple comparisons |
| ☐ | ☒ | A full description of the statistical parameters including central tendency (e.g. means) or other basic estimates (e.g. regression coefficient) AND variation (e.g. standard deviation) or associated estimates of uncertainty (e.g. confidence intervals) |
| ☐ | ☒ | For null hypothesis testing, the test statistic (e.g. *F*, *t*, *r*) with confidence intervals, effect sizes, degrees of freedom and *P* value noted<br>*Give P values as exact values whenever suitable.* |
| ☒ | ☐ | For Bayesian analysis, information on the choice of priors and Markov chain Monte Carlo settings |
| ☒ | ☐ | For hierarchical and complex designs, identification of the appropriate level for tests and full reporting of outcomes |
| ☒ | ☐ | Estimates of effect sizes (e.g. Cohen's *d*, Pearson's *r*), indicating how they were calculated |

*Our web collection on statistics for biologists contains articles on many of the points above.*

## Software and code

Policy information about availability of computer code

Data collection

Data analysis

SIMPLE 3.0
RELION 4.03
PHENIX 1.21
COOT 0.9
MolProbity 4.5.2
UCSF ChimeraX-1.9
cryoSPARC v4.5.3
UCSF pyem 0.5
Perseus 2.1.3.0
MaxQuant 2.5.1.0
PerseusR 0.3.4
PDBeFold 2.58
SignalP 6.0

For manuscripts utilizing custom algorithms or software that are central to the research but not yet described in published literature, software must be made available to editors and reviewers. We strongly encourage code deposition in a community repository (e.g. GitHub). See the Nature Portfolio guidelines for submitting code & software for further information.

## Data

Policy information about availability of data

All manuscripts must include a data availability statement. This statement should provide the following information, where applicable:

- Accession codes, unique identifiers, or web links for publicly available datasets
- A description of any restrictions on data availability
- For clinical datasets or third party data, please ensure that the statement adheres to our policy

Electron Microscopy DataBank (EMDB): EMD-48835 (BAMFj composite map), EMD-48832 (BAMFj consensus map), EMD-48833 (BAMFj BamGM-focused map), EMD-48834 (BAMFj BamADP-focused map), EMD-48836 (BamAP complex), EMD-48837 (BamAD complex).
Protein Data Bank (PDB): 9N2D (BAMFj complex), 9N2E (BamAP complex), 9N2F (BamAD complex). The hybrid model of the BAMFJ complex is provided alongside this paper.
Gel and immunoblot source data are published alongside this paper. The mass spectrometry proteomics data have been deposited to the ProteomeXchange Consortium via the PRIDE partner repository with the dataset identifier PXD065907 and are also available alongside the paper.

## Research involving human participants, their data, or biological material

Policy information about studies with human participants or human data. See also policy information about sex, gender (identity/presentation), and sexual orientation and race, ethnicity and racism.

| | |
|---|---|
| Reporting on sex and gender | N/A |
| Reporting on race, ethnicity, or other socially relevant groupings | N/A |
| Population characteristics | N/A |
| Recruitment | N/A |
| Ethics oversight | N/A |

Note that full information on the approval of the study protocol must also be provided in the manuscript.

# Field-specific reporting

Please select the one below that is the best fit for your research. If you are not sure, read the appropriate sections before making your selection.

☒ Life sciences ☐ Behavioural & social sciences ☐ Ecological, evolutionary & environmental sciences

For a reference copy of the document with all sections, see nature.com/documents/nr-reporting-summary-flat.pdf

# Life sciences study design

All studies must disclose on these points even when the disclosure is negative.

| | |
|---|---|
| Sample size | EM sample sizes were determined by available electron microscope time and density of particles on the grids. Pre-determination of sample size was not possible in this study for the proteomics experiments. 3 biological replicates were used to allow statistical analysis. |
| Data exclusions | EM: Particles were excluded from final analysis using pre-established classification methods within the software packages described. Briefly, 2D classification was used to exclude classes that represented contaminations, while 3D classification was used to remove particles that didn't belong to clearly defined structural elements. Details are given in the manuscript.<br>Other data: no data were excluded. |
| Replication | The number of repeats for each experiment are given in the Figure legends and in all cases the data were successfully reproduced or appropriate statistical analysis was performed. |
| Randomization | Not relevant to this work as sample selection was targeted and all samples were treated the same way. |
| Blinding | Single particle selection was automated. For proteomics the MaxQuant analysis was carried out blind. |

# Reporting for specific materials, systems and methods

We require information from authors about some types of materials, experimental systems and methods used in many studies. Here, indicate whether each material, system or method listed is relevant to your study. If you are not sure if a list item applies to your research, read the appropriate section before selecting a response.

## Materials & experimental systems

| n/a | Involved in the study |
|---|---|
| ☐ | ☒ Antibodies |
| ☒ | ☐ Eukaryotic cell lines |
| ☒ | ☐ Palaeontology and archaeology |
| ☒ | ☐ Animals and other organisms |
| ☒ | ☐ Clinical data |
| ☒ | ☐ Dual use research of concern |
| ☒ | ☐ Plants |

## Methods

| n/a | Involved in the study |
|---|---|
| ☒ | ☐ ChIP-seq |
| ☒ | ☐ Flow cytometry |
| ☒ | ☐ MRI-based neuroimaging |

# Antibodies

Antibodies used

anti-Strep-tag (34850 Qiagen), anti-GroEL (G6532 Merck), anti-ALFA-tag (N1582 Synaptic Systems GmbH), anti-His tag (H1029-100UL Merck Life Science UK Limited), anti-mouse IgG peroxidase conjugate (A4416 Merck), and anti-rabbit IgG peroxidase conjugate (31462 Pierce). OmpA(Shibata, Tahara et al. 2023) was provided by Satoshi Shibata (Tottori University) and antiserum against SprF(Kulkarni, Johnston et al. 2019) by Mark McBride (University of Wisconsin-Milwaukee). Antibodies against BAM subunits, Sus proteins, and SkpA were raised by Davids Biotechnologie in rabbits against the purified recombinant proteins.

Validation

Validated by immunoblotting strains expressing the target antigen versus strains not expressing the target antigen. Anti-GroEL cannot be validated in this way but is used only as a a loading control as in multiple previous studies, fractionates to the cytoplasm, has the correct molecular mass, and has been validated by the commercial supplier.

# Plants

Seed stocks

N/A

Novel plant genotypes

N/A

Authentication

N/A
