# Supplementary material for: A new paradigm for outer membrane protein biogenesis in the Bacteroidota
Source: Nature. 2025 Oct 1;647(8089):479–87. doi: 10.1038/s41586-025-09532-8 (PMC12611786; doi:10.1038/s41586-025-09532-8)

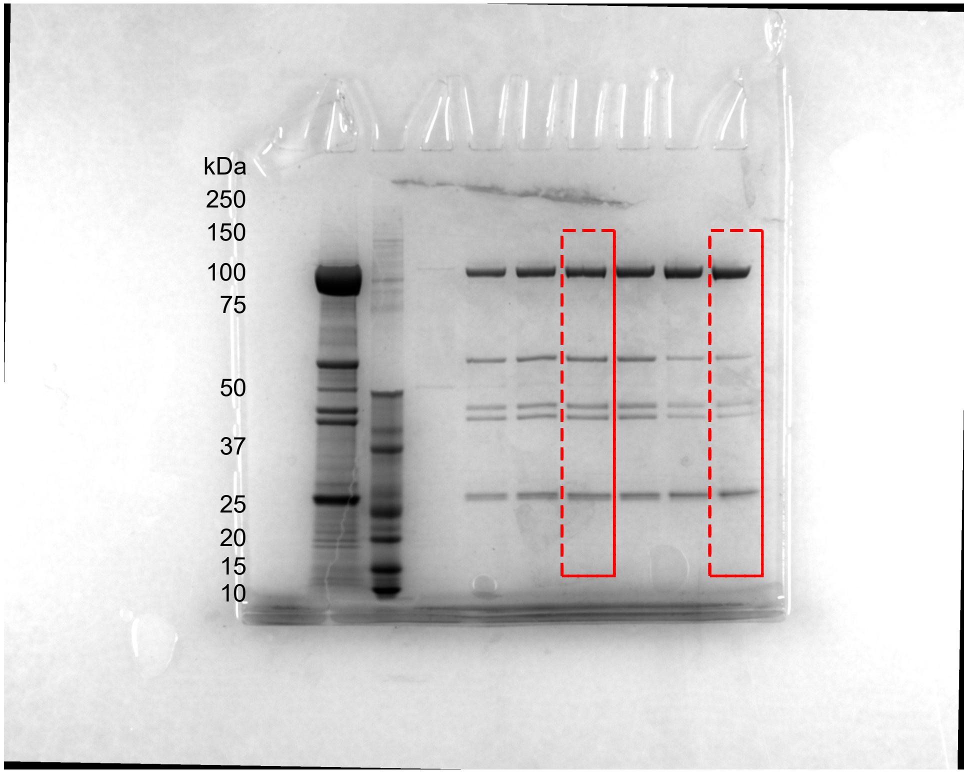

Fig. 1 panel a (Coomassie gel)

Fig. 4 panel b *bamA*^{dep}

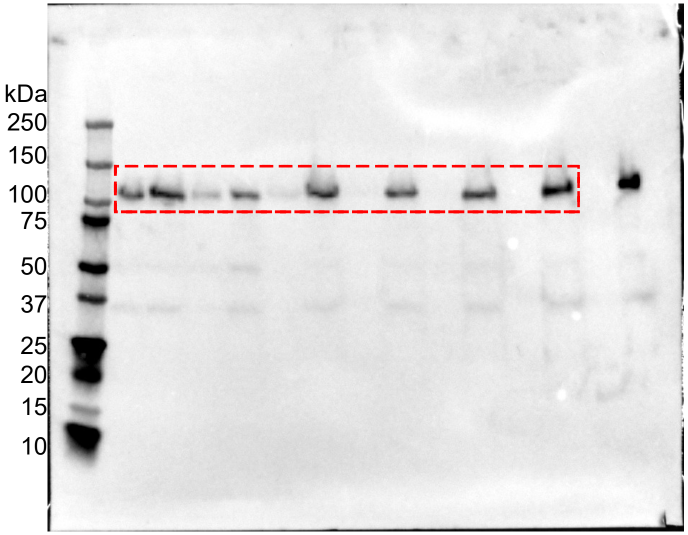

(anti-twinstrep for BamA)

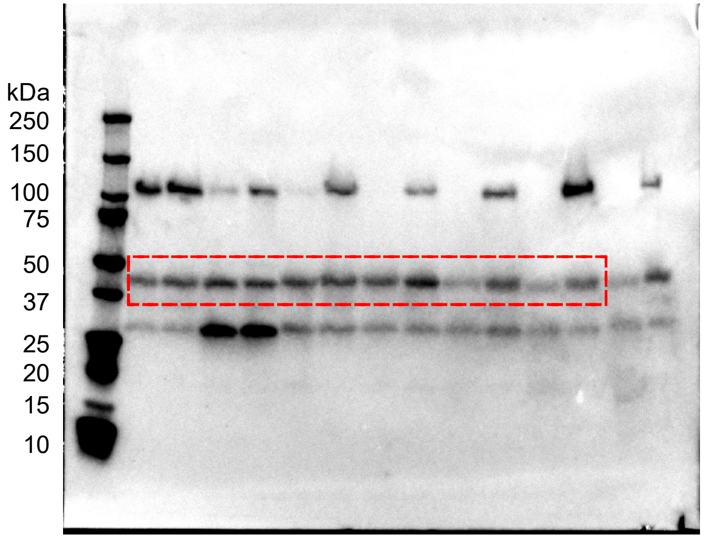

(anti-ALFA for BamG) Reblot the same membrane

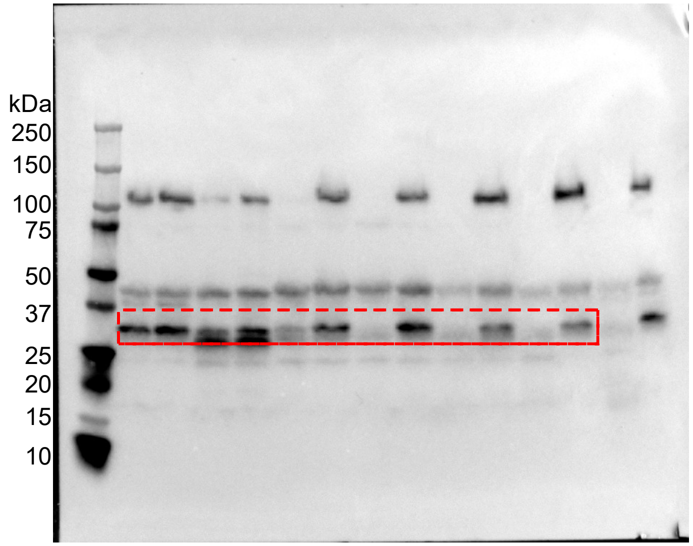

(anti-SprF) Reblot the same membrane

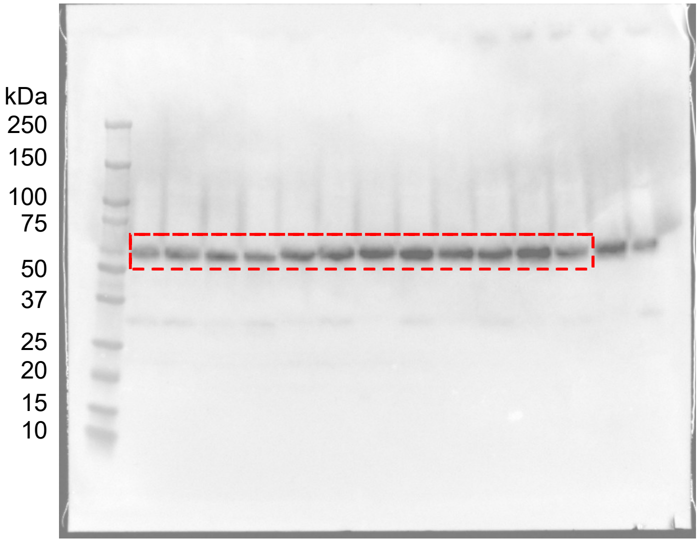

(anti-GroEL) Reblot the same membrane

Fig. 4 panel b *bamA*^{dep}

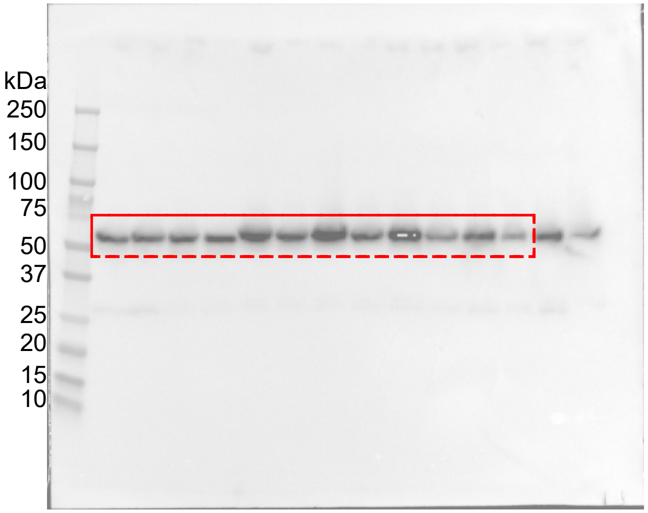

(anti-BamH)

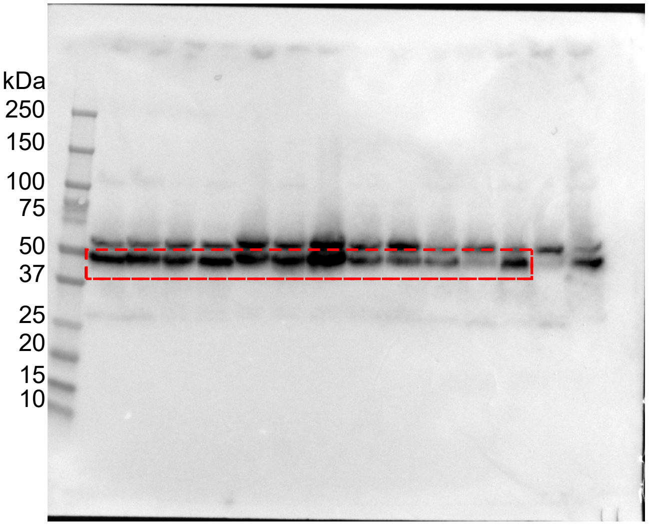

(anti-SkpA)
reblot the same membrane of left

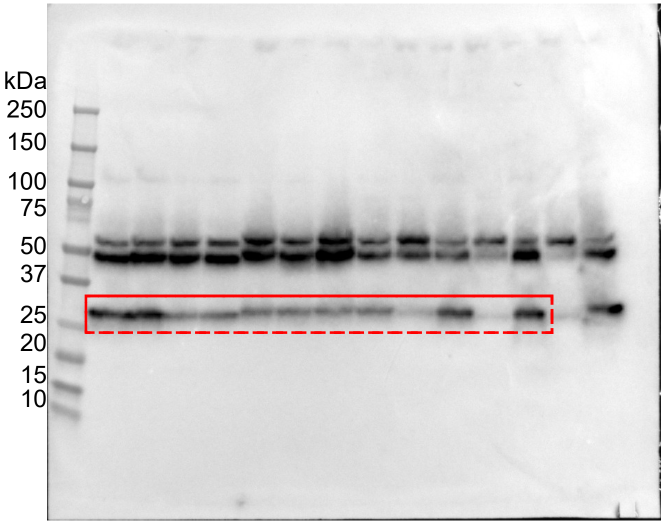

(anti-BamP)
reblot the same membrane of left

Fig. 4 panel b *bamA*^{dep}

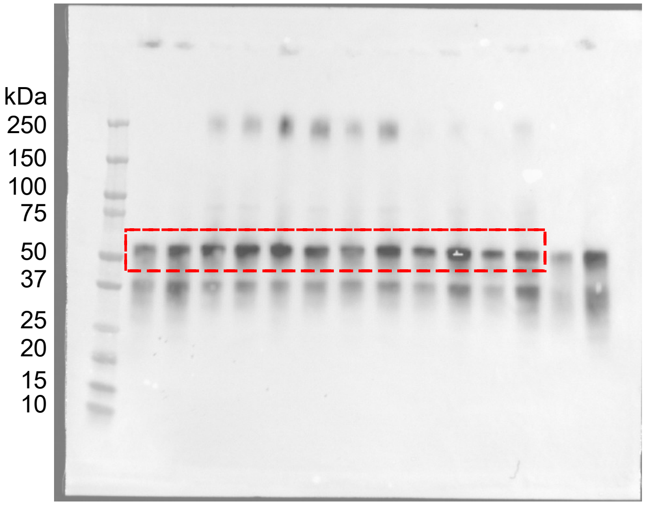

(anti-OmpA)

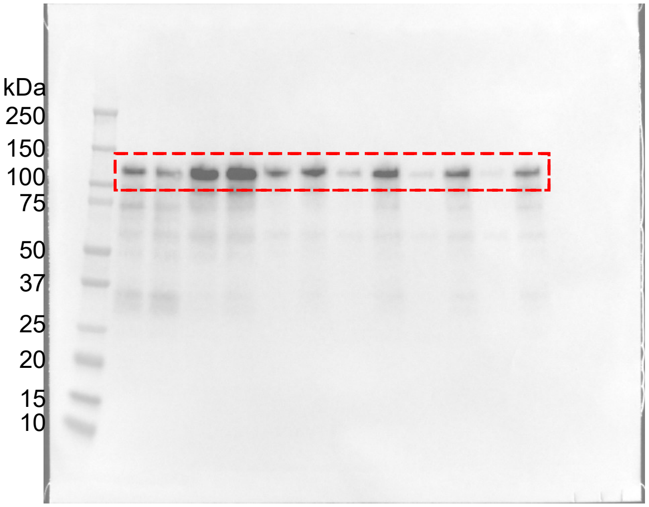

(anti-SusC)

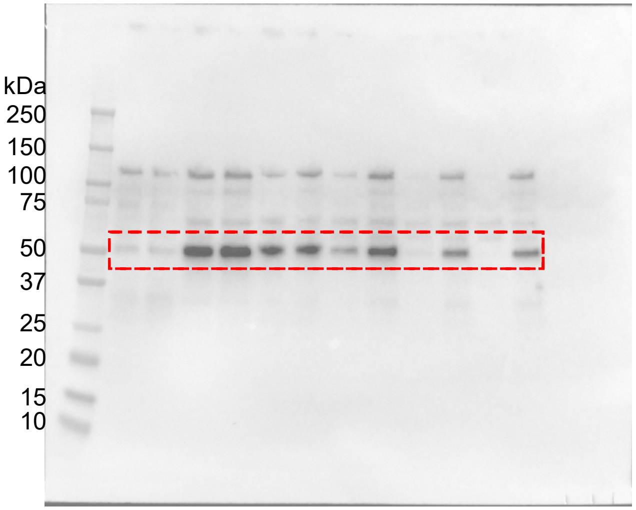

(anti-SusD)
reblot the same membrane of left

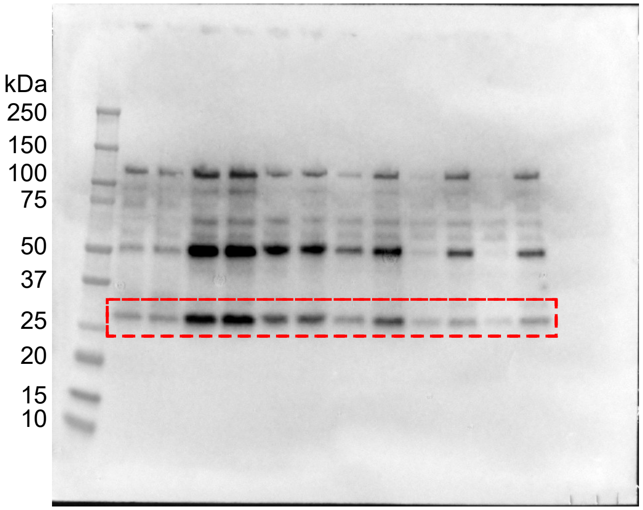

(anti-SusE)
reblot the same membrane of left

Fig. 4 panel b *bamG*^{dep}

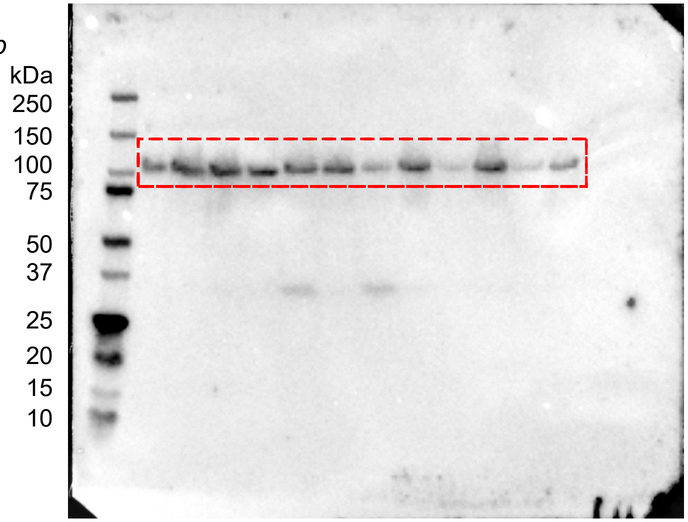

(anti-twinstrep for BamA)

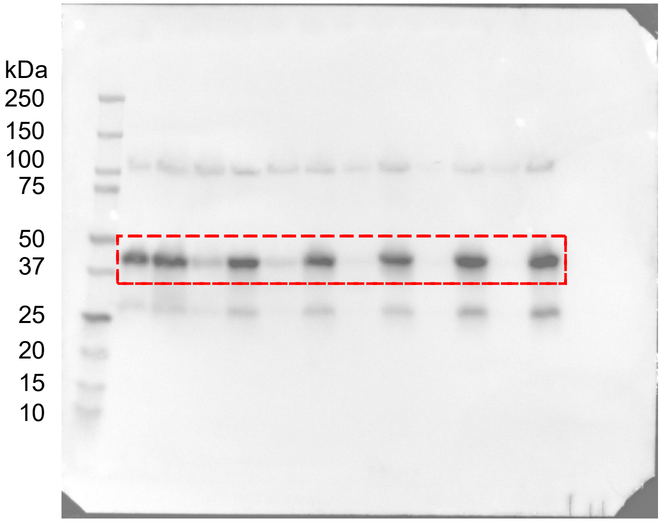

(anti-ALFA for BamG) Reblot the same membrane

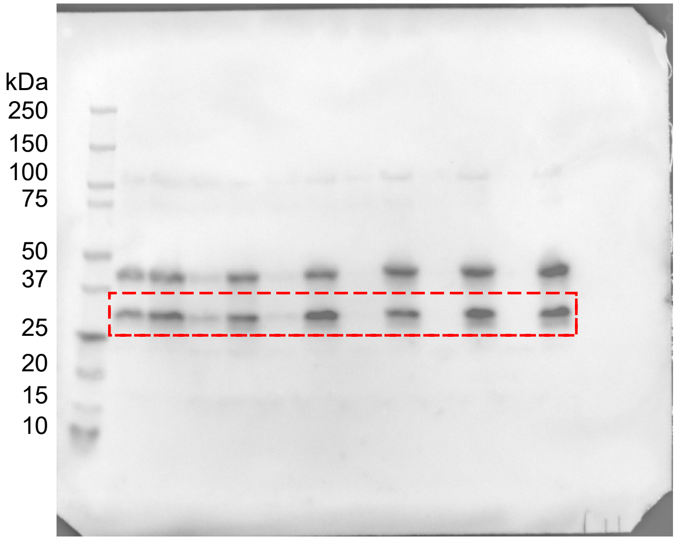

(anti-SprF) Reblot the same membrane

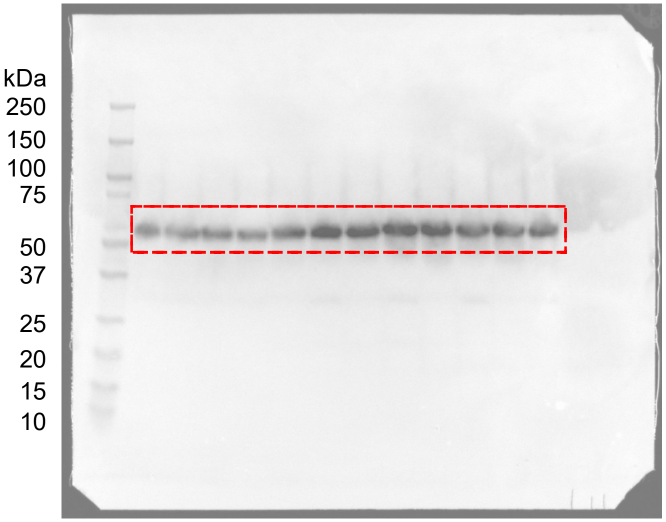

(anti-GroEL) Reblot the same membrane

Fig. 4 panel b *bamG*^{dep}

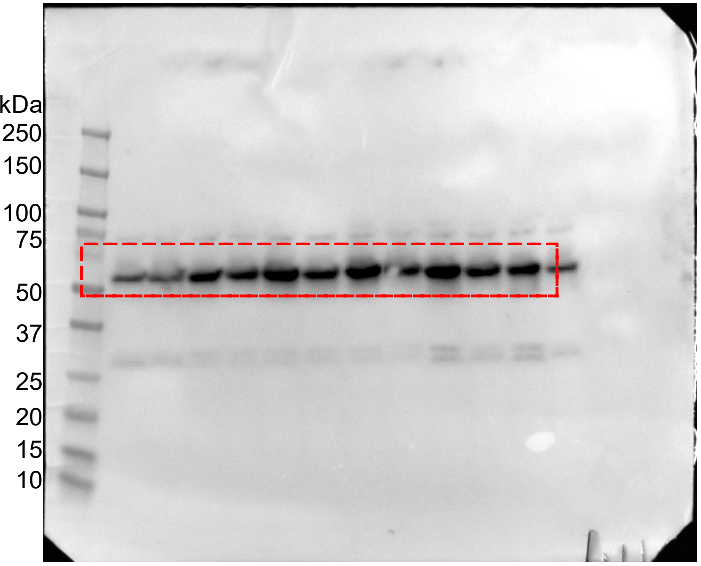

(anti-BamH)

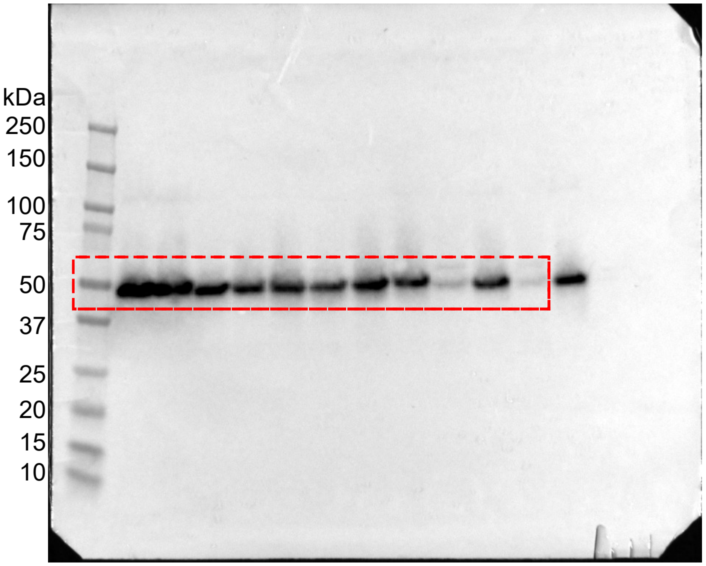

(anti-SkpA)
reblot the same membrane of left

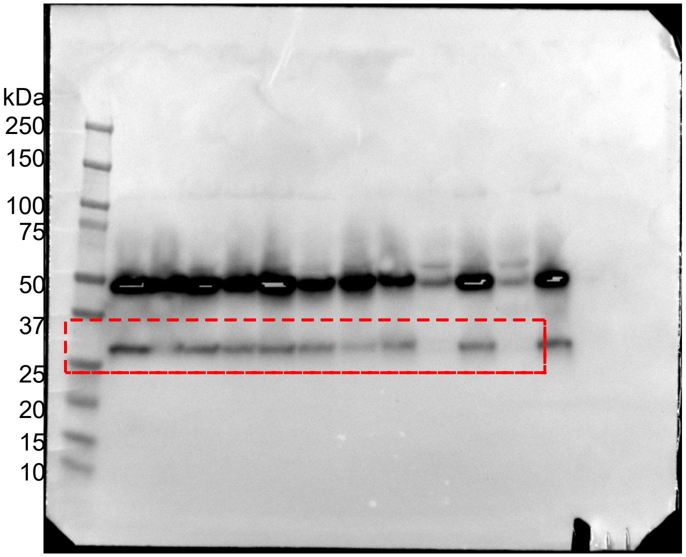

(anti-BamP)
reblot the same membrane of left

Fig. 4 panel b *bamG*^{dep}

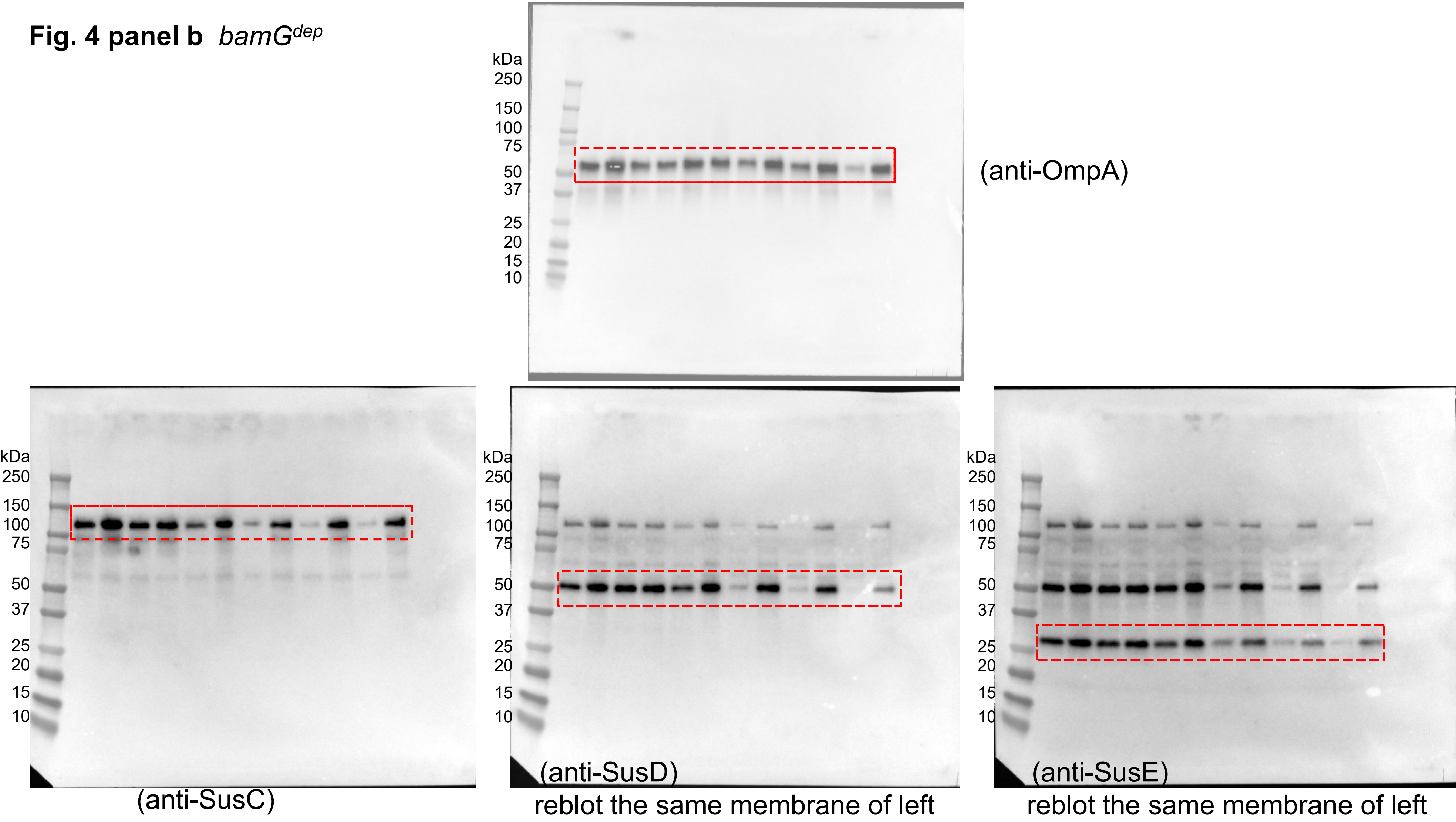

Fig. 4 panel b *bamH*^{dep}

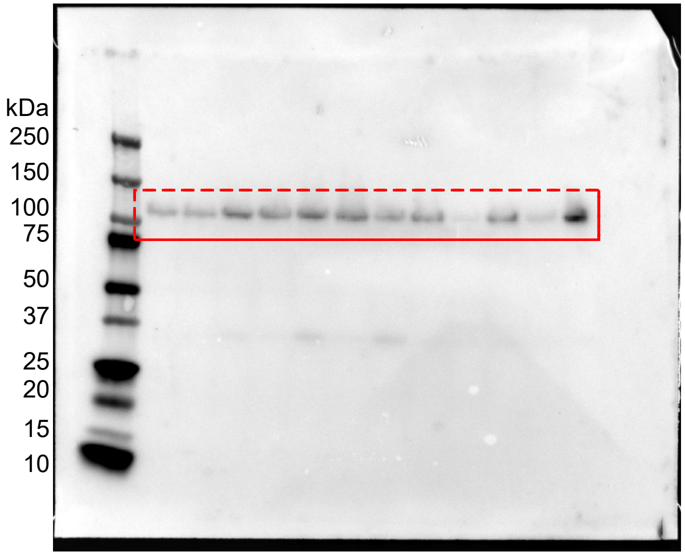

(anti-twinstrep for BamA)

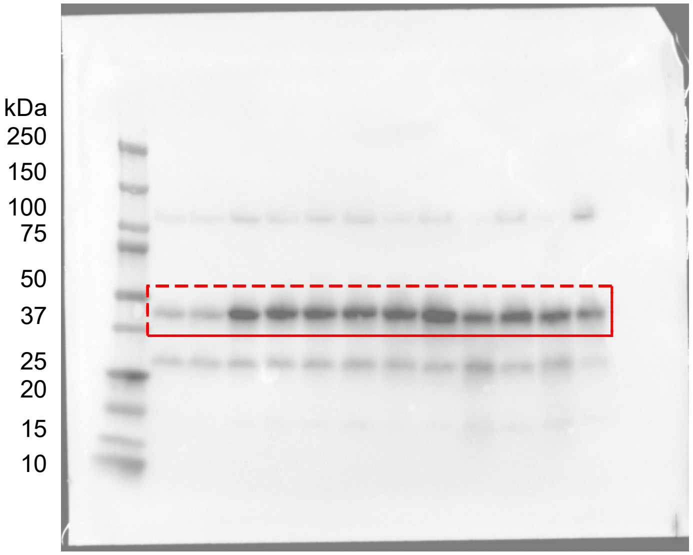

(anti-ALFA for BamG) Reblot the same membrane

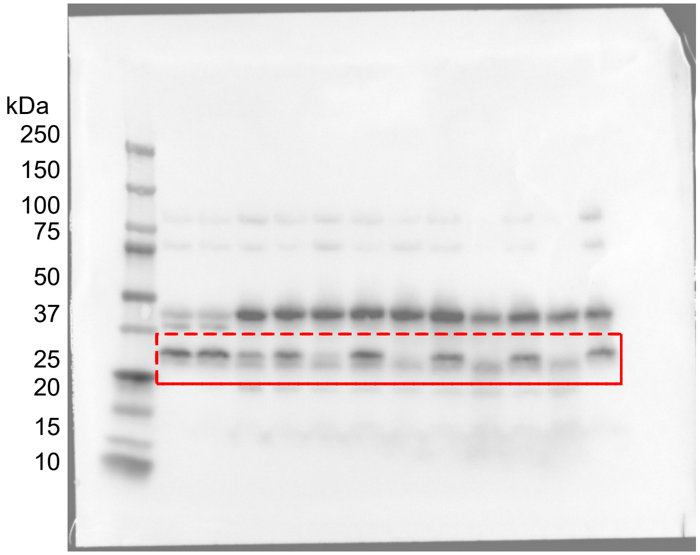

(anti-SprF) Reblot the same membrane

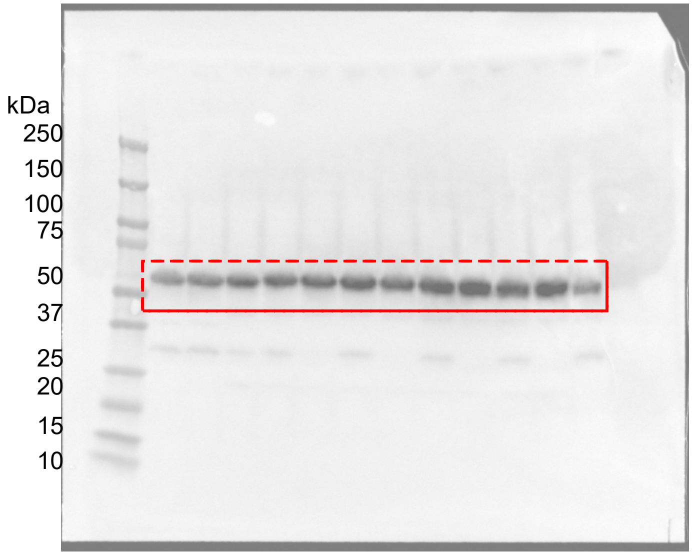

(anti-GroEL) Reblot the same membrane

Fig. 4 panel b *bamH^{dep}*

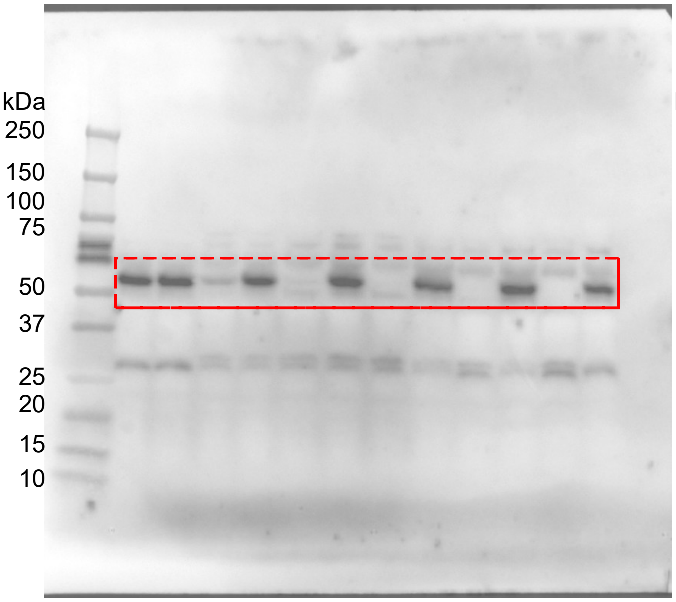

(anti-BamH)

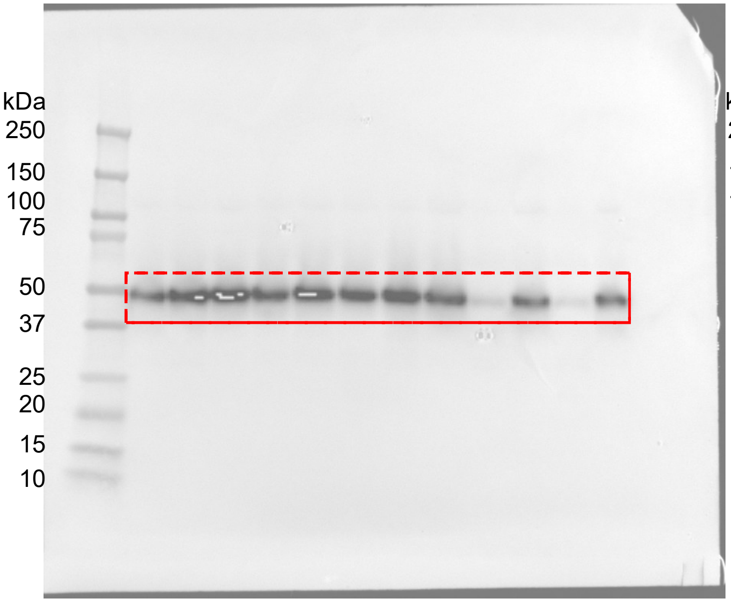

(anti-SkpA)
reblot the same membrane of left

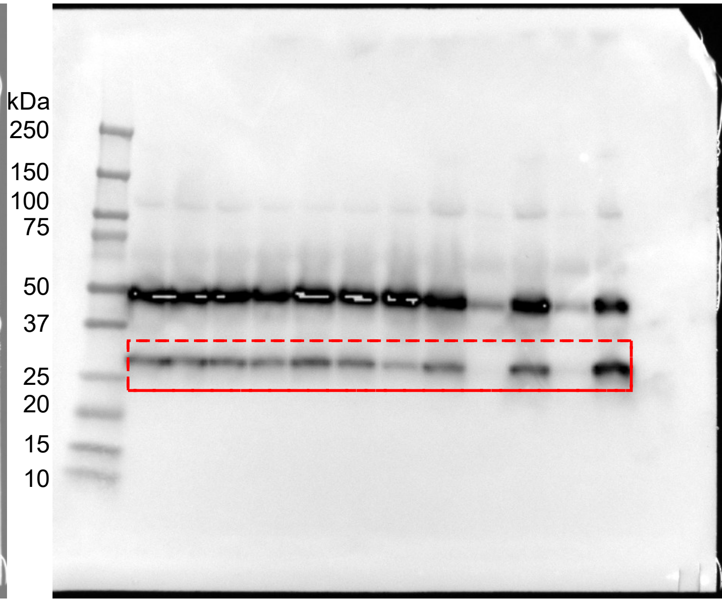

(anti-BamP)
reblot the same membrane of left

Fig. 4 panel b *bamH*^{dep}

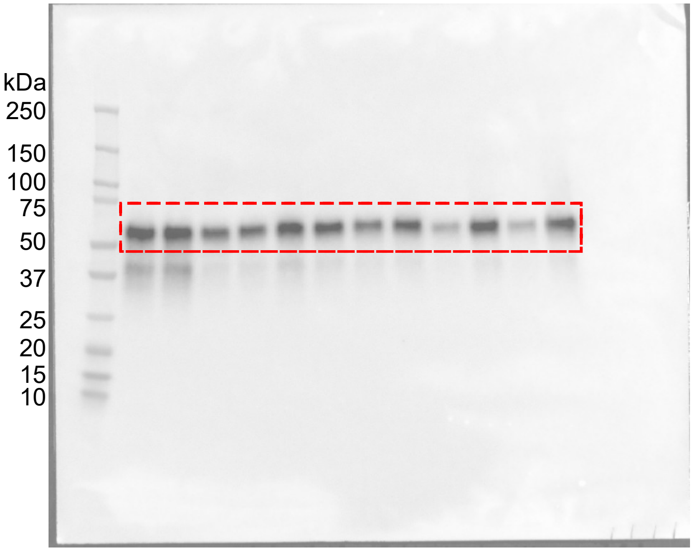

(anti-OmpA)

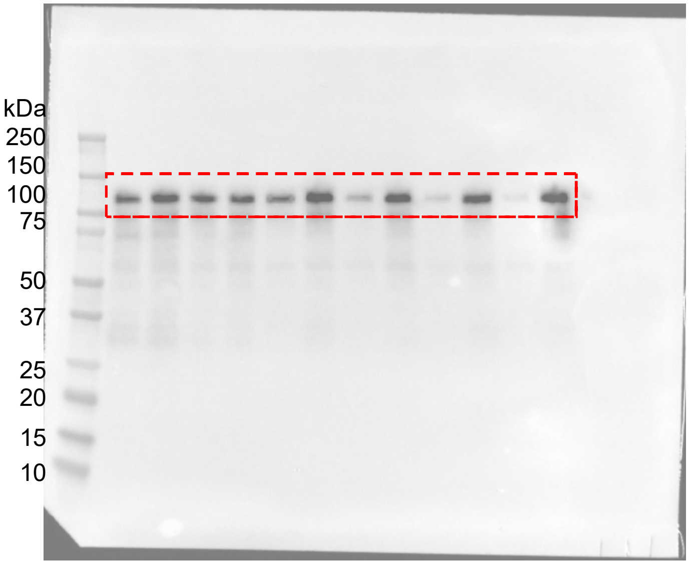

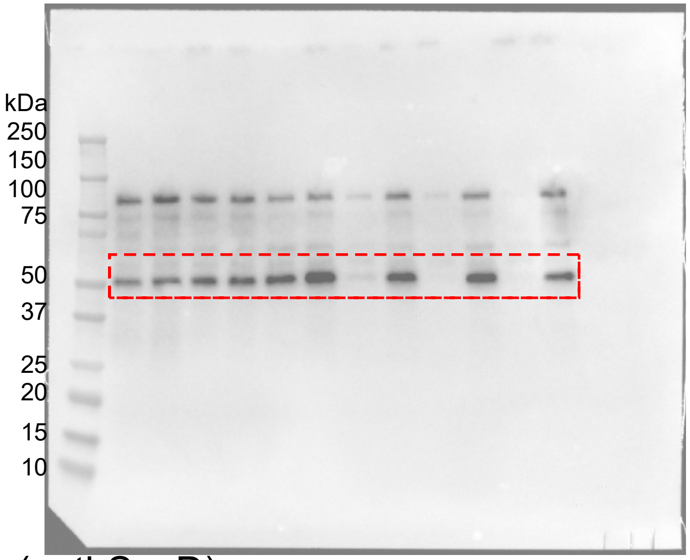

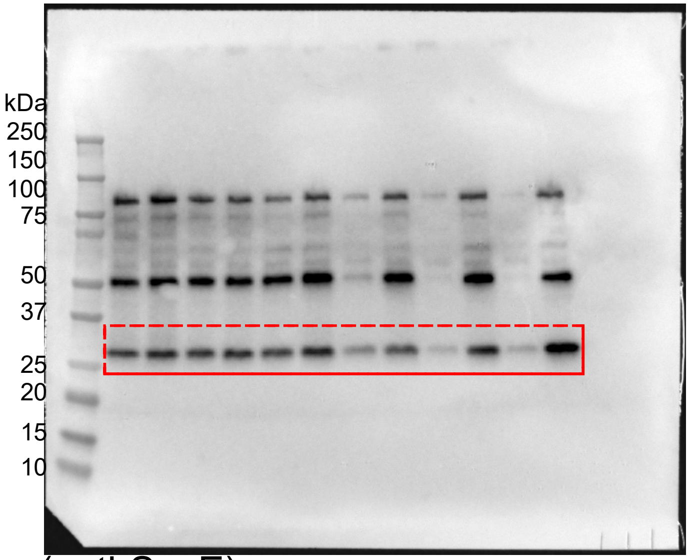

(anti-SusC)

(anti-SusD)
reblot the same membrane of left

(anti-SusE)
reblot the same membrane of left

Fig. 4 panel d

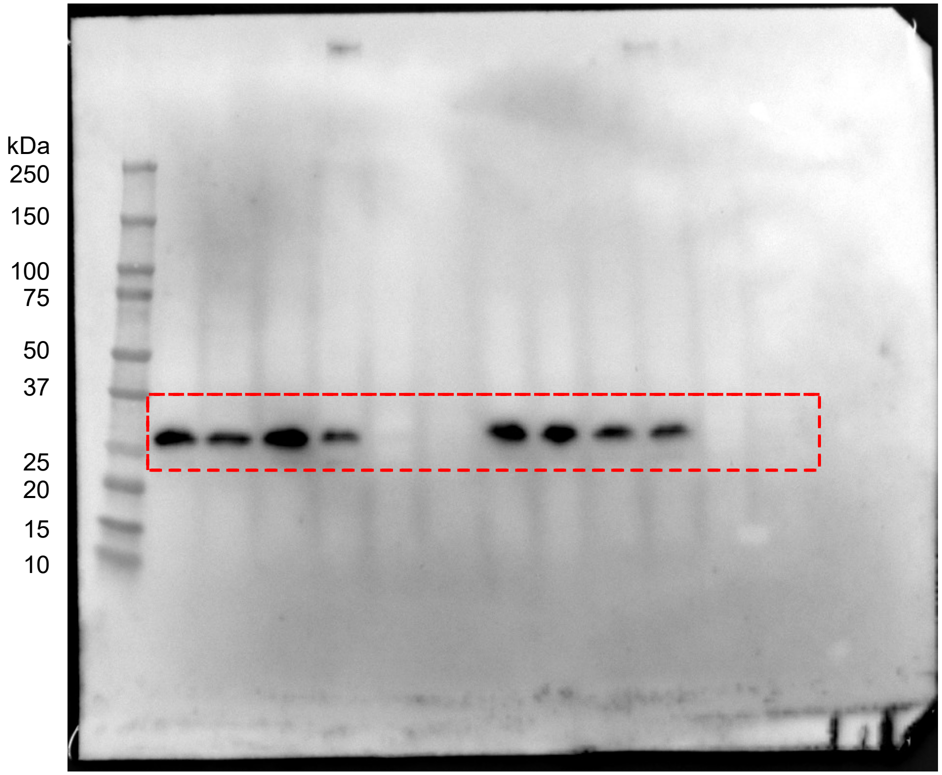

(anti-his for SusE)

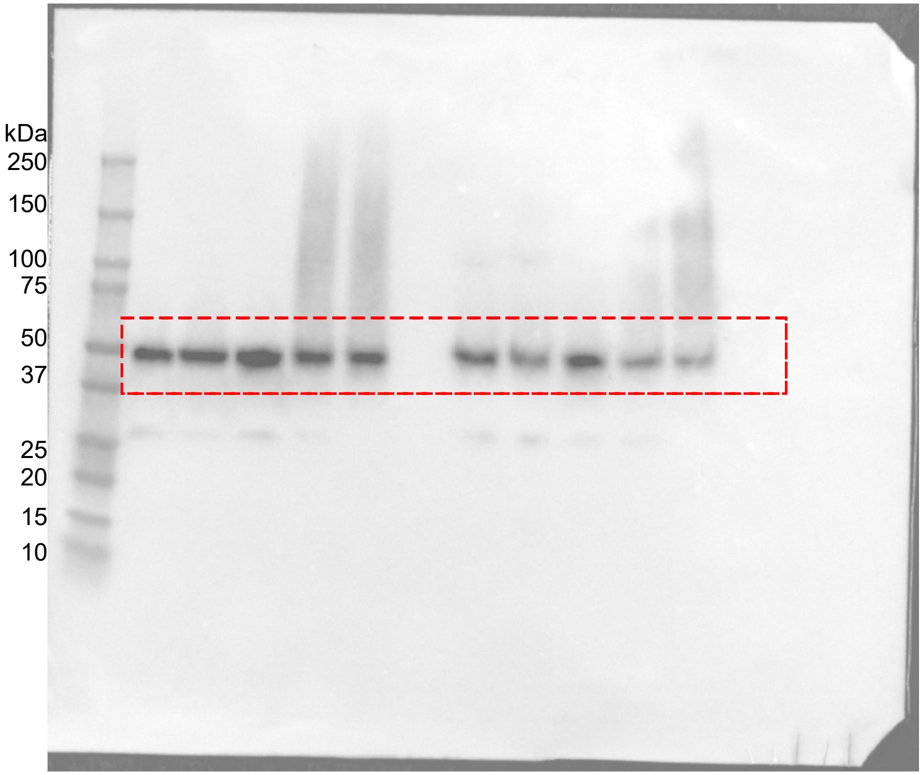

(anti-SkpA) reblot the membrane of left

Fig. 4 panel d

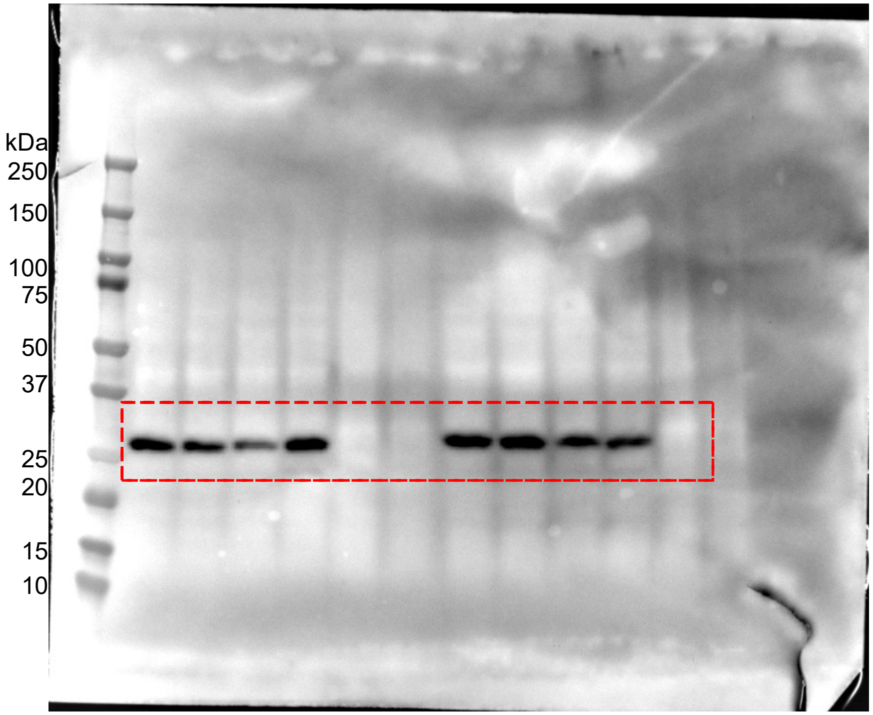

(anti-his for SusE)

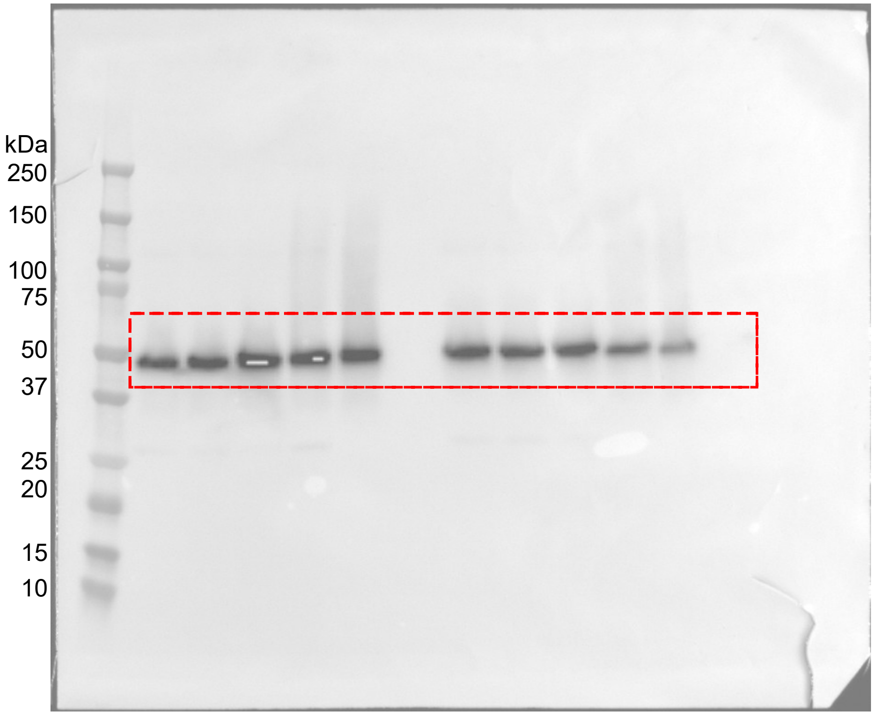

(anti-SkpA) reblot the membrane of left

Fig. 4 panel d

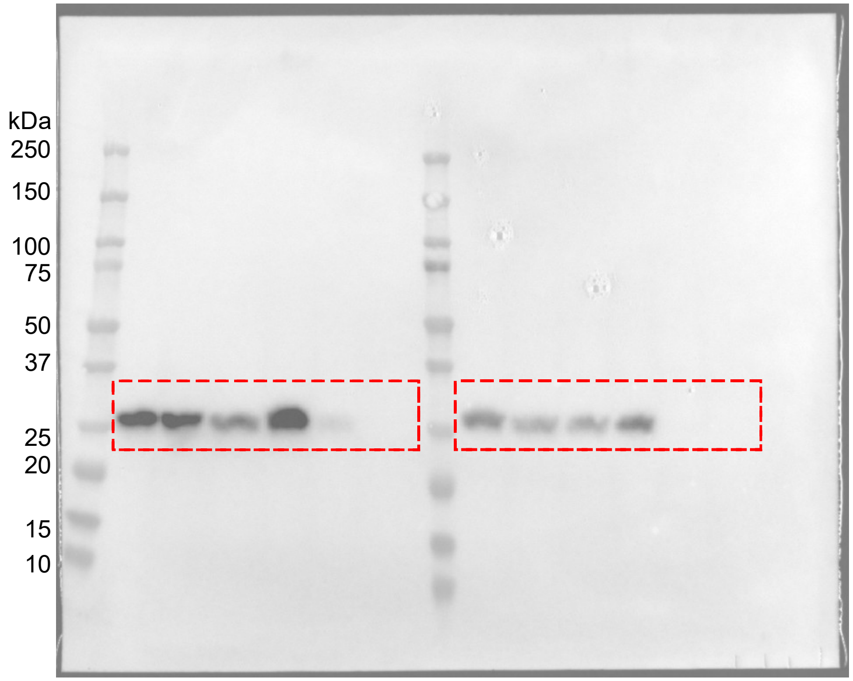

(anti-his for SusE)

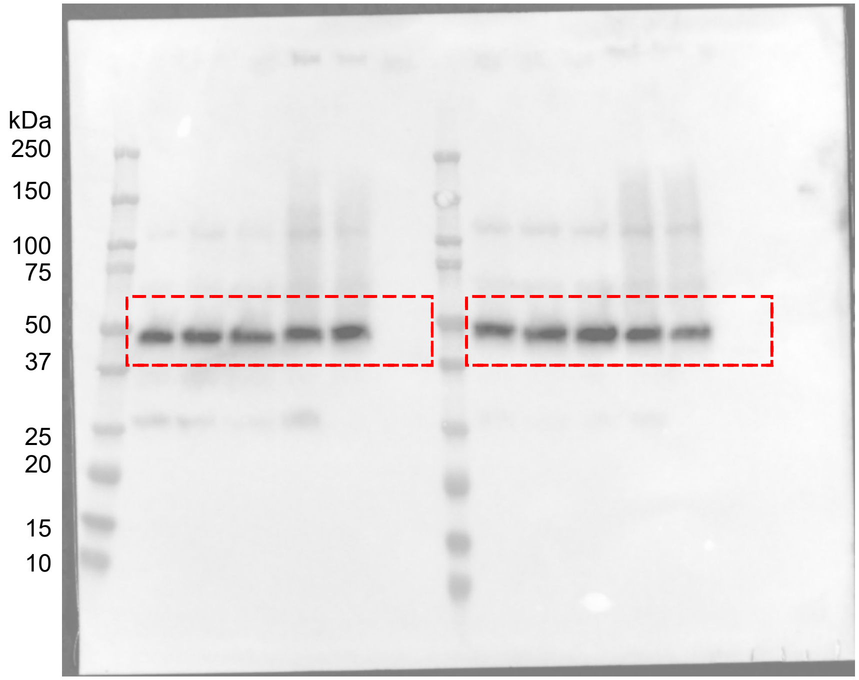

(anti-SkpA) reblot the membrane of left

Fig. 4 panel f

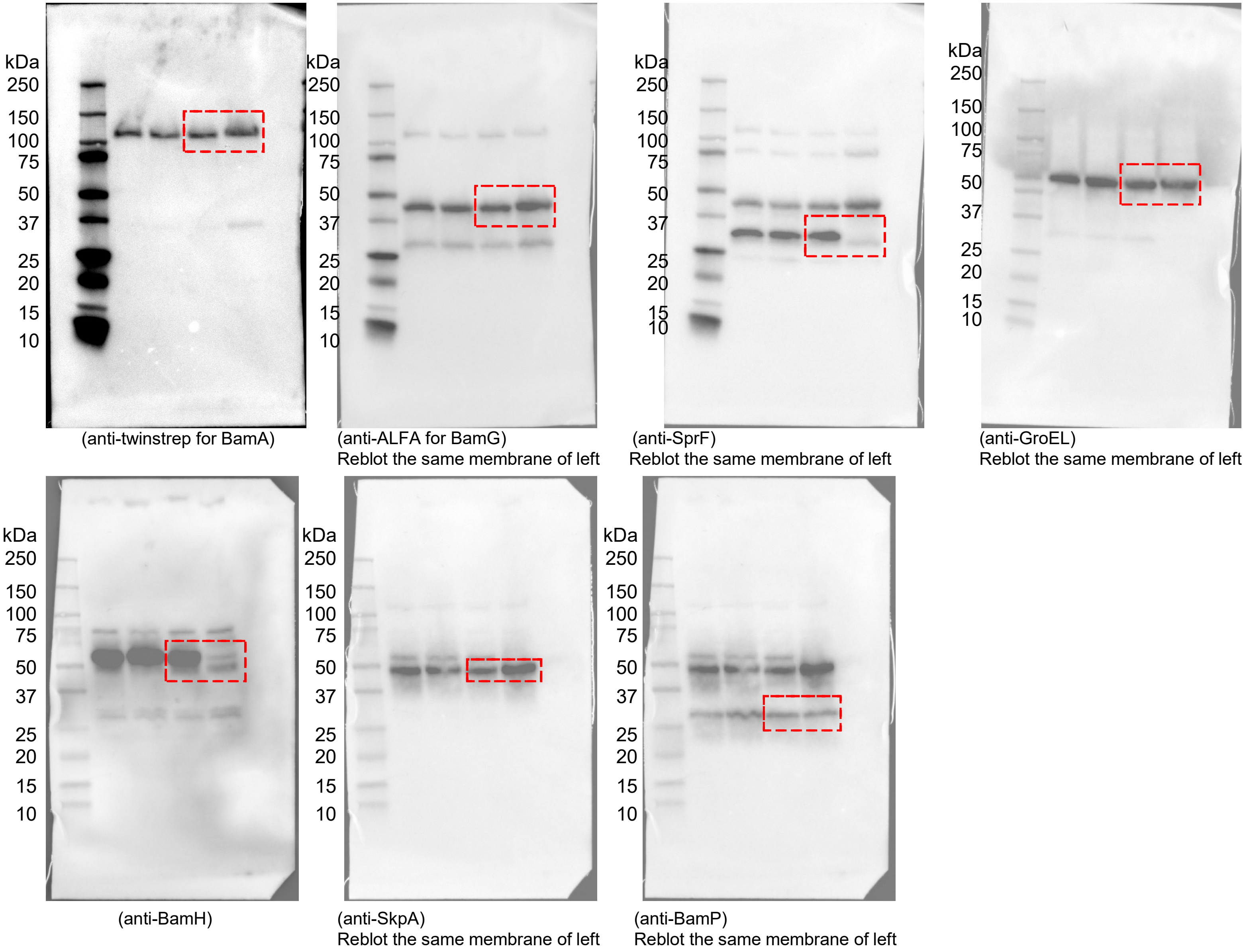
kDa
250
150
100
75
50
37
25
20
15
10
(anti-twinstrep for BamA)
(anti-ALFA for BamG)
Reblot the same membrane of left
(anti-SprF)
Reblot the same membrane of left
(anti-GroEL)
Reblot the same membrane of left
(anti-BamH)
(anti-SkpA)
Reblot the same membrane of left
(anti-BamP)
Reblot the same membrane of left

Fig. 4 panel f

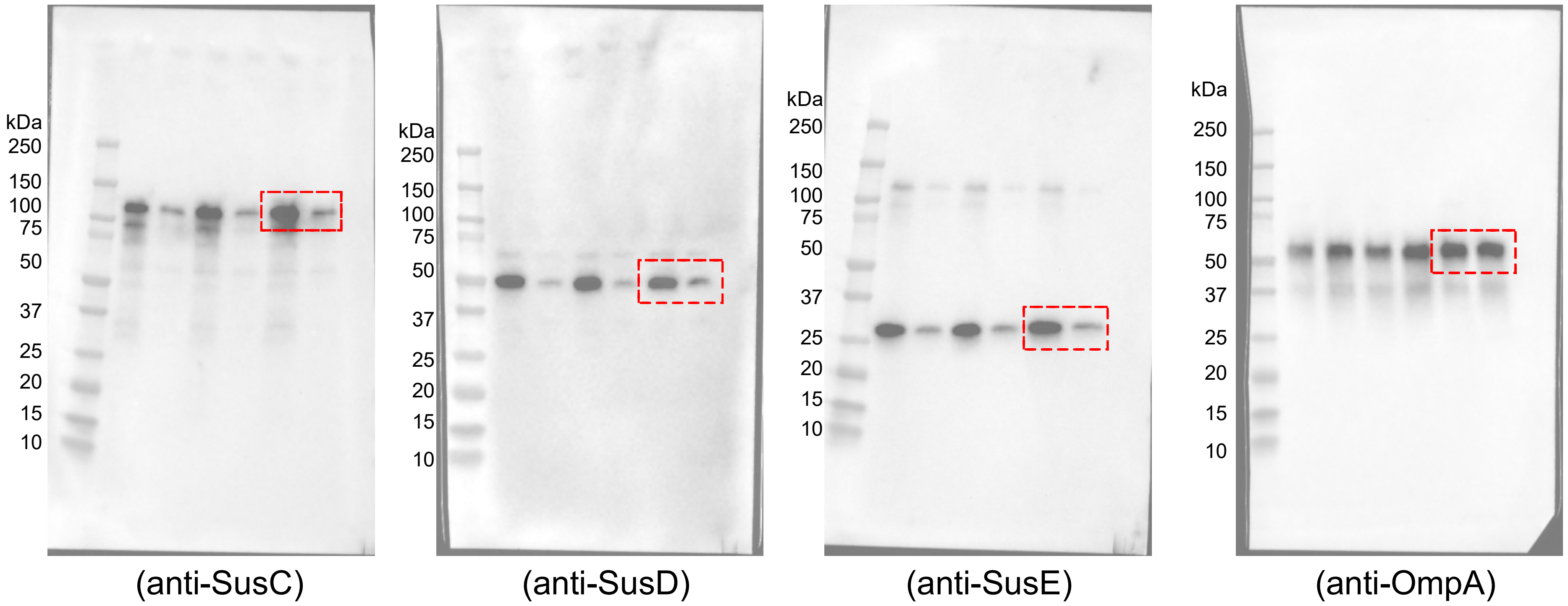
kDa
250
150
100
75
50
37
25
20
15
10
(anti-SusC)
kDa
250
150
100
75
50
37
25
20
15
10
(anti-SusD)
kDa
250
150
100
75
50
37
25
20
15
10
(anti-SusE)
kDa
250
150
100
75
50
37
25
20
15
10
(anti-OmpA)

Fig. 5 panel b

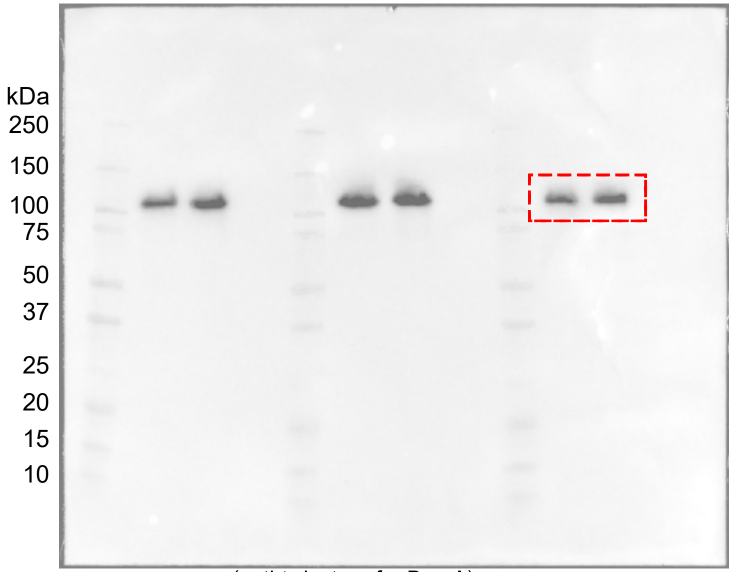

(anti-twinstrep for BamA)

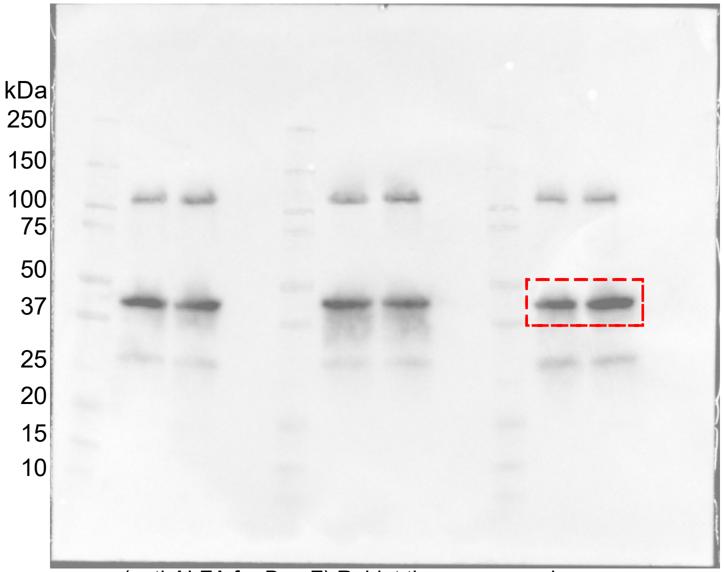

(anti-ALFA for BamF) Reblot the same membrane

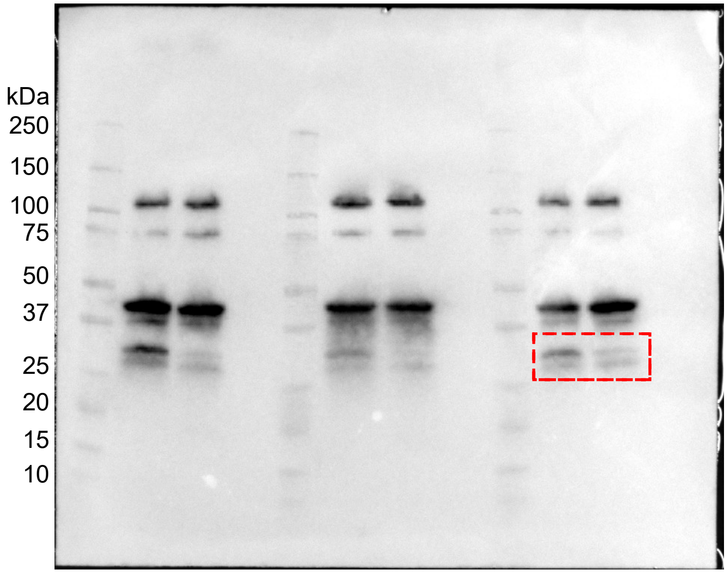

(anti-SprF) Reblot the same membrane

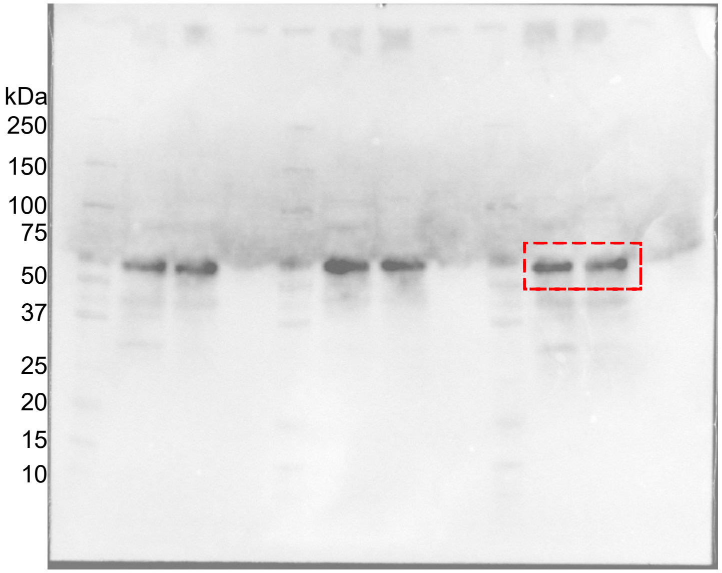

(anti-GroEL) Reblot the same membrane

Fig. 5 panel b

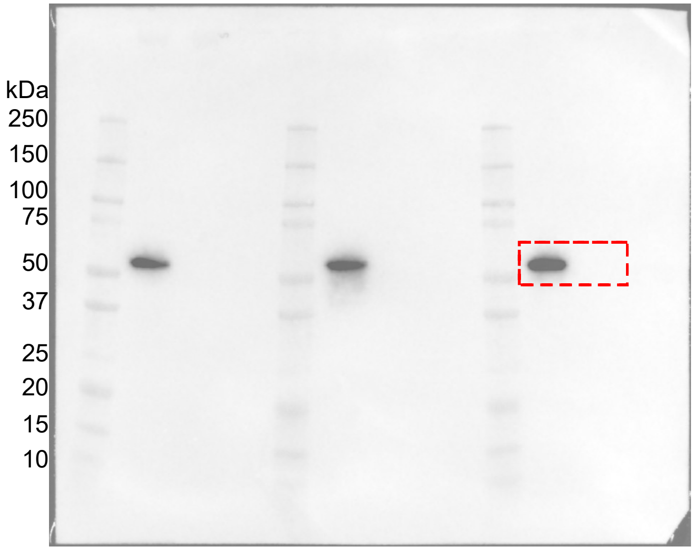

(anti-BamG)

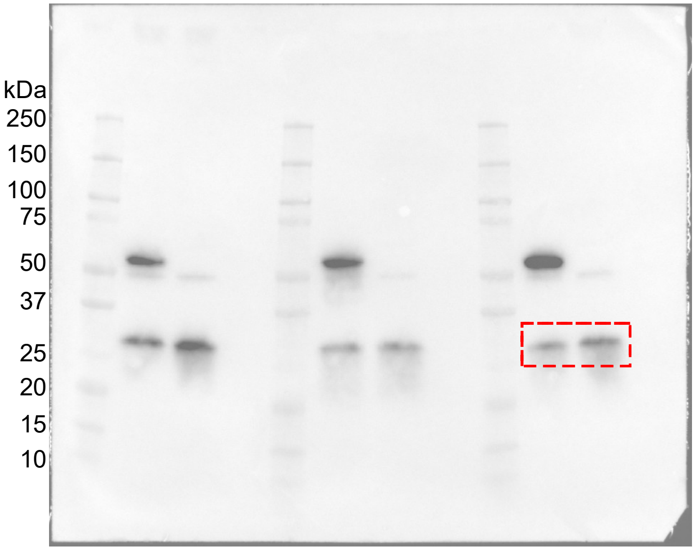

(anti-BamP)
reblot the same membrane of left

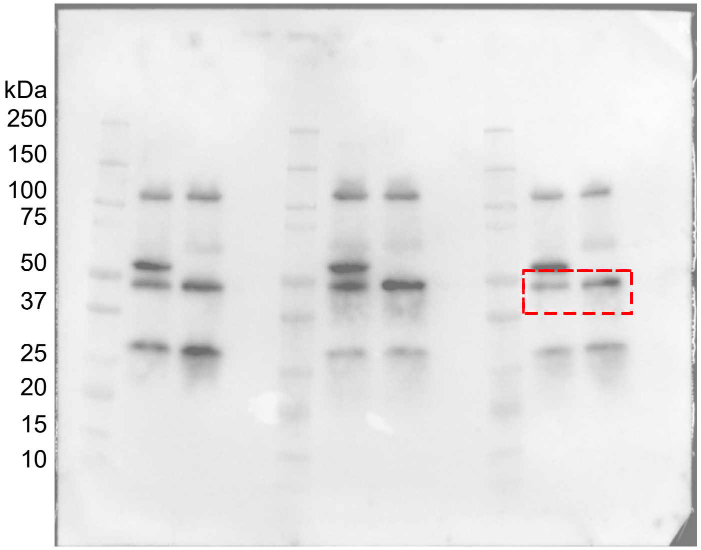

(anti-SkpA)
reblot the same membrane of left

Fig. 5 panel b

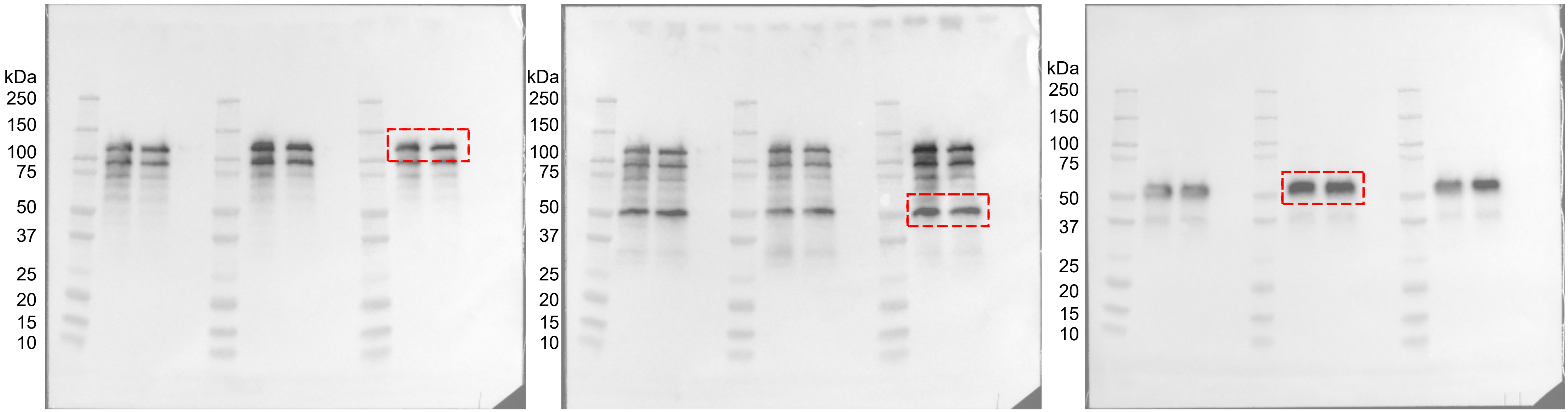

(anti-SusC)

(anti-SusD)
reblot the same membrane of left

(anti-OmpA)

ED Fig 3 panel b

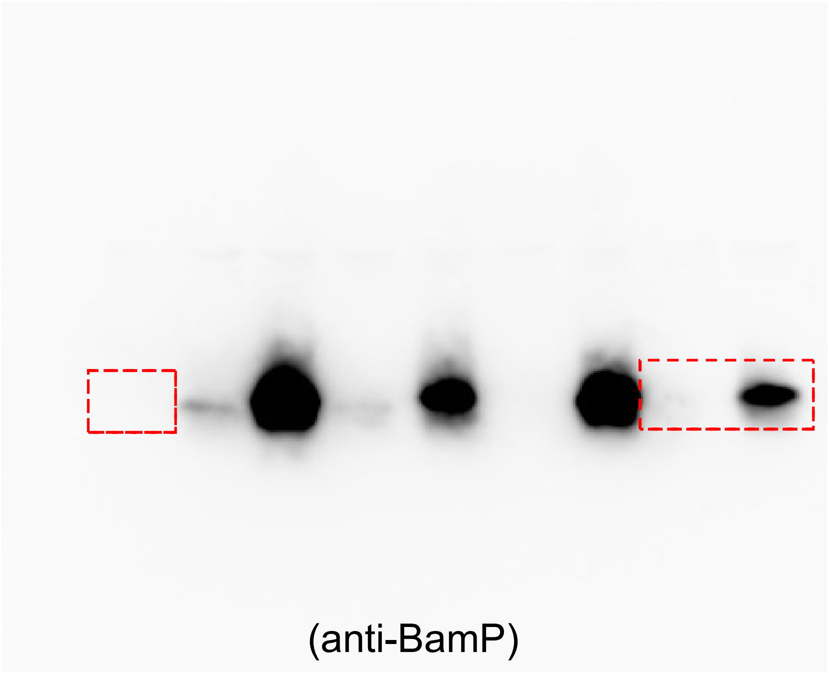

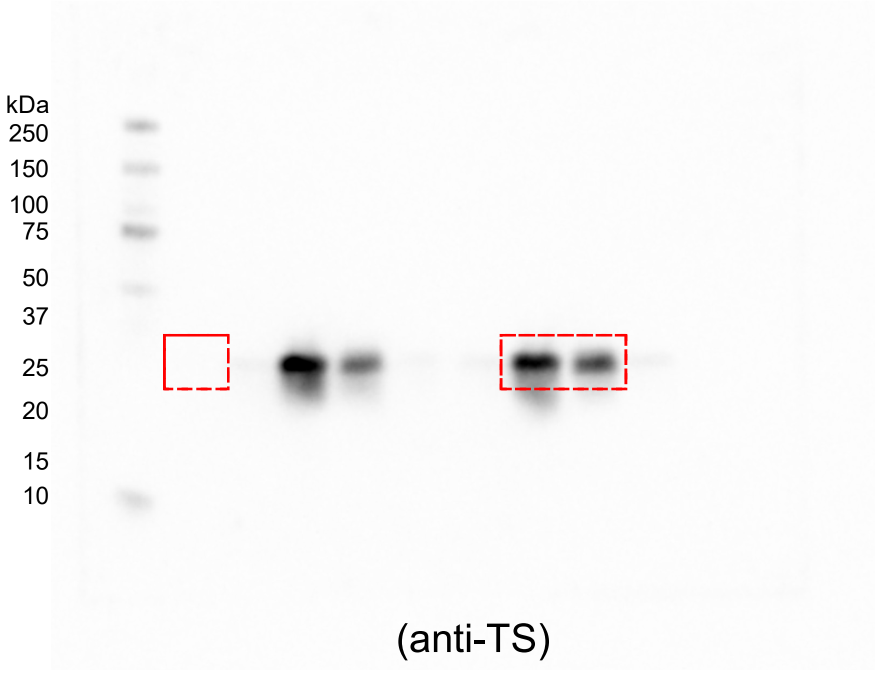

ED Fig 3 panel b

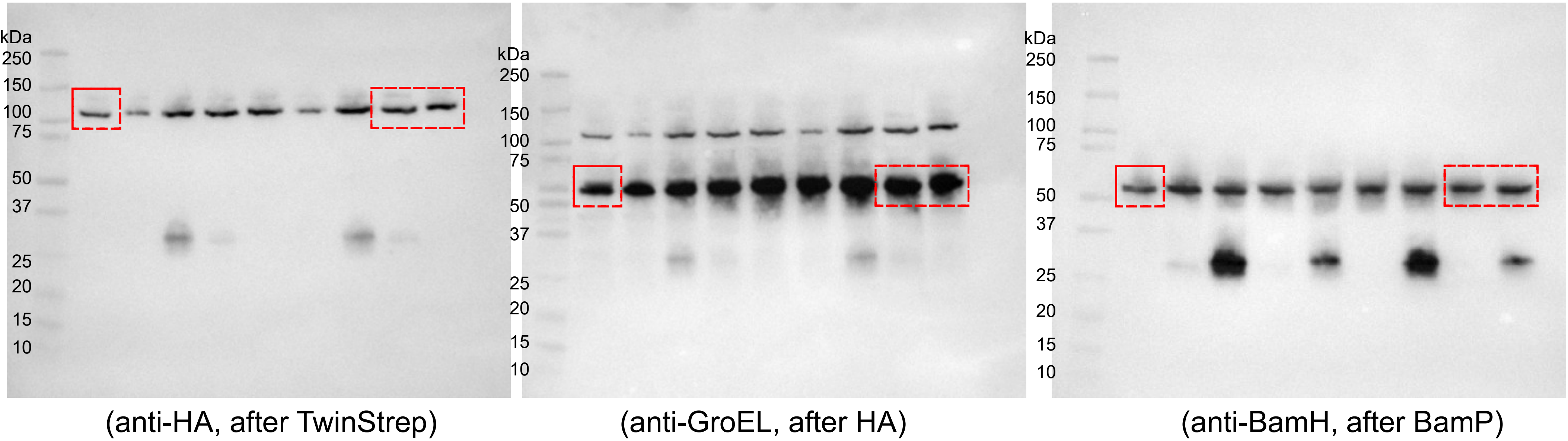

ED Fig 3 panel c

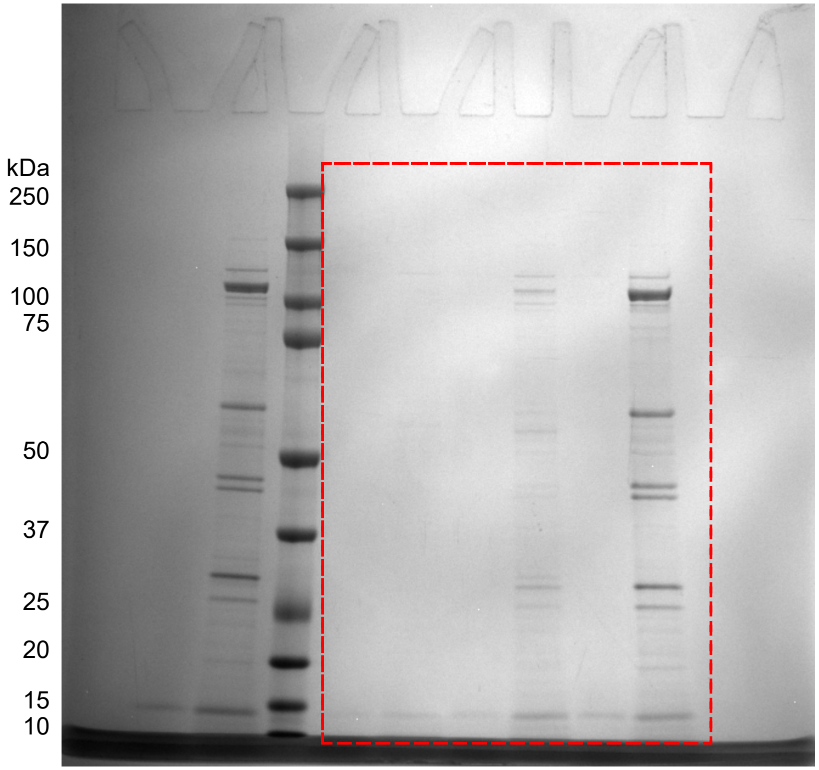

ED Fig 3 panel d

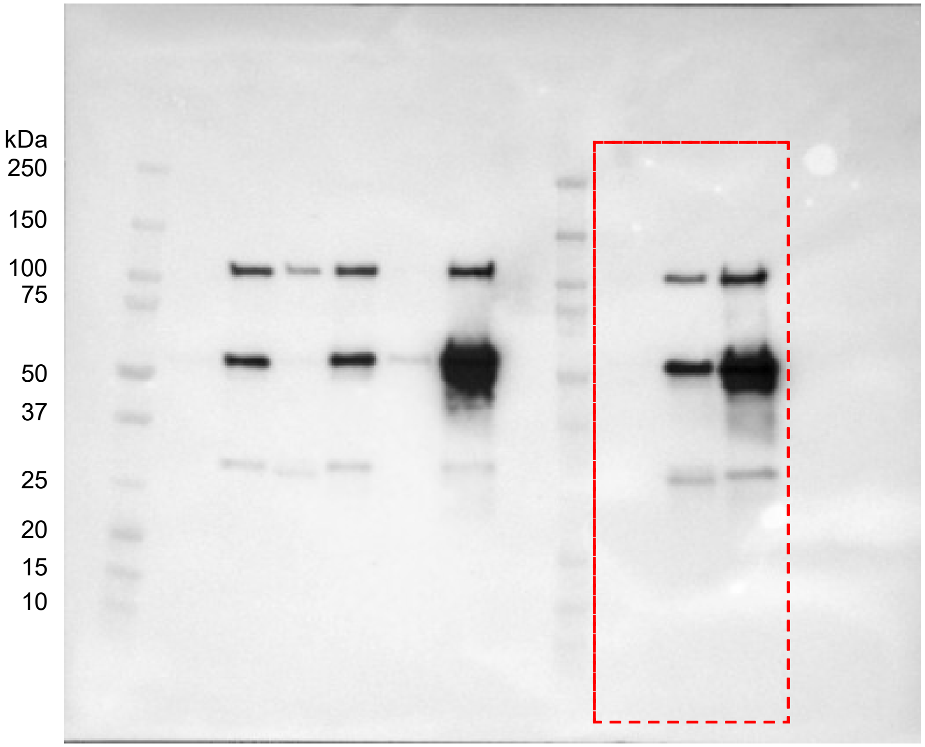

ED Fig. 4 panel a

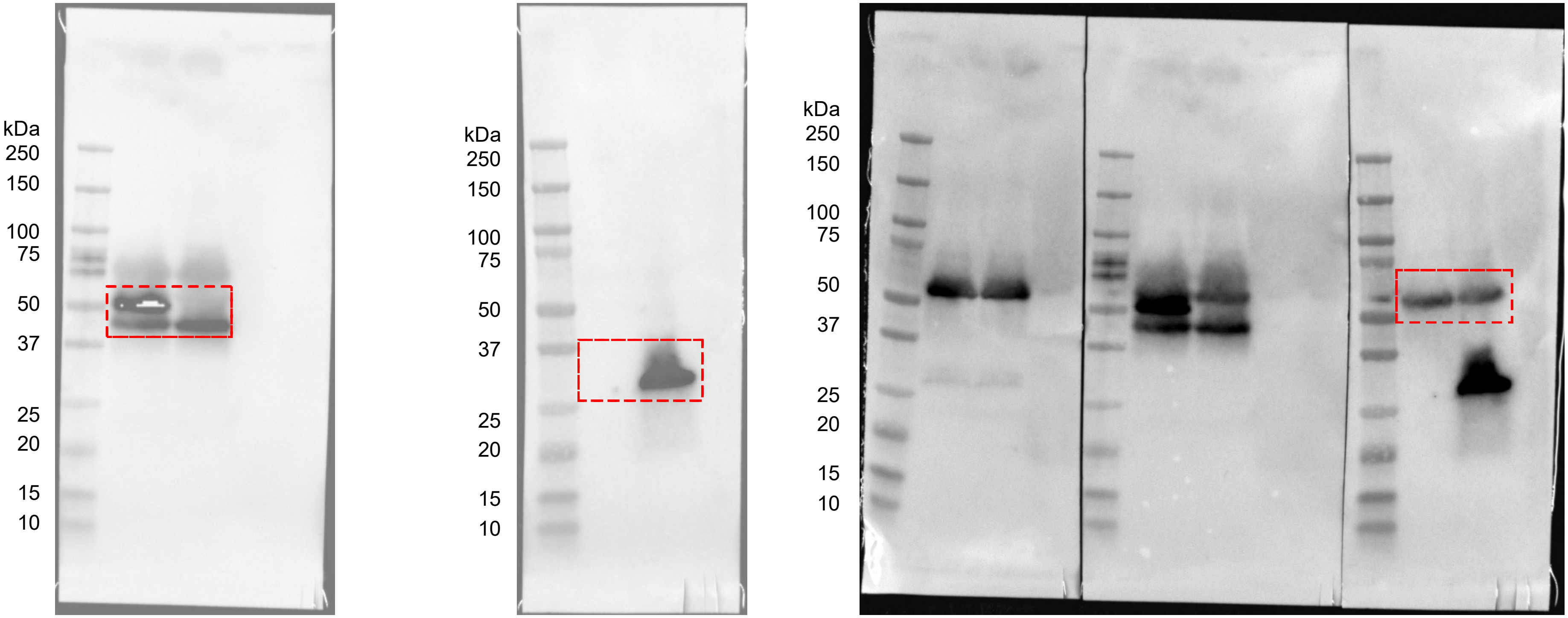

(anti-BamM)

(anti-BamP)

(anti-GroEL) reblot the membrane of left

ED 4a

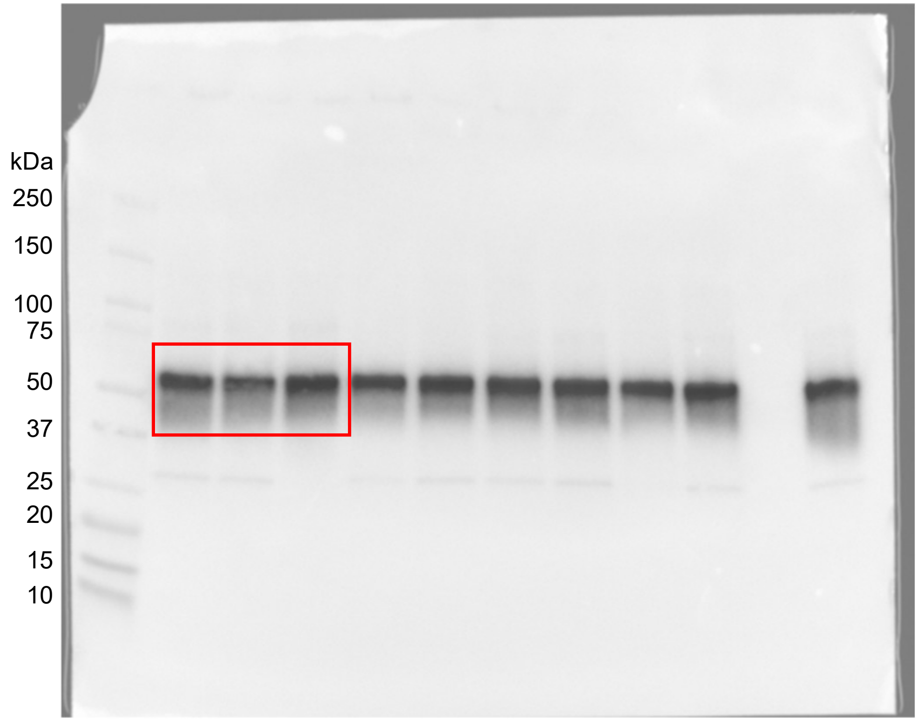

OmpA

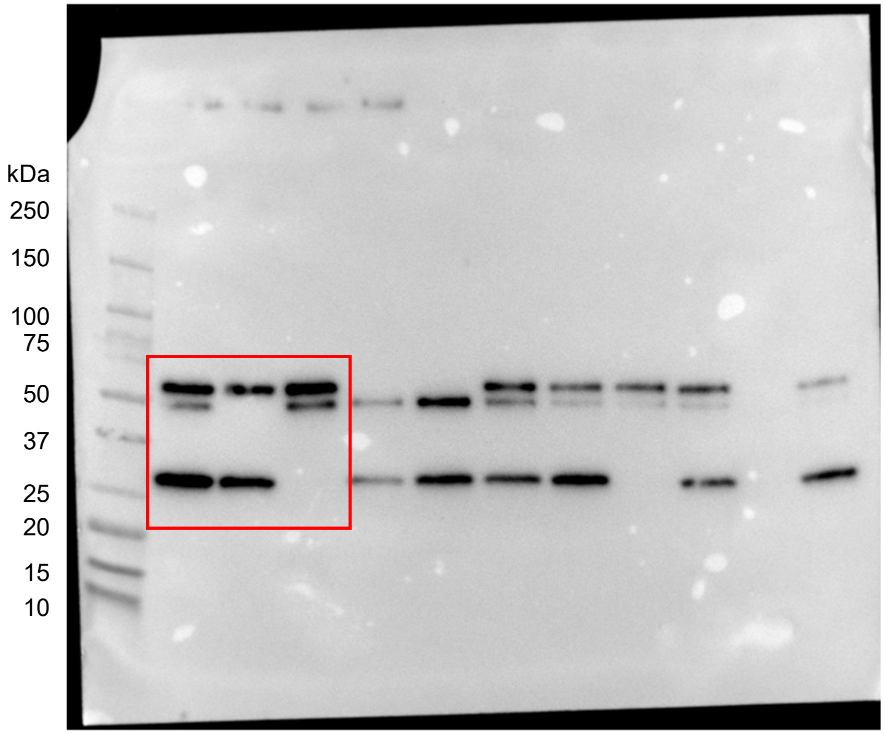

BamH
BamM
BamP

ED Fig. 5 panel a

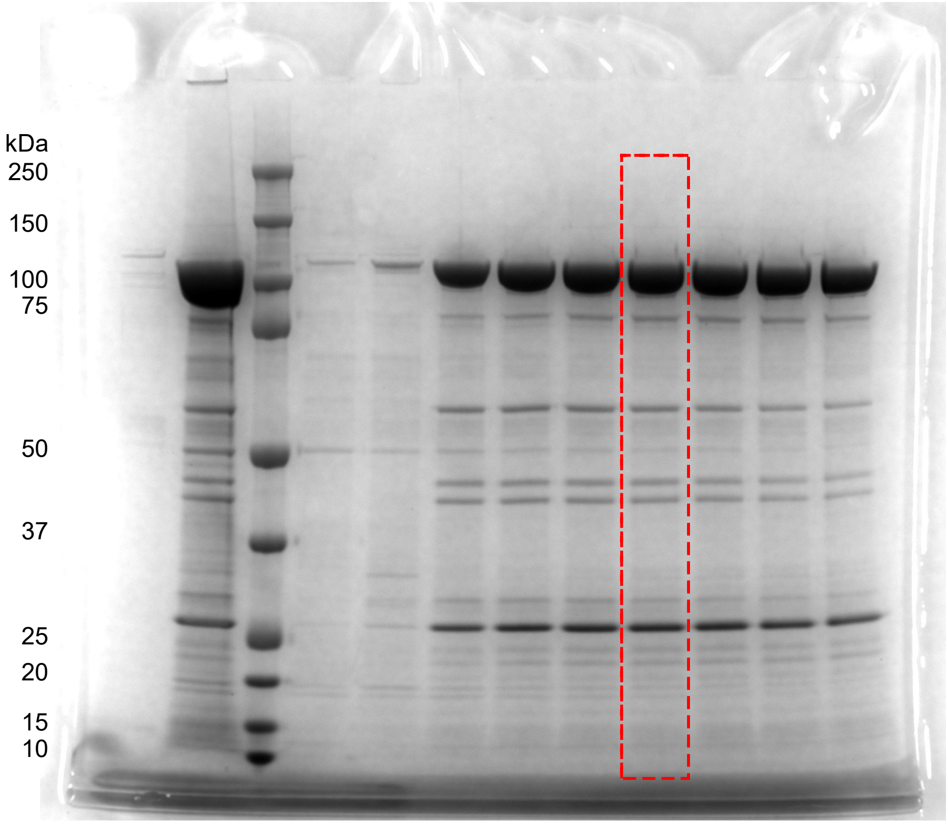

ED Fig. 7 panel d wt and *bamA[dep]*

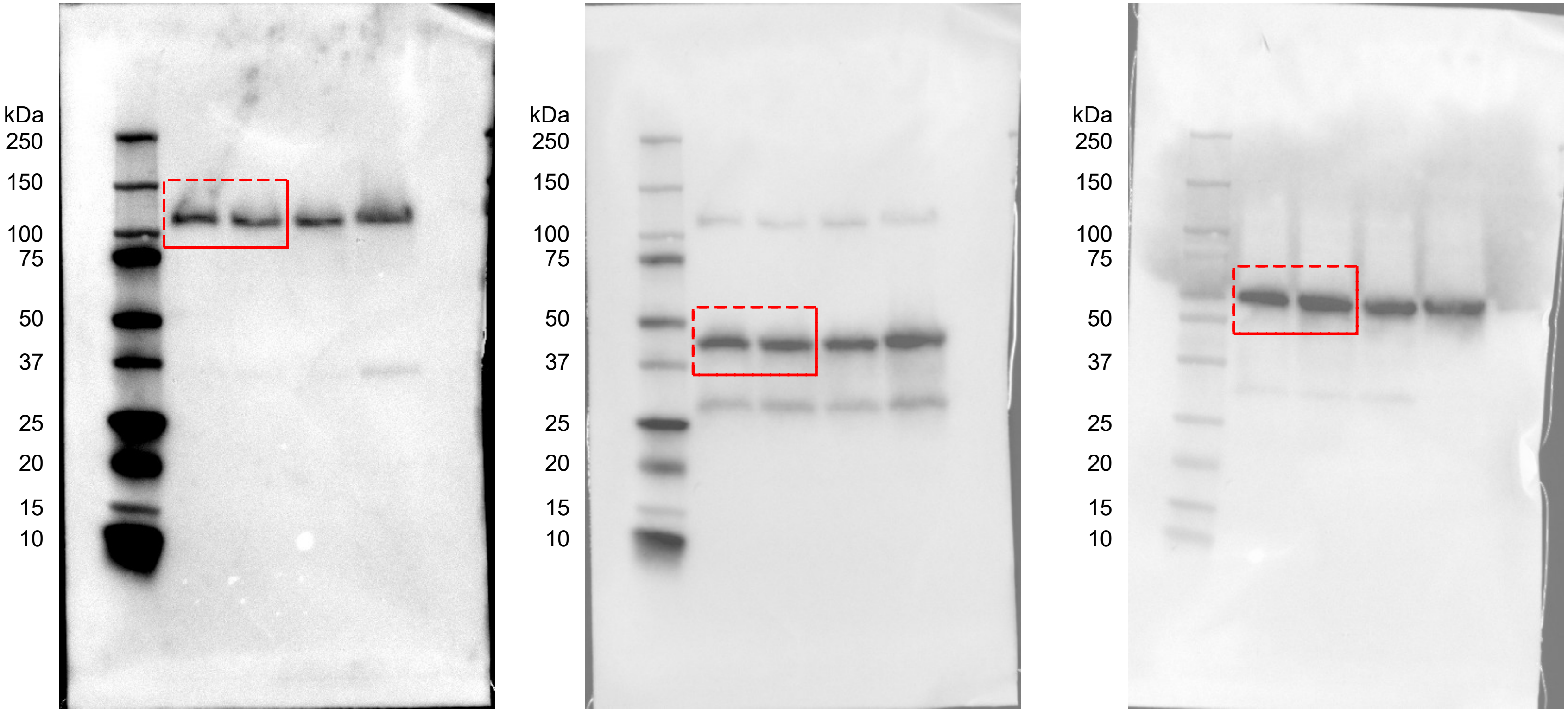

(anti-twinstrep for BamA)

(anti-ALFA for BamG)
Reblot the same membrane

(anti-GroEL)
Reblot the same membrane

ED Fig. 7 panel d wt and *bamA*^{dep}

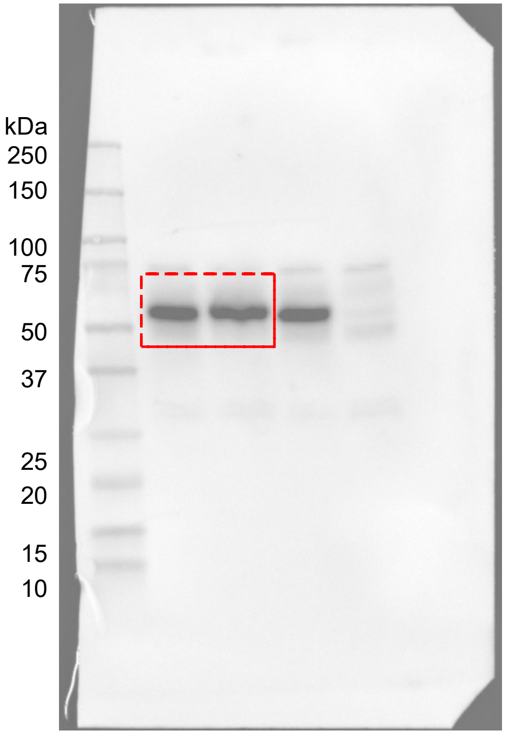

(anti-BamH)

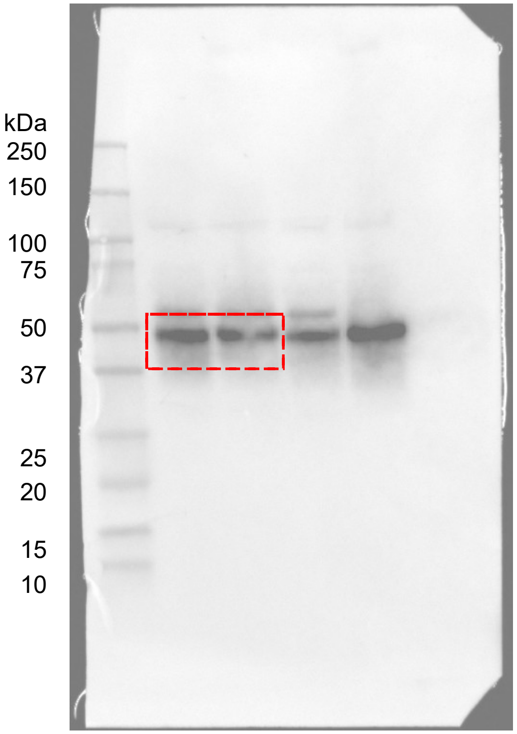

(anti-SkpA)
Reblot the same membrane of left

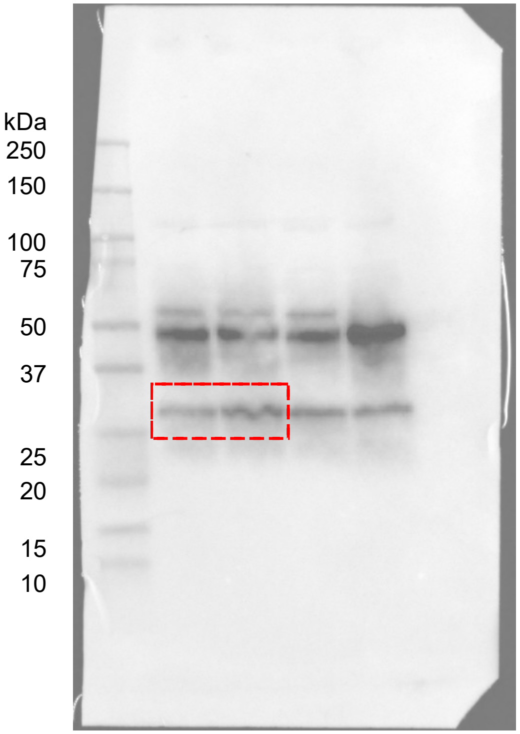

(anti-BamP)
Reblot the same membrane of left

ED Fig. 7 panel d wt and *bamG^dep*

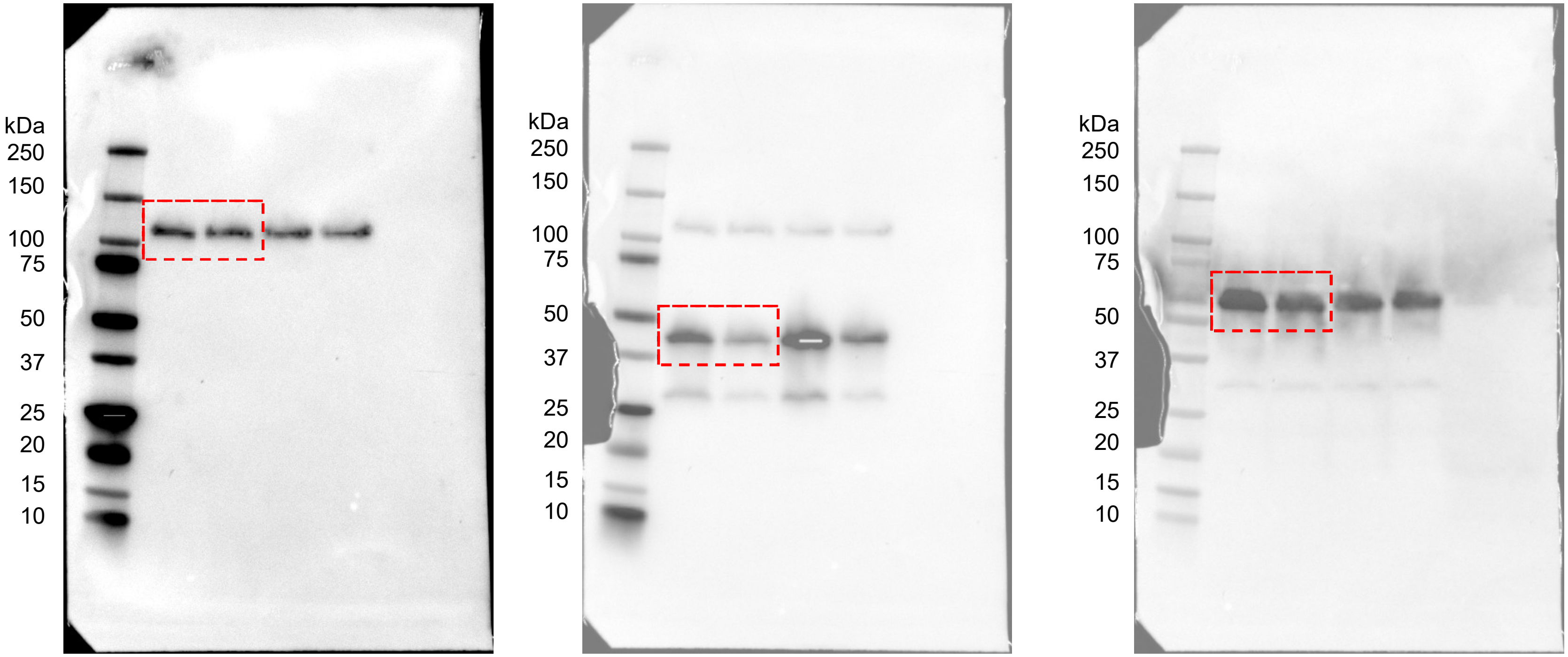

(anti-twinstrep for BamA)

(anti-ALFA for BamG)
Reblot the same membrane

(anti-GroEL)
Reblot the same membrane

ED Fig. 7 panel d wt and *bamG*^{dep}

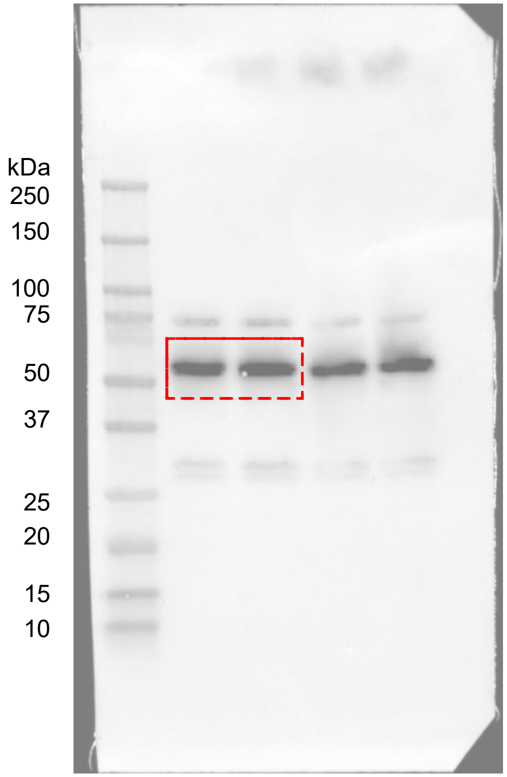

(anti-BamH)

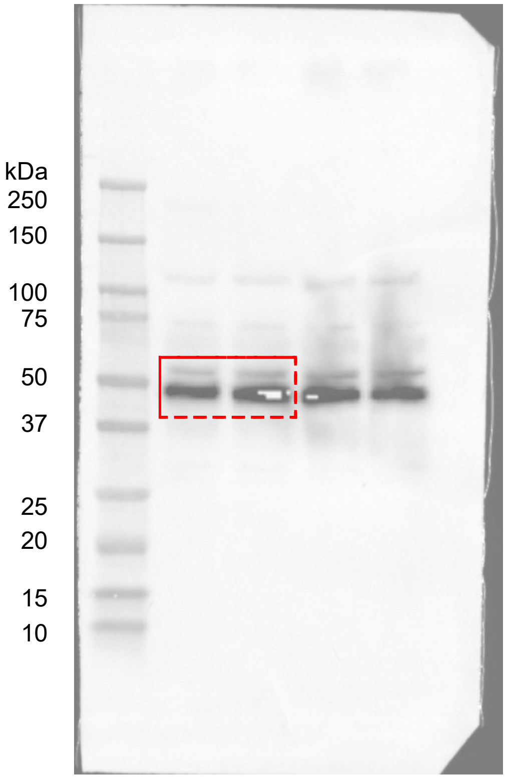

(anti-SkpA)
Reblot the same membrane of left

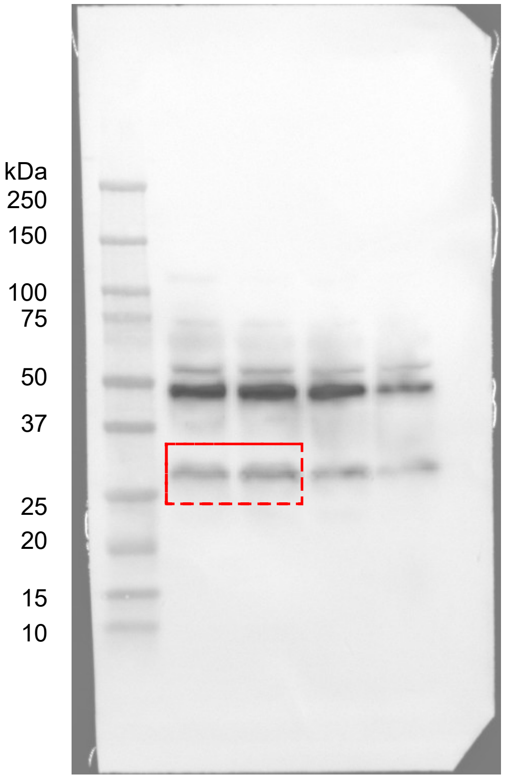

(anti-BamP)
Reblot the same membrane of left

ED Fig. 7 panel d wt and *bamH*^{dep}

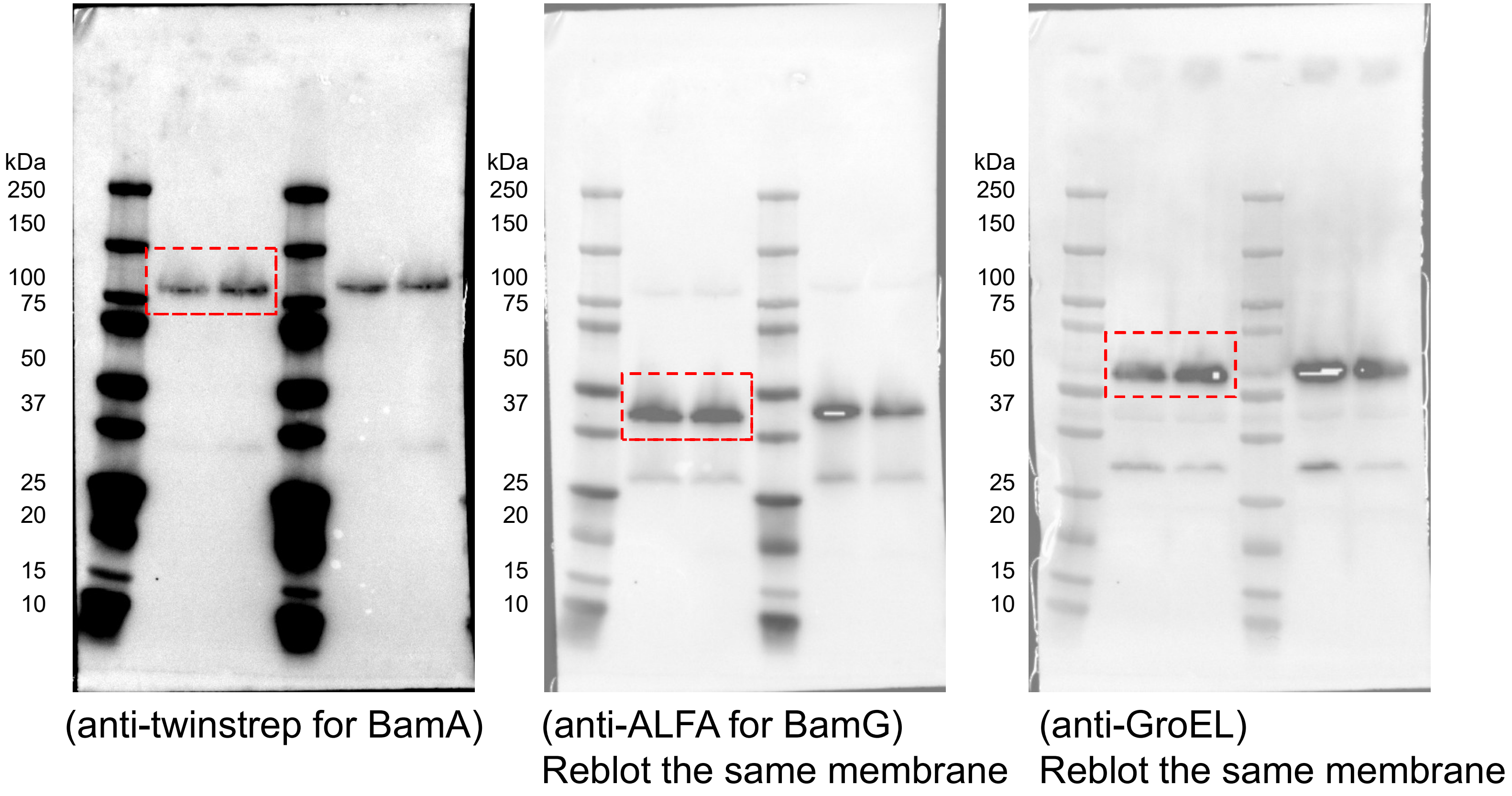

ED Fig. 7 panel d wt and *bamH*^{dep}

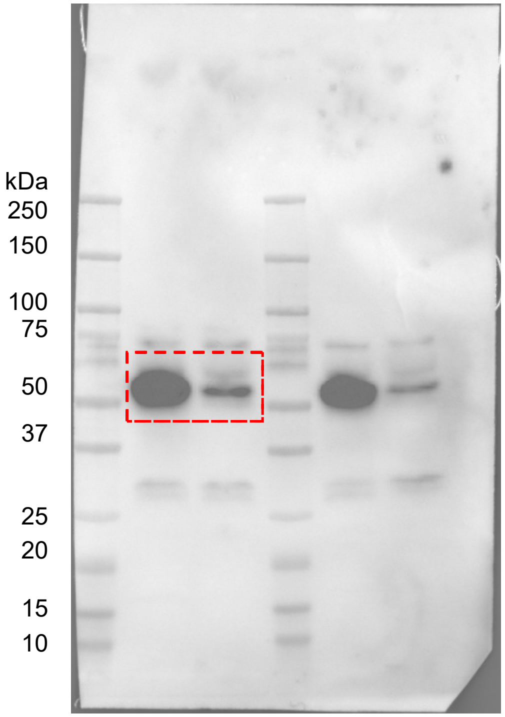

(anti-BamH)

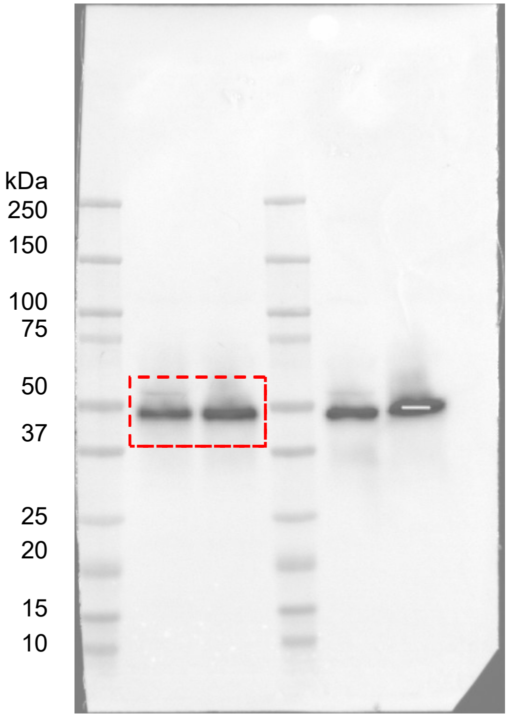

(anti-SkpA)
Reblot the same membrane of left

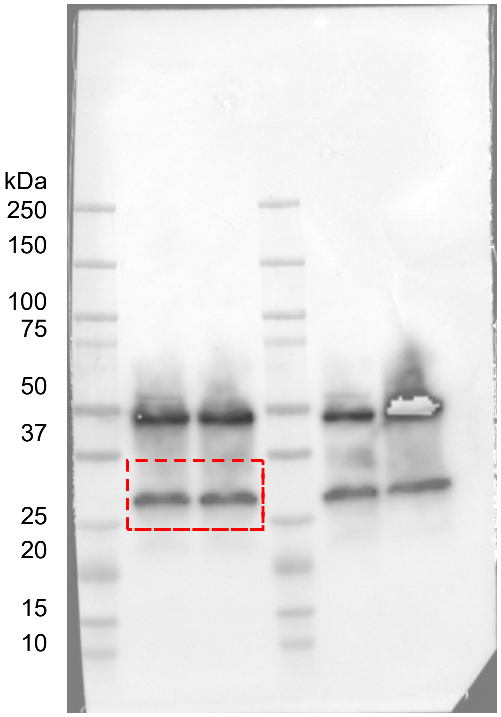

(anti-BamP)
Reblot the same membrane of left

ED Fig 7 f

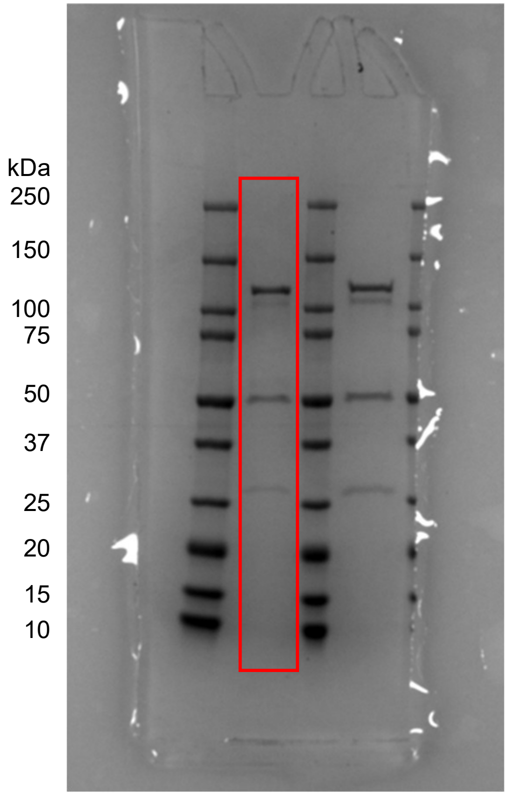

Coomassie blue

ED Fig. 7 panel g

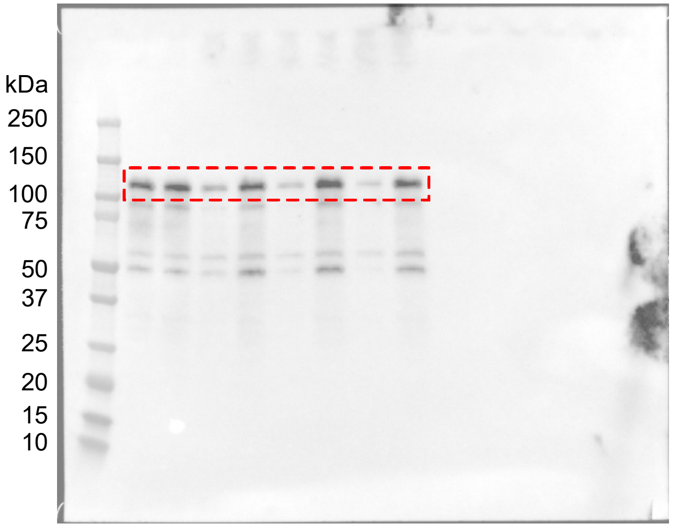

(anti-SusC) cells

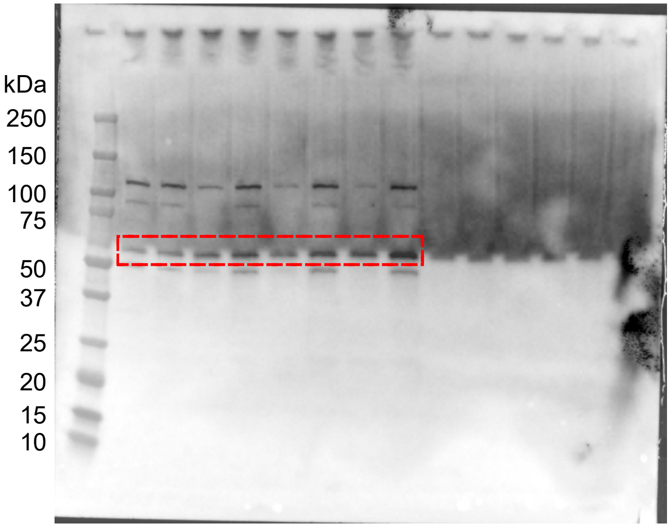

(anti-GroEL) cells
Reblot the same membrane of left

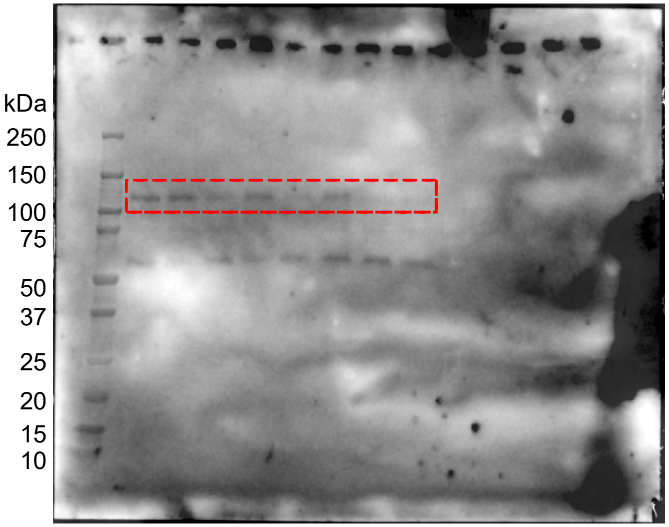

(anti-SusC) OMVs

ED Fig 7 h

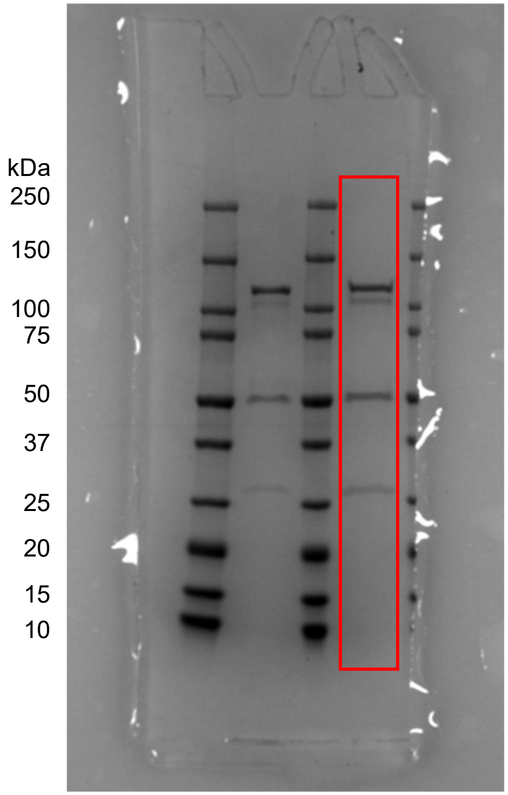

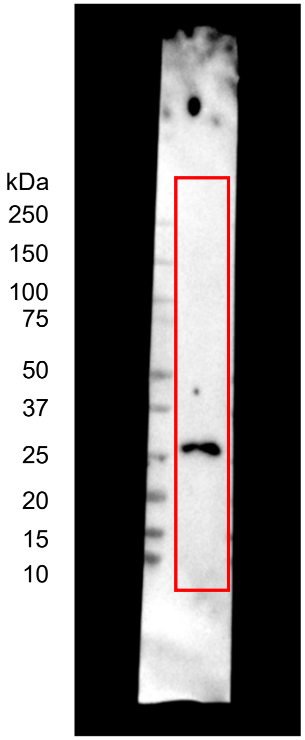

Coomassie blue

α-His

ED Fig 7 i

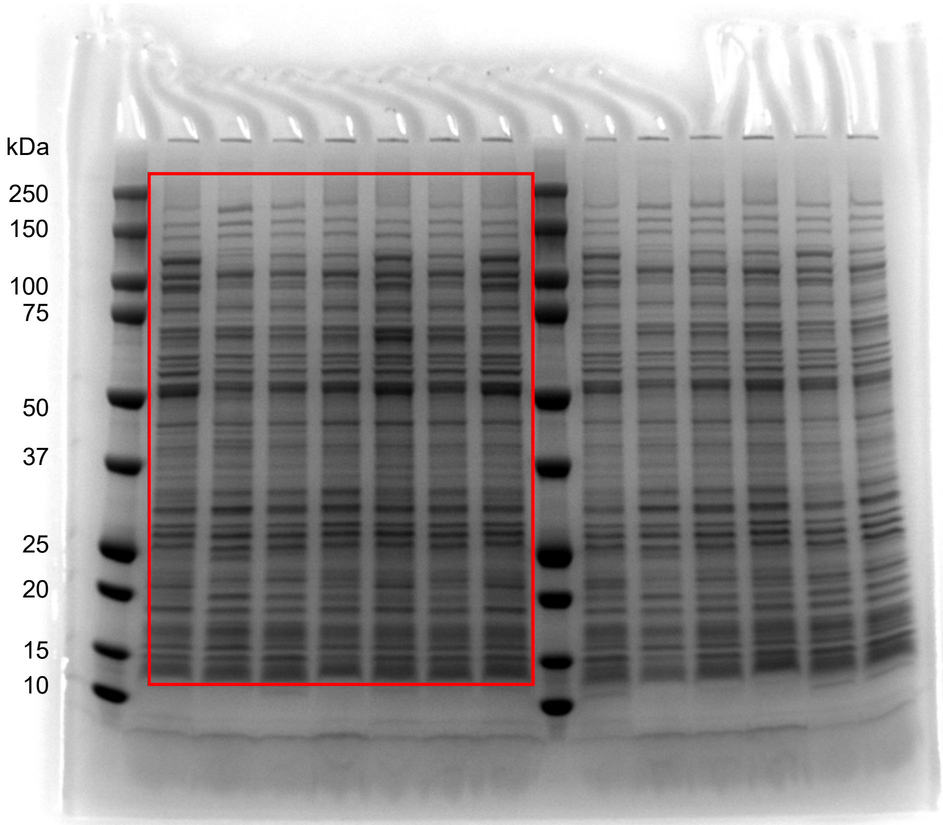

Left panel

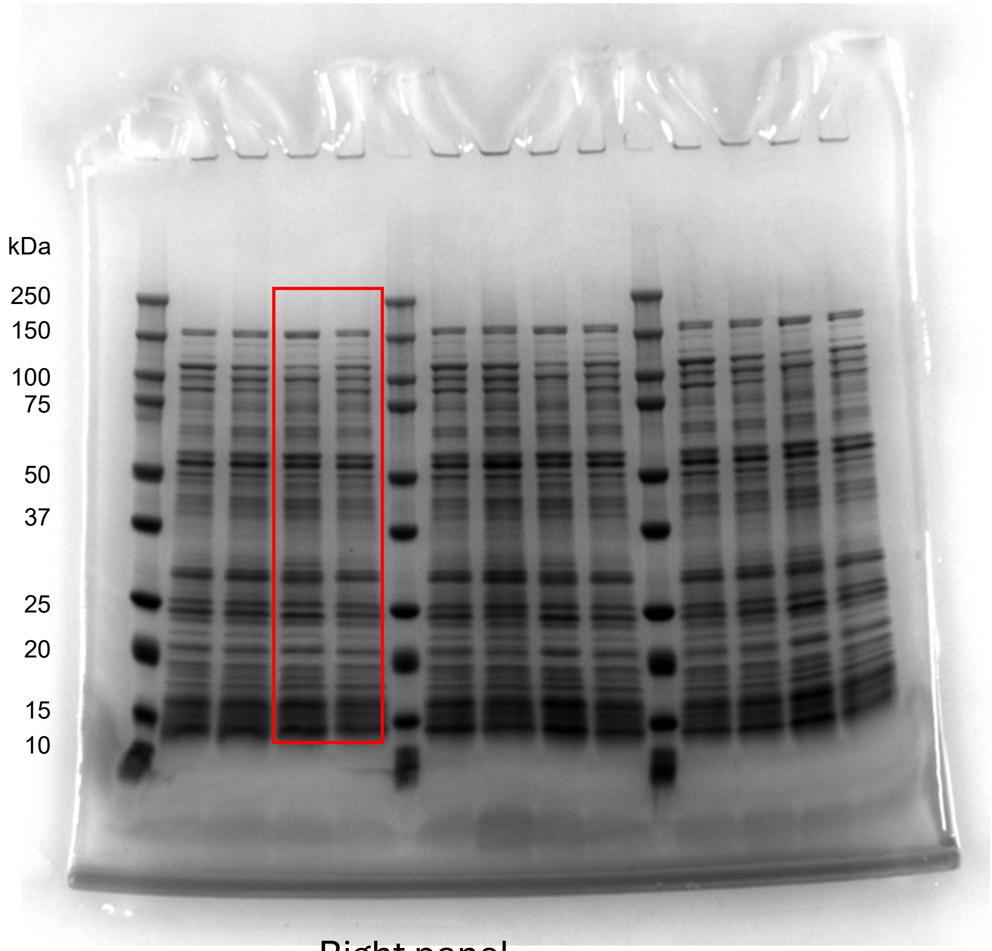

Right panel

ED Fig. 9 panel a

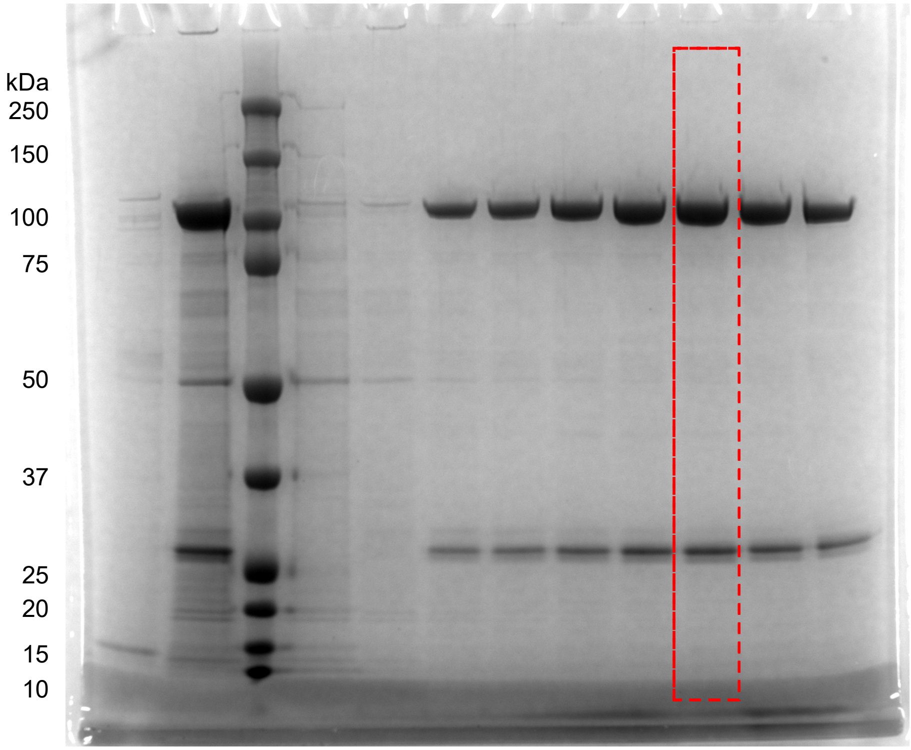

(BamM prep)

ED Fig. 9 panel b

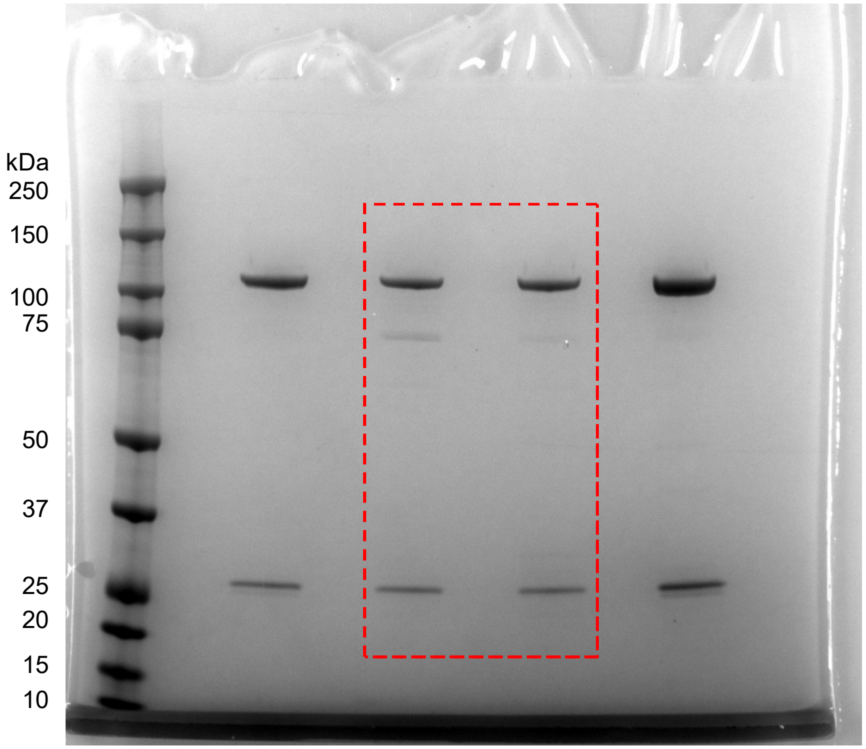

ED Fig. 9 panel d

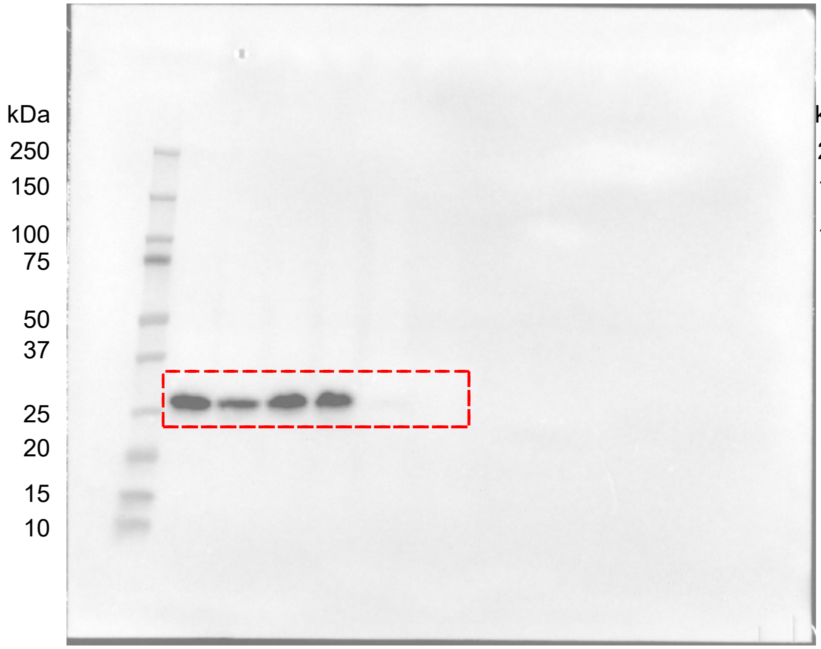

(anti-his for SusE)

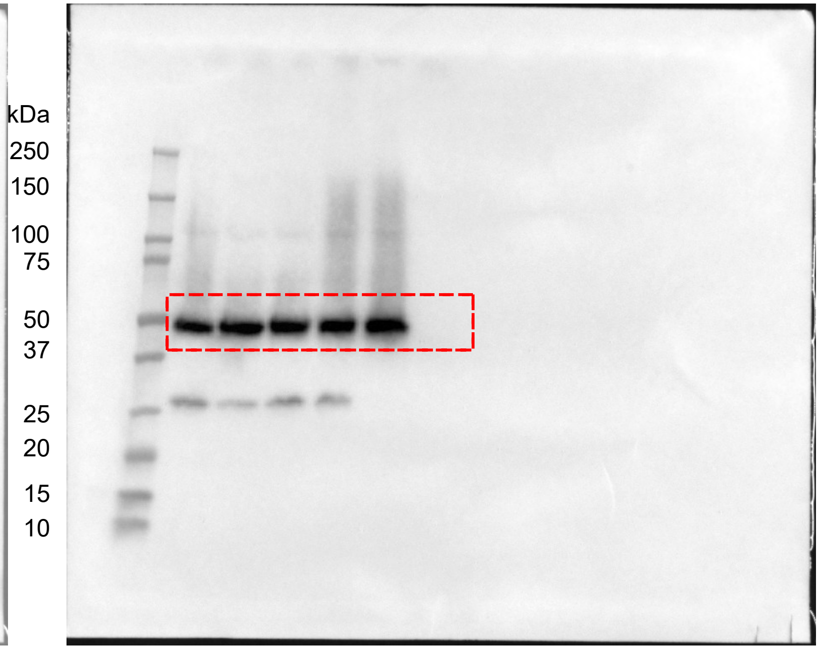

(anti-SkpA) reblot the membrane of left

ED Fig 9f

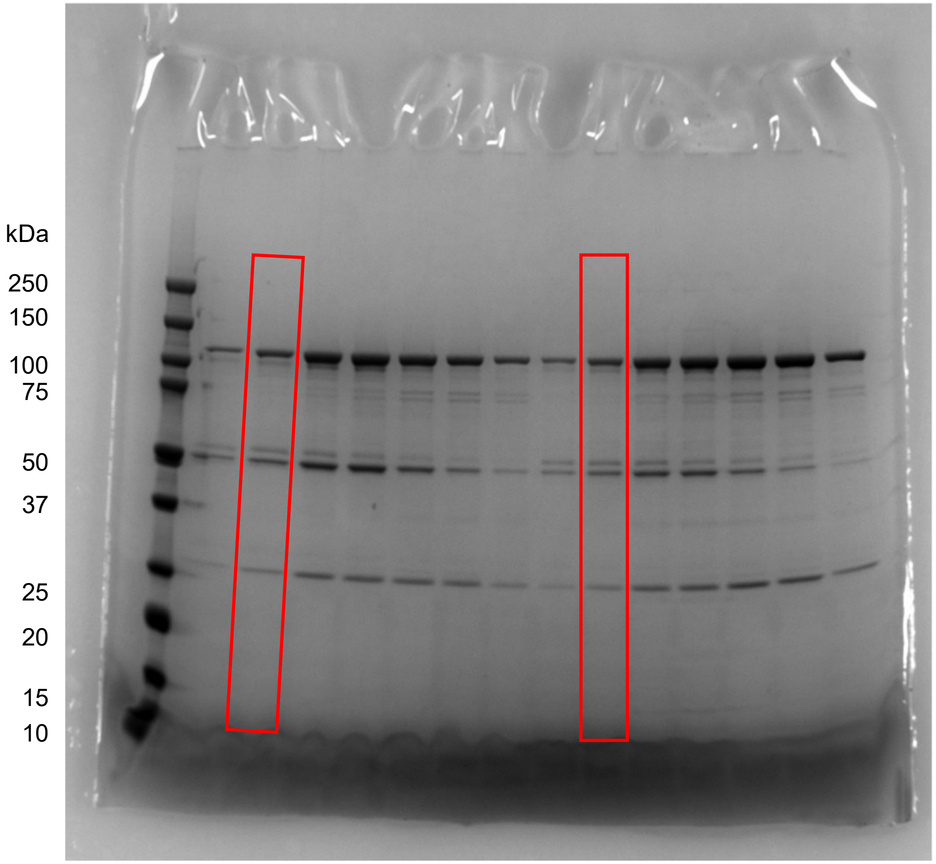

Supplement: Supplementary file 3 — Uncropped gels and immunoblots. See separate source data file Supplementary Fig. 1. [file 41586_2025_9532_MOESM3_ESM.pdf]
